# Loss of *MEN1* activates DNMT1 implicating DNA hypermethylation as a driver of MEN1 tumorigenesis

**DOI:** 10.18632/oncotarget.7279

**Published:** 2016-02-09

**Authors:** Ziqiang Yuan, Carmen Sánchez Claros, Masako Suzuki, Elaine C. Maggi, Justin D. Kaner, Noah Kinstlinger, Jolanta Gorecka, Thomas J. Quinn, Rula Geha, Amanda Corn, Jessica Pastoriza, Qiang Jing, Asha Adem, Hao Wu, Girum Alemu, Yi-Chieh Du, Deyou Zheng, John M. Greally, Steven K. Libutti

**Affiliations:** ^1^ Department of Surgery, Albert Einstein College of Medicine, Bronx, New York, USA; ^2^ Department of Genetics, Albert Einstein College of Medicine, Bronx, New York, USA; ^3^ Department of Medicine, Albert Einstein College of Medicine, Bronx, New York, USA; ^4^ Department of Pathology and Lab Medicine, Weill Cornell Medical College, New York, New York, USA; ^5^ Department of Neurology, Albert Einstein College of Medicine, Bronx, New York, USA; ^6^ Department of Neuroscience, Albert Einstein College of Medicine, Bronx, New York, USA; ^7^ Department of Pediatrics, Albert Einstein College of Medicine, Bronx, New York, USA

**Keywords:** MEN1, global DNA methylation, HELP-tagging, DNMT1, Sox/Wnt/β-catenin signaling pathway

## Abstract

Multiple endocrine neoplasia type 1 (MEN1) syndrome results from mutations in the *MEN1* gene and causes tumor formation via largely unknown mechanisms. Using a novel genome-wide methylation analysis, we studied tissues from MEN1-parathyroid tumors, *Men1* knockout (KO) mice, and *Men1* null mouse embryonic fibroblast (MEF) cell lines. We demonstrated that inactivation of menin (the protein product of *MEN1*) increases activity of DNA (cytosine-5)-methyltransferase 1 (DNMT1) by activating retinoblastoma-binding protein 5 (Rbbp5). The increased activity of DNMT1 mediates global DNA hypermethylation, which results in aberrant activation of the Wnt/β-catenin signaling pathway through inactivation of *Sox* regulatory genes. Our study provides important insights into the role of menin in DNA methylation and its impact on the pathogenesis of MEN1 tumor development.

## INTRODUCTION

Multiple endocrine neoplasia type 1 (MEN1) is a familial cancer syndrome characterized by tumors of the endocrine glands, including the parathyroid, anterior pituitary, and endocrine pancreas. MEN1 results from germline mutations in the tumor suppressor gene *MEN1* (protein product menin). Somatic mutations in *MEN1* are also frequently identified in sporadic parathyroid adenomas, insulinomas, gastrinomas, non-functional pancreatic neuroendocrine tumors, and lung carcinoids [1–5]. Menin is an important multifunctional transcriptional modulator that binds multiple proteins, including: host cell factor 2 (HCF2) [6], retinoblastoma-binding protein 5 (Rbbp5) [7], and mixed-lineage leukemia (MLL1/MLL2) [8–10]. Menin, Rbbp5, and MLL co-localize with the promoters of thousands of genes in the human pancreatic islet and the cell lines HeLa and HepG2 to act as a transcriptional activator [7]; additionally, menin alters DNA repair, cell proliferation, and apoptosis [11–16].

Epigenetic alterations are important in tumorigenesis and include histone post-transcriptional modifications, direct DNA methylation, chromatin organization, and non-coding regulatory RNA [17]. Menin uses epigenetic regulation to control gene expression patterns [9, 18–22]. For example, menin is essential in the MLL1 and MLL2 histone methyltransferase complexes, which increase histone methylation [9, 18–20]. Inactivation of menin was found to reduce binding to protein arginine N-methyltransferase 5 (PRMT5), ultimately decreasing Gas1 expression in MEN1 tumors [23]. Frequent DNA hypermethylation of cyclin-dependent kinase inhibitor 2A (CDKN2A), Ras association domain family member 1 (RASSF1A), and adenomatous polyposis coli (APC) promoters has been reported in MEN1-associated tumors [21, 22]. While these studies focused on individual genes, a comprehensive genome-wide DNA methylation study of MEN1-related tumors has not been performed. Genome-wide approaches have shown aberrantly methylated regions assist in the neoplastic processes [24–28].

Recently, our group developed and validated a novel high-throughput DNA methylation assay, HpaII tiny fragment enrichment by ligation-mediated PCR (HELP)-tagging, utilizing massively parallel sequencing for measuring global DNA methylation [29, 30]. In the present study, we performed the first genome-wide analysis of quantitative global DNA methylation in MEN1 tumors. We utilized a large tissue biorepository of human tumor samples and validated our findings using knockout (KO) mice and cell line models. We identified a possible molecular mechanism elucidating how inactivating menin results in global DNA hypermethylation in MEN1-related tumors. Finally, we identified Sox-mediated regulation of Wnt/β-catenin signaling as a mechanism contributing to MEN1-related tumor formation.

## RESULTS

### Global parathyroid DNA hypermethylation in MEN1 patients

**Figure 1:**
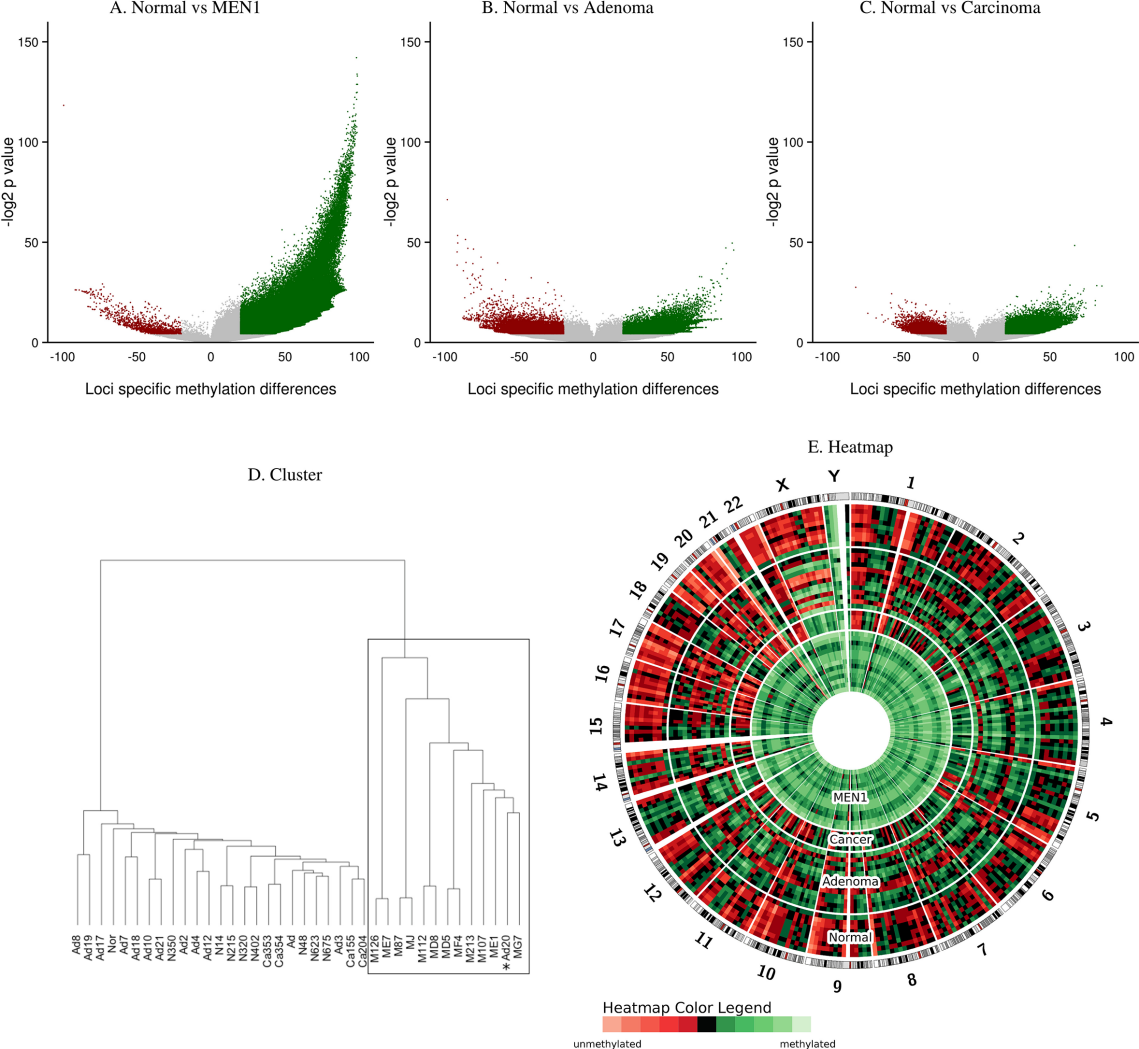
Global DNA methylation in MEN1-parathyroid tumors Volcano plots comparing the DNA methylation between parathyroid tumor samples and controls are on the x-axis and the −log2 of the corresponding *P* values of those mean differences are on the y-axis (**A**–**C**). The green dots represent significantly hypermethylated loci whereas the red dots represent hypomethylated ones. Loci in MEN1-parathyroid tumor cases are significantly more methylated when compared to normal parathyroid tissues (A). Hierarchical Cluster showing high correlation of methylated loci in the MEN1 group (*: single sporadic adenoma that segregated with the MEN1 group; Ad: parathyroid adenoma; Ca: parathyroid carcinoma; M: MEN1-parathyroid tumor; N: Normal parathyroid tissues) (**D**). Circular heatmap representation of DNA methylation levels for parathyroid adenoma (Adenoma), parathyroid carcinoma (Cancer), MEN1-parathyroid tumors (MEN1), and normal parathyroid (Normal) (**E**). DNA methylation levels from each patient were averaged in 10 Mbp genomic windows. The heatmap indicates global DNA hypermethylation in MEN1 patients.

DNA methylation analysis was performed with HELP-tagging plus massively parallel sequencing to detect the CpG methylation status of approximately 2.0 million CCGG loci distributed throughout the genome. There was significantly increased genome-wide DNA methylation in MEN1-parathyroid tumors compared to normal human parathyroid tissues, sporadic parathyroid adenomas, and parathyroid cancers (Figure 1). While 466,950 loci were significantly hypermethylated in MEN1-parathyroid tumors (Figure 1A), only 48,162 and 27,169 loci were significantly hypermethylation in parathyroid adenomas (Figure 1B) and parathyroid carcinomas (Figure 1C) respectively, when compared to normal parathyroids. Out of 275,340 loci located in promoter regions (2000 of the target genes are shown in Supplementary Table 1), 167,988 loci were significantly hypermethylated, of those, 3772 loci were in tumor suppressor genes (Supplementary Figure 1A and Supplementary Table 2). We also analyzed the promoter regions of the Polycomb genes, which are related to cancer development (Supplementary Table 3). Upon identical examination of the gene body region, we identified 134,101 loci were significantly hypermethylated out of 804,491 loci (Supplementary Figure 1B). These findings suggest increased DNA methylation in MEN1-parathyroid tumors is a genome-wide event.

Global DNA methylation was further validated with MassArray [29] and Pyrosequencing techniques, by their high correlation coefficients (Supplementary Table 4). Hierarchical clustering (Figure 1D) revealed unique nodal clustering, with sporadic adenomas, carcinomas, and normal samples clustering separately. Interestingly, a single sporadic parathyroid adenoma clustered together with the MEN1-parathyroid tumor group and showed a global hypermethylation phenotype as well (Figure 1D). DNA sequencing of this particular case revealed a missense mutation at codon 338 (Leu338Pro) in the *MEN1* gene (exon 6) (Supplementary Table 5). This missense mutation is located in the functional domain responsible for Jun D interaction. We did not find this *MEN1* missense mutation to be reported in the normal population from the HapMap database (www.hapmap.ncbi.nih.gov). Other sporadic parathyroid adenoma tissues were screened for *MEN1* gene mutations by direct sequencing and no additional missense or truncating mutations were found (Supplementary Table 5). Heatmap analysis also illustrated global DNA hypermethylation in MEN1 patients (Figure 1E).

### Upregulation of DNA (cytosine-5)-methyltransferase 1 (DNMT1) expression in human MEN1-parathyroid tumors and endocrine tumors of the pancreas and parathyroid from *Men1* KO mice

**Figure 2:**
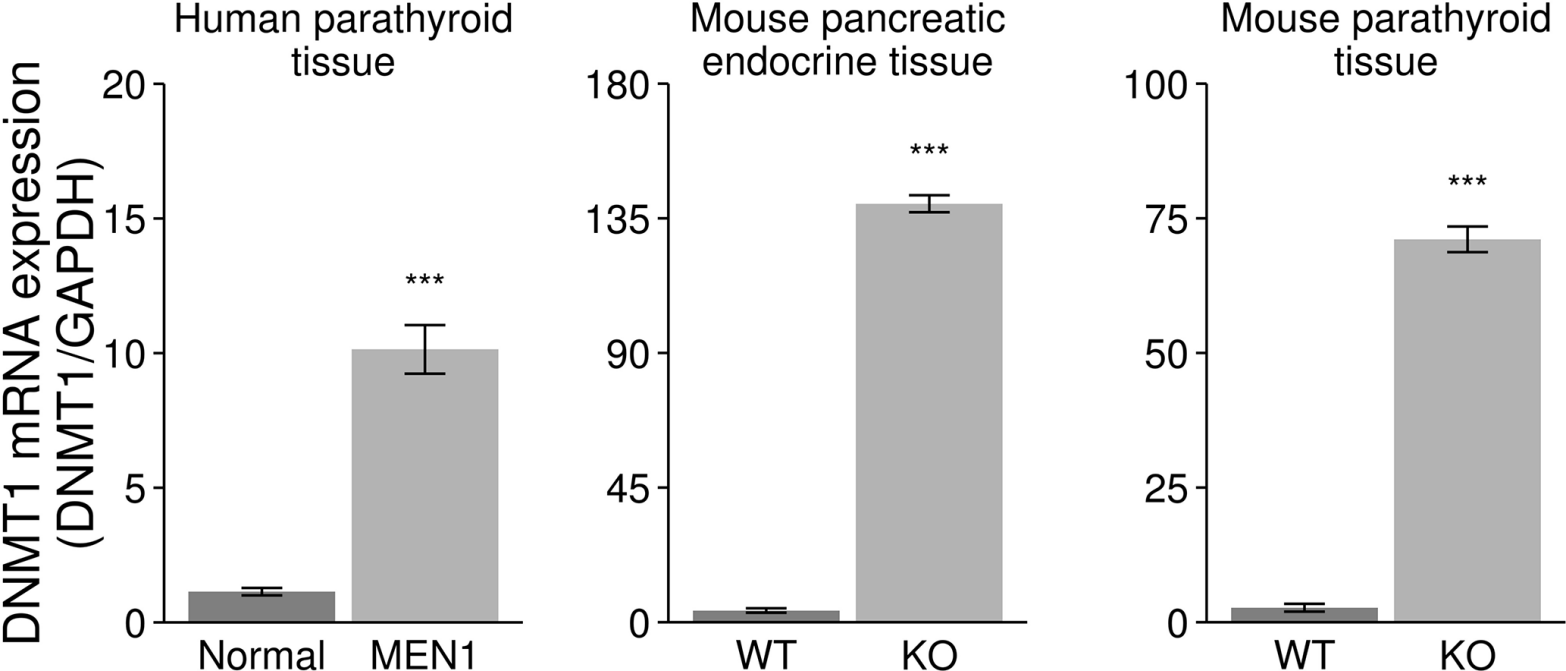
Increased expression of DNMT1 in the endocrine tumor tissues from human MEN1 parathyroids and *Men1* KO mice DNMT1 mRNA was significantly increased in the endocrine parathyroid tumors from MEN1 patients (MEN1) and pancreas and parathyroid from *Men1* KO mice (KO) compared to normal endocrine tissues from human and control mice (WT) by real-time RT-PCR assay (***P < 0.001).

**Figure 3:**
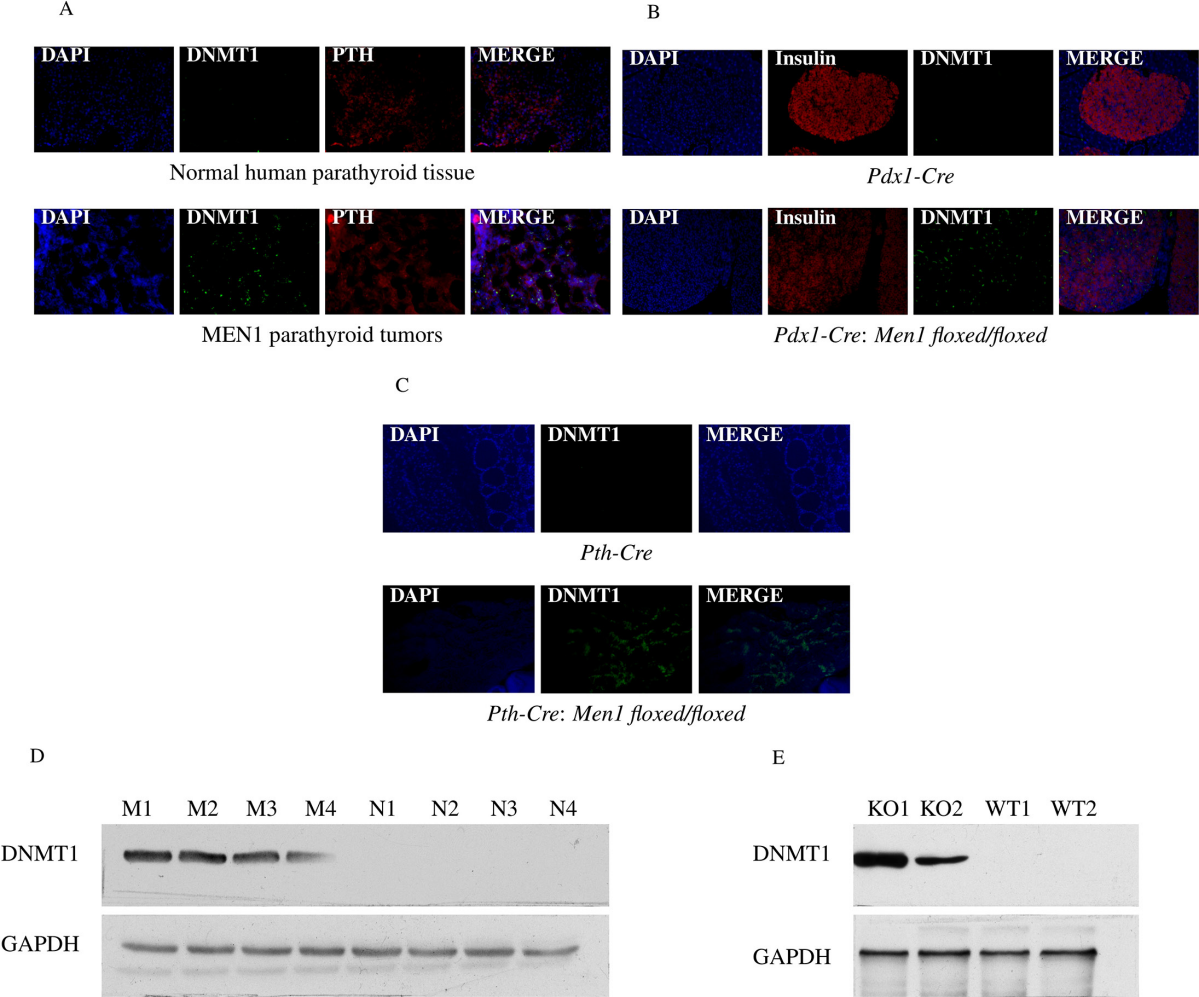
Increased expression of DNMT1 in endocrine tumor tissues from human MEN1 parathyroids and *Men1* KO mice DNMT1 expression was significantly increased in the parathyroid tumors from MEN1 patients (**A**), mouse pancreatic endocrine tumor tissues (**B**), and mouse parathyroid tumor tissues (**C**) from *Men1* KO mice compared to normal endocrine tissues from human and control mice by IF staining. DMNT1 was stained green by anti-DNMT1 antibody (Alexa Fluor 488); PTH and insulin were stained red by anti-PTH antibody or anti-insulin antibody, respectively (Alexa Fluor 647); and nuclei were stained blue with DAPI. Images were taken using a 20X objective. DNMT1 expression was determined in endocrine tumor tissues from the parathyroid in MEN1 patients (**D**) and pancreatic islets in *Men1* KO mice (**E**), compared to normal endocrine tissues from humans and control mice by western blot assay (***P < 0.001). (Normal/N: human normal parathyroids; MEN1/M: human MEN1 parathyroids; KO: *Men1* KO mice; WT: *Men1* WT mice).

Rbbp5 interacts with the promoter region of DNMT1 in human islet cells while in a complex with menin (*P* value < 0.0001) [7]. This finding has been validated in *Men1* null and wild type (WT) mouse embryonic fibroblast (MEF) cells by ChIP-PCR (Supplementary Figure 2). Based on this, we examined the expression of DNMT1 in endocrine tumor tissues from MEN1 patients and *Men1* KO mice. The loss of menin was confirmed in our *Men1* KO mouse models in parathyroid or pancreatic tissues by immunohistochemistry and western blot analysis as previously described [31–32]. DNMT1 mRNA expression was significantly increased in the endocrine tumors from MEN1 patients and *Men1* KO mice compared to normal by real-time RT-PCR (Figure 2). Immunofluorescence (IF) and western blot assays were used to identify increased DNMT1 protein levels in MEN1-parathyroid tumors (Figure 3A and 3D), mouse pancreatic endocrine tumors from *Men1* KO mice (Figure 3B and 3E), and mouse parathyroid tumors from a different *Men1* KO mouse model (Figure 3C). We measured DNMT1 mRNA expression in patients with sporadic parathyroid adenomas and carcinomas, and observed no significant difference in these tissues compared to normal human parathyroid tissues (Supplementary Figure 3). Interestingly, mRNA expression of DNMT1 in the human parathyroid adenoma with a *MEN1* mutation (described above) was increased compared to normal (Supplementary Figure 3). To determine if inactivation of menin increased the other three DNA methyltransferases (DNMT2, DNMT3a, and DNMT3b), we measured mRNA expression levels in the endocrine tumors from MEN1 patient samples and *Men1* KO mouse models, and demonstrated no significant increase in DNMT2, DNMT3a, and DNMT3b (Supplementary Figure 4). Taken together, these findings indicate that DNMT1 expression is significantly and specifically upregulated following the loss of menin function.

### Increased enzymatic activity of DNMT1 following loss of *MEN1* in tissues from patients and mice

**Figure 4:**
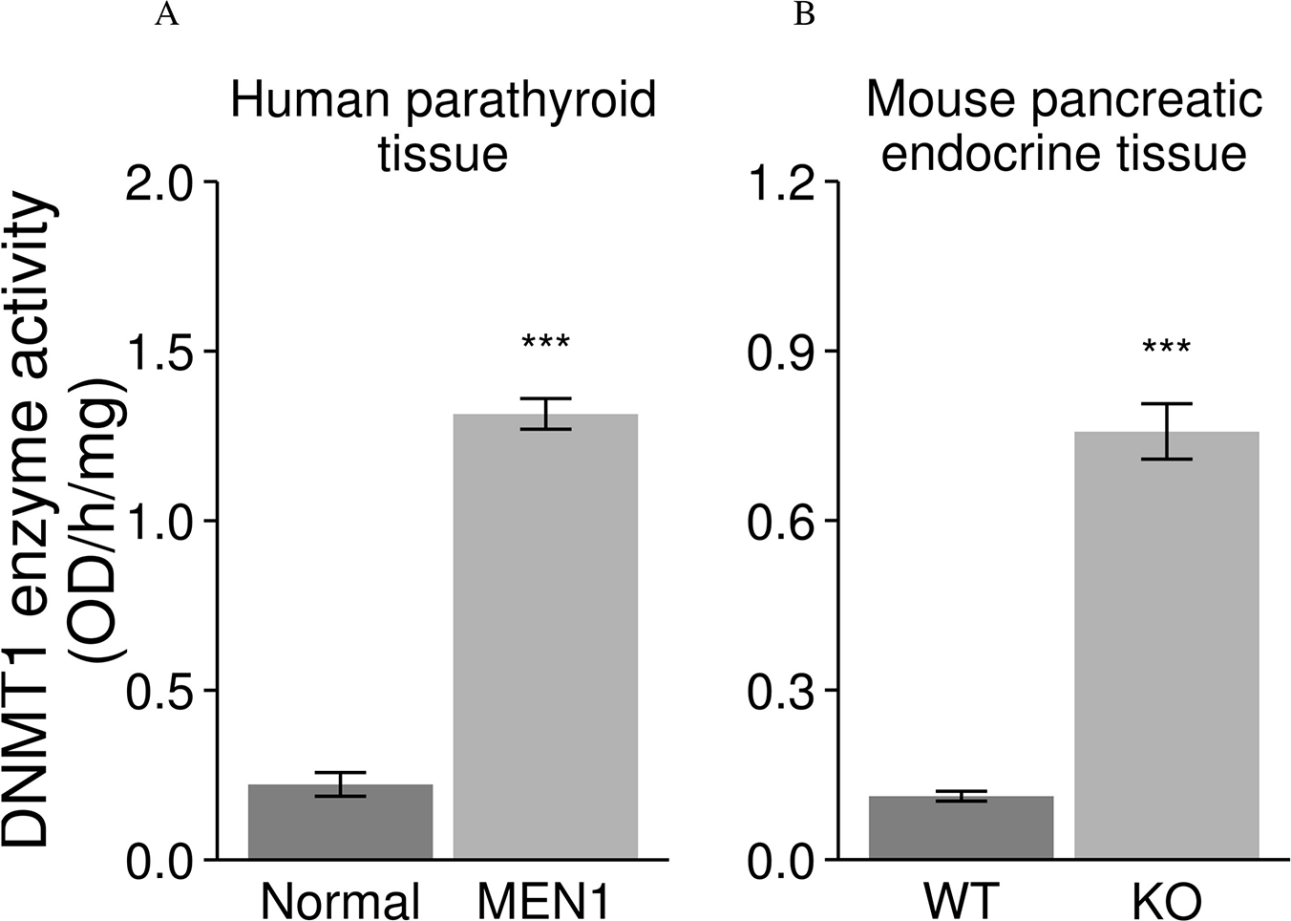
Increased enzymatic activity of DNMT1 in endocrine tumors from human MEN1 parathyroids and *Men1* KO mice DNMT1 enzymatic activity was significantly increased in the parathyroid tumor tissues from MEN1 patients (**A**) and mouse pancreatic endocrine tumors (**B**) from *Men1* KO mice compared to normal endocrine tissues from humans and control mice by a functional DNMT1 assay. (Normal: human normal parathyroids; MEN1: human MEN1 parathyroids; KO: *Men1* KO mice; WT: *Men1* WT mice). (***P < 0.001).

DNMT1 enzymatic activity was measured in endocrine tissues from MEN1 patient samples and *Men1* KO mice. We found that DNMT1 enzymatic activity was significantly increased in the endocrine tumor tissues from MEN1 patients and *Men1* KO mice as compared to normal endocrine tissues from unaffected individuals and *Men1* WT control mice (Figure 4).

### Inhibition of menin in *Men1* WT MEF cells leads to induction of DNMT1 mRNA expression and enzymatic activity

**Figure 5:**
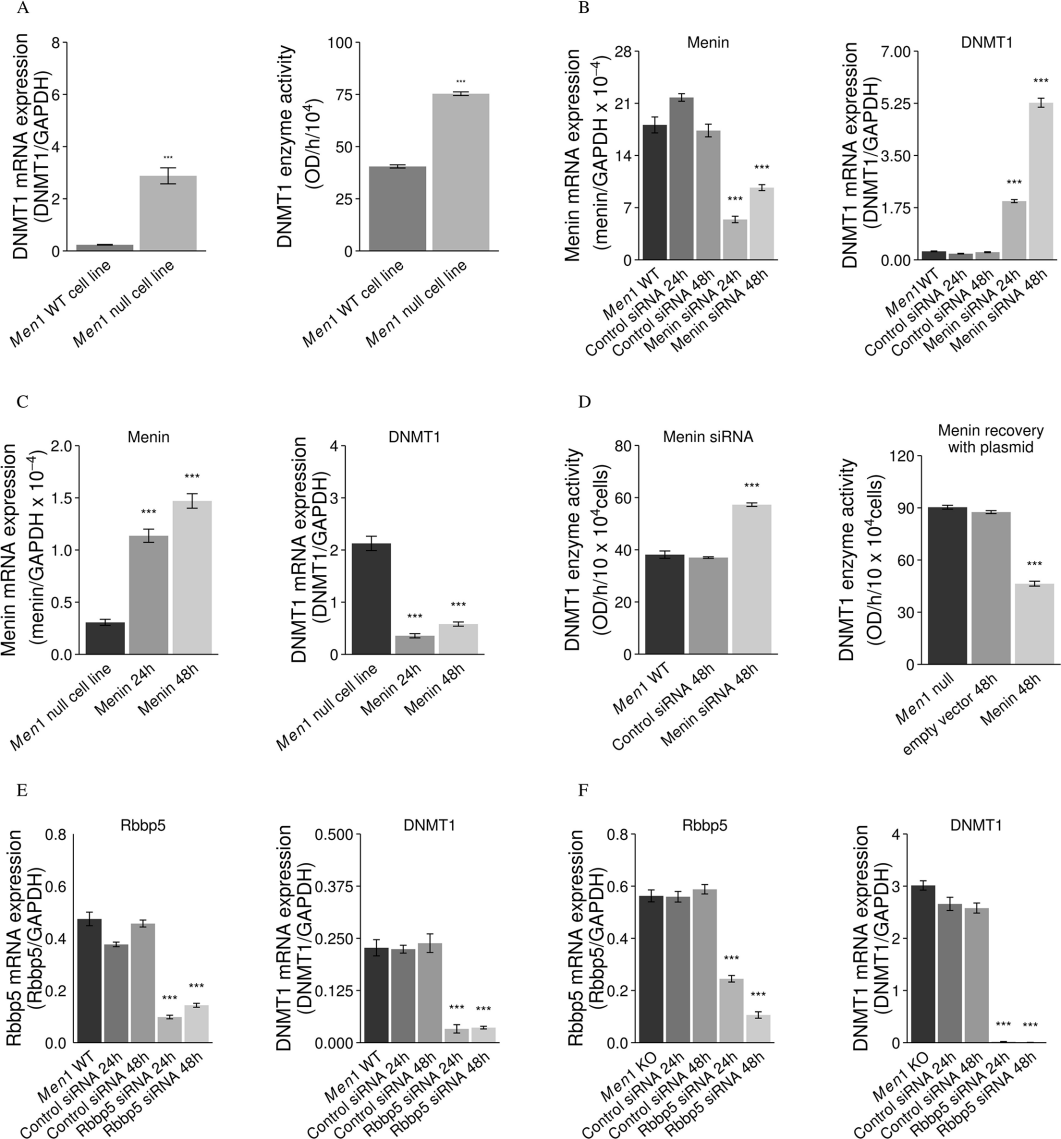
Menin suppresses DNMT1 expression and activity, but Rbbp5 enhances DNMT1 expression mRNA expression and activity of DNMT1 was significantly increased in a *Men1* null cell line as compared to a *Men1* WT cell line (**A**). The expression and activity of DNMT1 was up-regulated with knockdown of menin (siRNA) in a mouse *Men1* WT cell line (**B** and **D**) and the expression and activity of DNMT1 was down-regulated by inducing menin expression with a menin expression plasmid (**C** and **D**). However, knockdown of Rbbp5 with Rbbp5 siRNA reduced DNMT1 mRNA expression in both *Men1* WT (**E**) and *Men1* null mouse (**F**) cell lines. (***P < 0.001).

DNMT1 mRNA expression and activity were significantly increased in a *Men1* null cell line compared to a *Men1* WT cell line (Figure 5A). Silencing menin in the mouse *Men1* WT cell line using siRNA against *Men1* gene transcripts significantly upregulated DNMT1 (Figure 5B). The expression of DNMT1 in the mouse *Men1* null cell line was downregulated by menin overexpression via a menin expression plasmid (Figure 5C). DNMT1 enzymatic activity was increased by knockdown of menin with menin siRNA in the mouse *Men1* WT cell line, while, in contrast, DNMT1 activity was decreased by menin overexpression via a menin expression plasmid in the mouse *Men1* null cell line (Figure 5D). Transfection of scrambled control siRNA had no effect on DNMT1 expression or enzymatic activity.

### Inhibition of Rbbp5 in mouse *Men1* null and WT MEF cells leads to a reduction of DNMT1 mRNA expression

We induced menin expression via a menin expression plasmid in a mouse *Men1* null cell line; or we silenced menin and/or Rbbp5 with siRNA in mouse *Men1* null and WT cell lines. In the mouse *Men1* null and WT MEF cell lines, knockdown of Rbbp5 with Rbbp5 siRNA reduced DNMT1 mRNA expression (Figure 5E and 5F), suggesting inactivation of menin leads to increased activity of DNMT1 through Rbbp5. Scrambled control siRNA had no effect on DNMT1 expression.

### Rbbp5 interacts with the DNMT1 promoter resulting in increased DNMT1 expression in endocrine but not in exocrine tissues of pancreas

**Figure 6:**
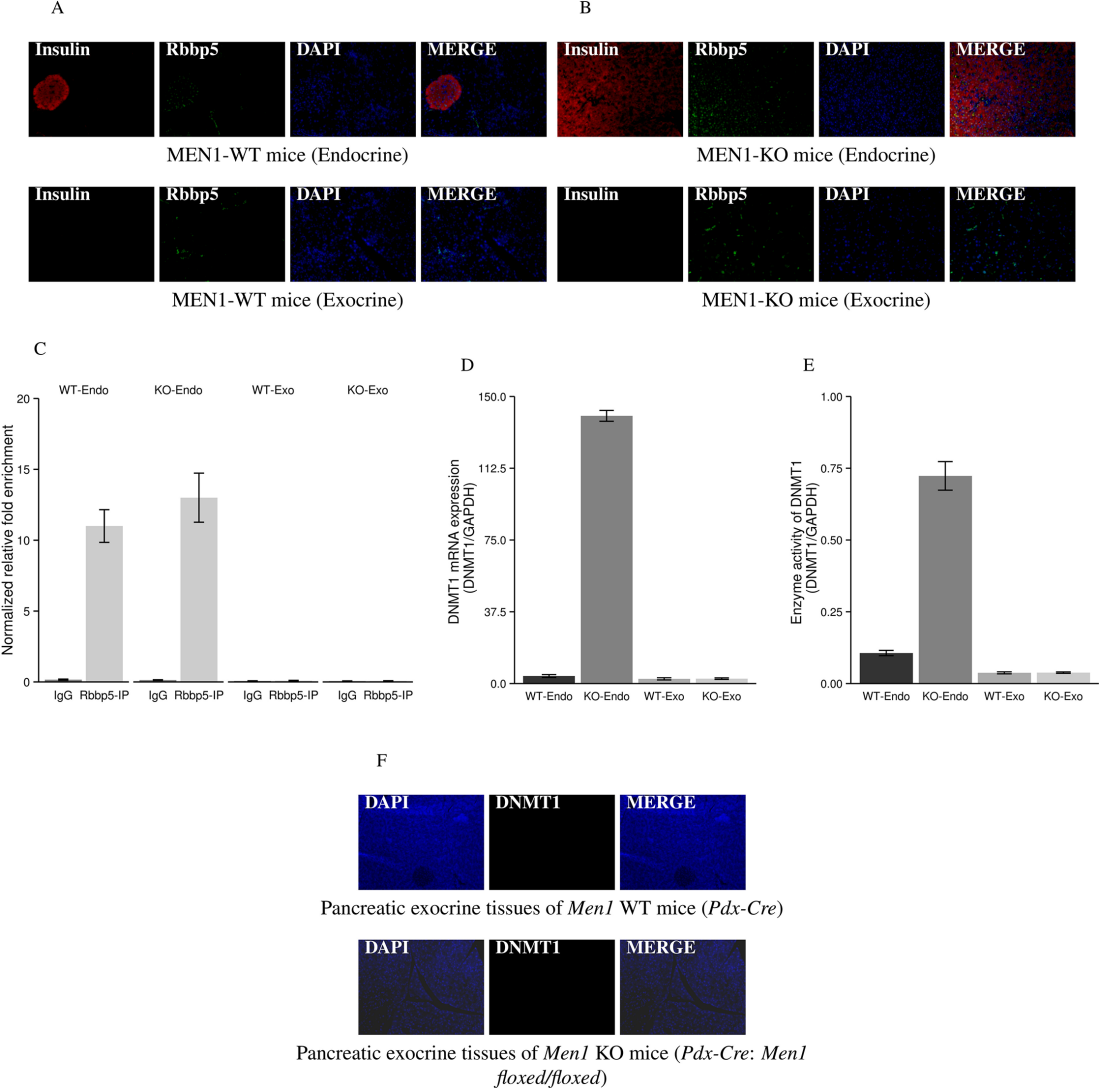
IF staining for Rbbp5 and DNMT1 expression and ChIP-PCR analysis of Rbbp5 binding to the DNMT1 promoter in pancreatic endocrine and exocrine tissues Rbbp5 protein was seen in both endocrine and exocrine tissues of the pancreas from *Men1* WT (**A**) and KO (**B**) mice by IF staining. Rbbp5 bound the DNMT1 promoter in the pancreatic endocrine tissue, but not in the pancreatic exocrine tissue, from *Men1* WT and KO mice (**C**). The Rbbp5-IP products were amplified using quantitative real-time RT-PCR with primers corresponding to the promoter regions of the DNMT1 gene. The relative amount of DNA in the ChIP product was calculated using the input for normalization. IgG was used as a control. The mRNA, protein expression, and enzymatic activity of DNMT1 were increased in the pancreatic endocrine tumor tissues from *Men1* KO mice compared to normal endocrine tissues from control mice (**D** and **E**). No increases were observed in pancreatic exocrine tissues from Men1 KO mice compared to normal exocrine tissues from control mice (**D**, **E**, and **F**).

Our mouse model shows *Men1* deletion in the whole pancreas induces tumorigenesis only in endocrine tissue and not in the exocrine tissue. To better understand the tissue-specific phenotype, we investigated whether Rbbp5 interacts with the promoter region of DNMT1 in both tissue types and examined DNMT1 expression in endocrine and exocrine pancreas of *Men1* WT and KO mice. There was no difference in the expression of Rbbp5 in endocrine and exocrine pancreatic tissues in *Men1* WT and KO mice (Figure 6A and 6B). However, Rbbp5 only bound the DNMT1 promoter in endocrine pancreatic tissue in both WT and *Men1* KO mice by ChIP-PCR assay (Figure 6C). The mRNA levels, protein expression, and enzymatic activity of DNMT1 were increased in the endocrine pancreas from the *Men1* KO mice when compared to normal endocrine tissue from WT mice (Figures 6D, 6E, 3B, and 3E). We found no differences between the exocrine pancreatic tissue from *Men1* KO mice and *Men1* WT mice with respect to DNMT1 expression or activity (Figure 6F).

### *Sox* family genes are hypermethylated at the promoter region, resulting in decreased mRNA and protein expression in human MEN1-parathyroid tumors

**Figure 7:**
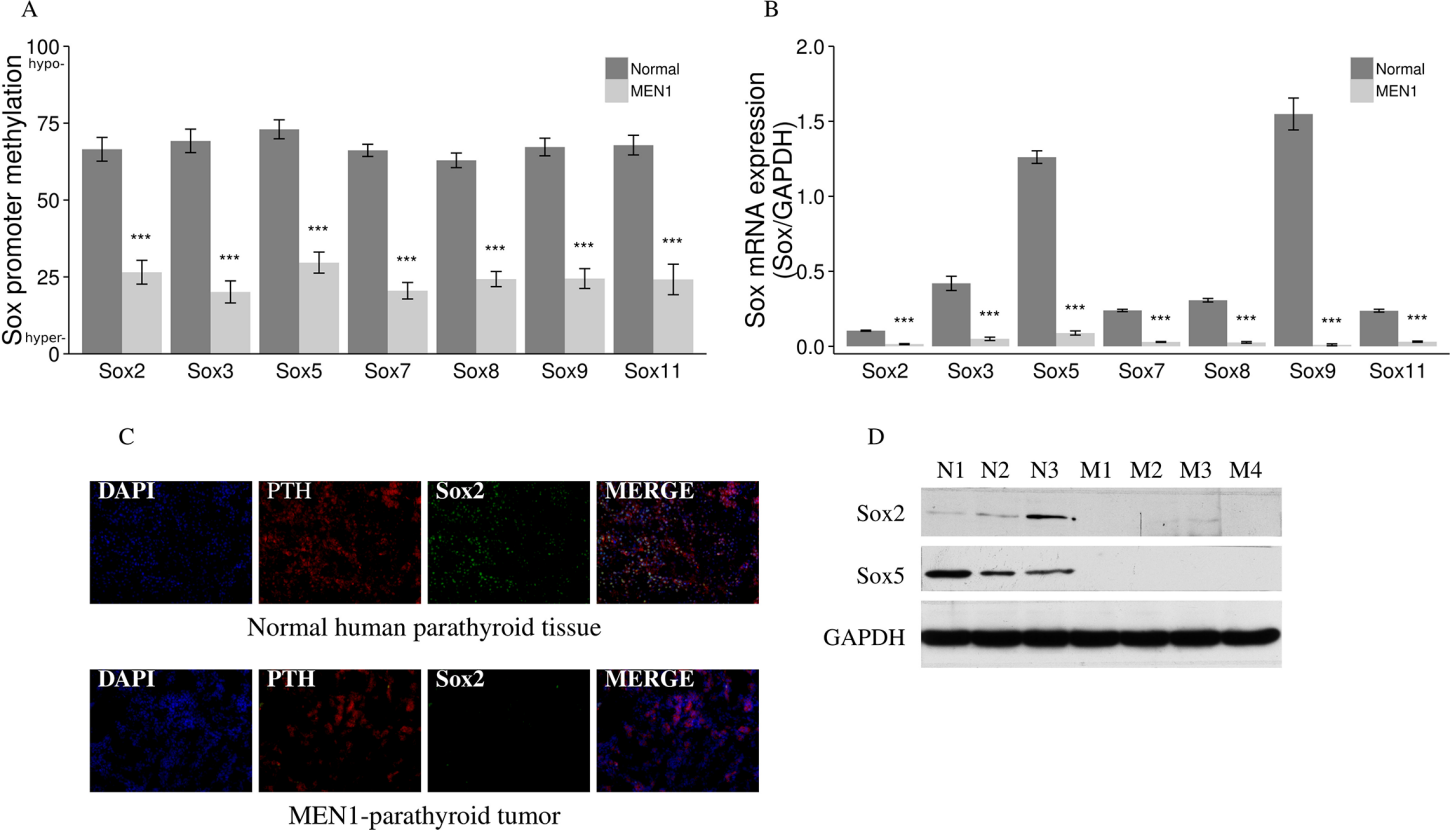
Aberrant methylation and expression of *Sox* genes in endocrine tumors from MEN1 patients Multiple *Sox* genes were hypermethylated with *P* < 0.0001 (*Sox* promoter methylation in the y axis depict the mean of the loci specific methylation. 0, fully methylated, to 100, fully unmethylated) (**A**) which correlates with a decreased mRNA expression of *Sox* genes (**B**) in all human MEN1-parathyroid tumors as compared to normal parathyroid tissues. Sox2 was also decreased when comparing MEN1-parathyroid tumors to normal control tissues (**C**) by IF staining. PTH was stained red by anti-PTH antibody (Alexa Fluor 647); Sox2 was stained green by anti-Sox2 antibody (Alexa Fluor 488); and nuclei were stained blue with DAPI. Images were taken with the 20X objective. Sox2 and Sox5 expression were decreased in parathyroid tumors from MEN1 patients compared to controls by western blot assay (**D**). (*P < 0.05, **P < 0.01, ***P < 0.001). (Normal/N: human normal parathyroids; MEN1/M: human MEN1 parathyroids).

We selected the top 2161 genes with the most significant methylation changes (methylation change of 20% or greater and *P* value < 0.05) and performed pathway analysis using bioinformatics and the Ingenuity Pathway Analysis (IPA) program. IPA determined that the hypermethylated genes in our datasets were significantly associated with the following canonical pathways: Eukaryotic Initiation Factor 2 (EIF2), Wnt/β-catenin, Oxidative Phosphorylation, and Granzyme A. Of the top four signaling pathways identified by IPA, only the Wnt/β-catenin pathway was uniquely relevant to tumorigenesis. Enrichment of the Wnt/β-catenin pathway could be explained by the high probability of Sox signaling involvement, based on the presence of seven *Sox* genes in our differentially methylated gene list. Sox family members were hypermethylated in all human MEN1-parathyroid tumors as compared to normal human parathyroid tissue (*P* value < 0.0001) (Figure 7A) and showed significant decrease in their mRNA expression (*P* value < 0.001) (Figure 7B). We further correlated the HELP-tagging and mRNA expression data on a gene expression heatmap, showing a Pearson's correlation coefficient (R) of 0.88–0.93 (Supplementary Figure 5). The decreased expression of some Sox family members was further demonstrated through IF staining of normal and MEN1 tissue samples, where the Sox2 protein decreased compared to controls (Figure 7C). The decreases in Sox2 and Sox5 proteins in MEN1 tissues were also confirmed by western blot assay (Figure 7D).

### Decreased mRNA and protein expression of *Sox* genes is seen in endocrine tumors of the pancreas and parathyroids from two *Men1* KO mouse models

**Figure 8:**
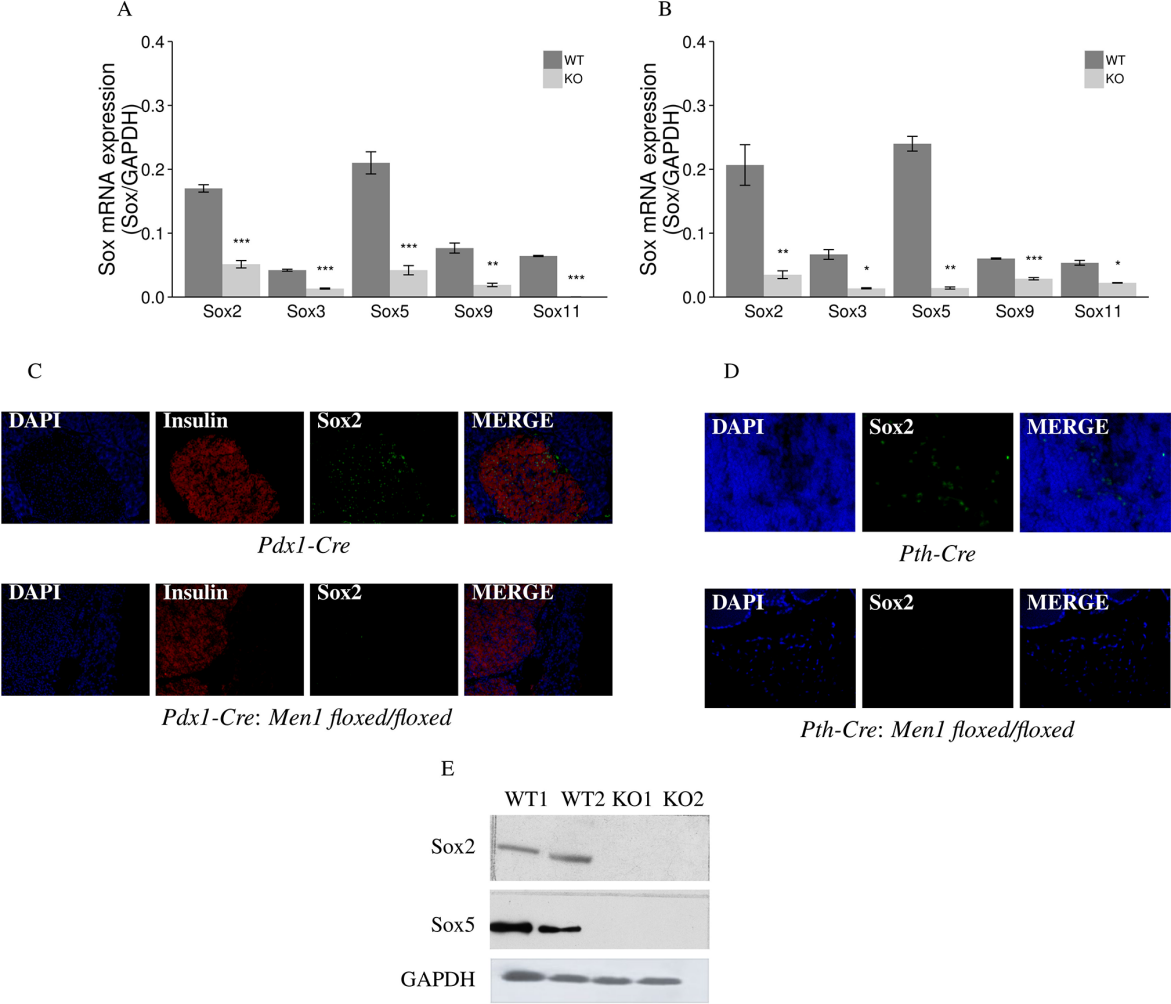
Aberrant methylation and expression of Sox genes in endocrine tumors from *Men1* KO mice *Sox* mRNA expression was decreased in endocrine tumors of the pancreas (**A**) and parathyroids (**B**) from *Men1* KO mice compared to normal endocrine tissues from control mice by real-time RT-PCR. The expression level of Sox2 protein was also decreased when comparing endocrine tumors of the pancreas (**C**) and parathyroids (**D**) from *Men1* KO mice compared to normal endocrine tissues from control mice by IF staining. Insulin was stained red by anti-insulin antibody (Alexa Fluor 647); Sox2 was stained green by anti-Sox2 antibody (Alexa Fluor 488); and nuclei were stained blue with DAPI. Images were taken with the 20X objective. Sox2 and Sox5 expression were decreased in pancreatic endocrine tumor tissues from Men1 KO mice compared to normal pancreatic endocrine tissues from *Men1* WT mice by western blot assay (**E**). (*P < 0.05, **P < 0.01, ***P < 0.001). (WT: *Men1* WT mice; KO: *Men1* KO mice).

Five *Sox* genes (*Sox2, Sox3, Sox5, Sox9,* and *Sox11*) showed significantly decreased mRNA expression (from 4 to 12 fold) in the endocrine tumor tissues of the pancreas and the parathyroids from *Pdx1-Cre:Men1 floxed/floxed* and *Pth-Cre:Men1 floxed/floxed* KO mice compared to normal endocrine tissues from the pancreas and parathyroids from control *Pdx1-Cre* and *Pth-Cre* mice, respectively (Figure 8A and 8B). Decreased protein expression of Sox2 was also confirmed in endocrine tumors of the pancreas and parathyroids from the *Men1* KO mice by IF and western blot assays (Figure 8C, 8D, and 8E).

### Downregulation of the *Sox* gene family results in increased β-catenin expression in endocrine tumors from MEN1 patients and *Men1* KO mice

**Figure 9:**
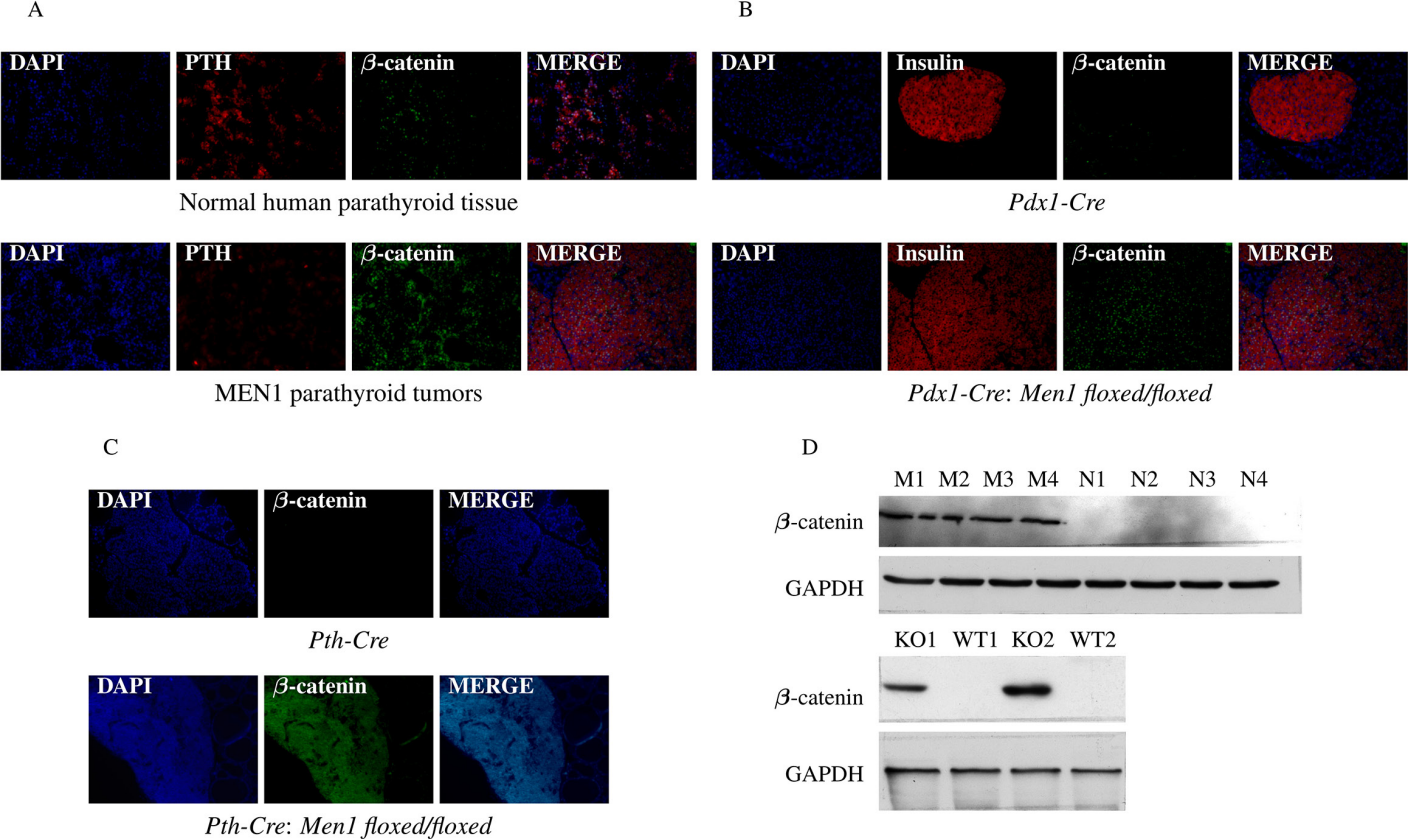
Hypermethylation of *Sox* genes results in increased β-catenin expression The presence of β-catenin was significantly increased in parathyroid tissues from MEN1 patients (**A**), mouse pancreatic endocrine tumors (**B**), and mouse parathyroid tumors (**C**) from *Men1* KO mice compared to normal endocrine tissues from humans and control mice by IF staining. PTH and insulin were stained red by anti-PTH antibody and anti-insulin antibody, respectively (Alexa Fluor 647); β-catenin was stained green by anti-β-catenin antibody (Alexa Fluor 488); and nuclei were stained blue with DAPI. Images were taken with the 20X objective. Increased β-catenin expression in parathyroid tumors from MEN1 patients and pancreatic endocrine tumors from *Men1* KO mice was further confirmed by western blot assay (**D**).

*Sox* genes exert an inhibitory effect on the accumulation of β-catenin, the effector protein of the Wnt/β-catenin signaling pathway directly implicated in tumorigenesis [33]. IF and western blot assays in our human MEN1 samples showed increased expression of β-catenin (Figure 9A and 9D). Increased expression of β-catenin in the pancreas and parathyroids of *Men1* KO mice was seen in both the *Pdx1-Cre:Men1 floxed/floxed* (Figure 9B and 9D) and *Pth-Cre:Men1 floxed/floxed* (Figure 9C) models. DNA hypermethylation of *Sox* gene promoters caused by the loss of menin resulted in the downregulation of the Sox signaling pathway, leading to enhanced Wnt/β-catenin pathway signaling.

### Specific inhibition of *Sox* genes is achieved with *Sox2* and *Sox5* siRNA leading to increased β-catenin mRNA expression

**Figure 10:**
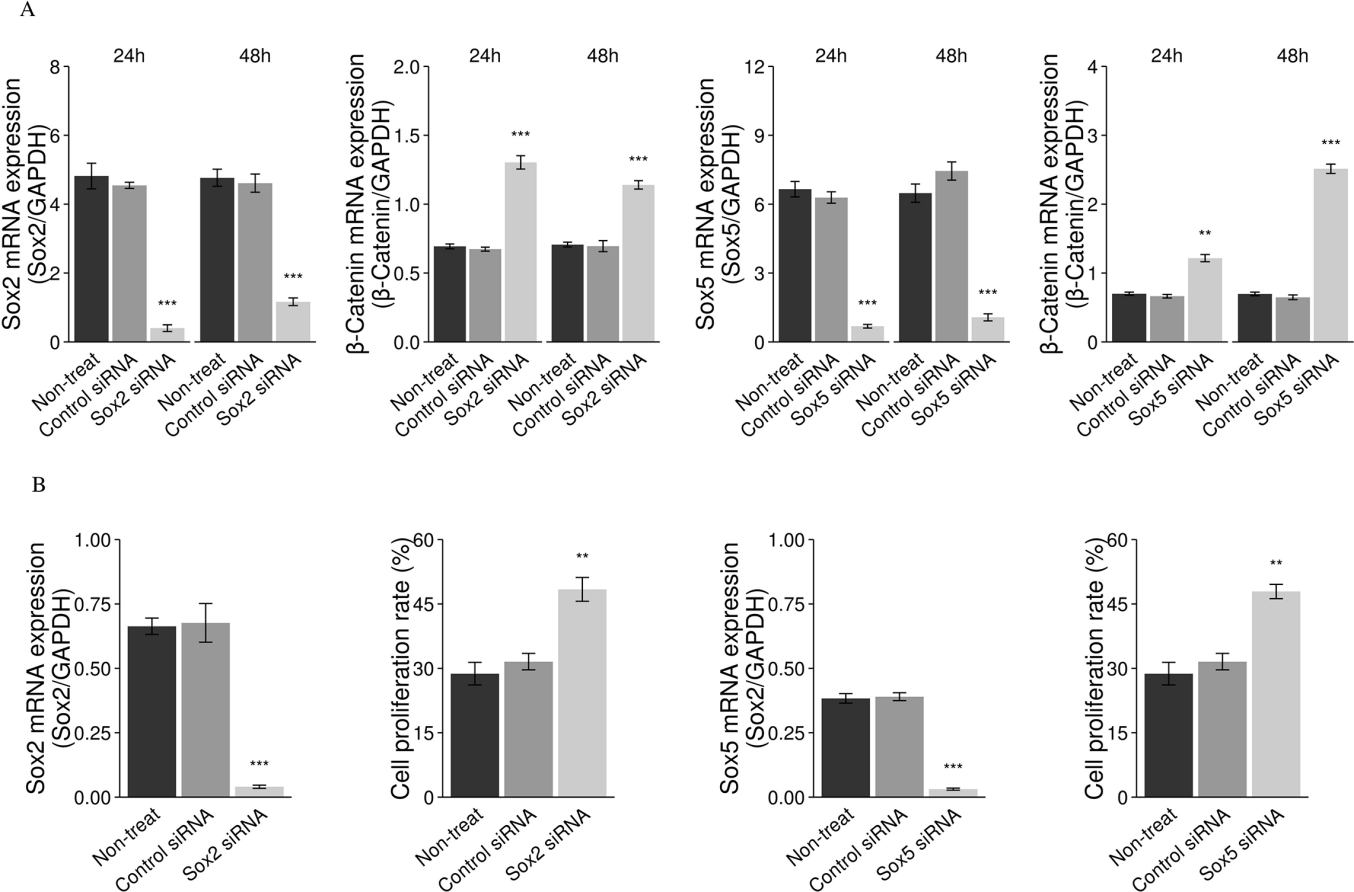
Downregulation of *Sox2* genes with siRNA results in increased cell proliferation The mRNA expression of β-catenin was increased following knockdown of the *Sox2* and *Sox5* genes with siRNA in a mouse *Men1* WT cell line, using a real-time RT-PCR assay (**A**). Cell proliferation was increased following knockdown of *Sox2* and *Sox5* genes, with *Sox2* siRNA and *Sox5* siRNA in a human pancreatic tumor cell line (BON-1), using IF staining for the Ki-67 marker (**B**). (*P < 0.05, **P < 0.01, ***P < 0.001).

We used siRNAs to knockdown *Sox2* and *Sox5* expression in a *Men1* WT MEF cell line. *Sox2* and *Sox5* siRNAs were tested for knockdown efficiency and both siRNAs were shown to decrease the mRNA levels of their targets (Figure 10A). The mRNA level of β-catenin determined by real-time RT-PCR, was significantly increased following *Sox2* and *Sox5* knockdown (Figure 10A). No effect was seen with scrambled control siRNAs. Cell proliferation was increased following knockdown of *Sox2* and *Sox5* genes with siRNA in a human pancreatic endocrine cell line, BON-1, as determined by IF staining for the Ki-67 marker (Figure 10B).

### Increased *Sox* mRNA expression and downregulated β-catenin in the *Men1* null and WT MEF cells following treatment with the demethylating agent 5-aza-2′-deoxycytidine

**Figure 11:**
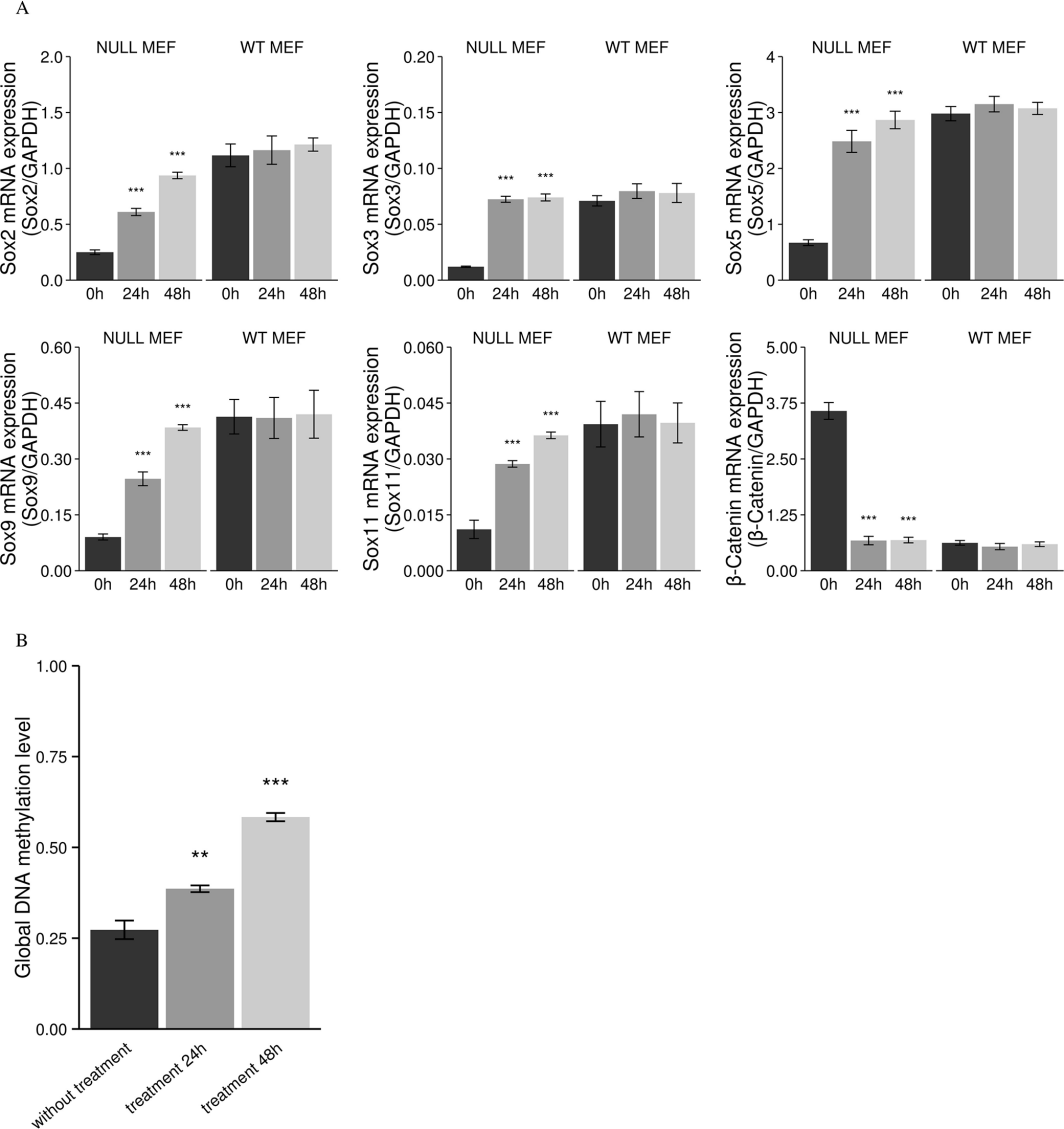
Increased mRNA expression of *Sox* genes, subsequent decreased mRNA expression of β-catenin, and decreased global DNA methylation after treatment with the demethylating agent 5-aza-2′-deoxycytidine in *Men1* null MEF cells In *Men1* null MEF cells, relative mRNA of *Sox2*, *Sox3*, *Sox5*, *Sox9*, *Sox11*, and β-catenin was measured after treatment with 1 μM 5-aza-2′-deoxycytidine for 24 h and 48 h, the asterisks indicate that the mRNA expression of *Sox* genes are significantly increased and the mRNA expression of β-catenin is significantly decreased as compared to controls. However, mRNA expression of *Sox* and β-catenin were not significantly changed in *Men1* WT MEF cells after treatment (**A**). The global DNA methylation level is significantly decreased in *Men1* null MEF cells by selective degradation DNMT1 with the demethylating agent treatment (0, fully methylated; 1, fully unmethylated) (**B**). (*P < 0.05, **P < 0.01, ***P < 0.001).

In order to further confirm that methylation of *Sox* genes results in decreased *Sox* mRNA expression, mouse *Men1* null and WT cells were treated with 1.0 μM of the demethylating agent 5-aza-2′-deoxycytidine for 24 hours. All cells were harvested at 24 or 48 hours. *Men1* null cells showed significantly increased *Sox* mRNA expression levels after treatment and achieved similar expression to the *Men1* WT cells (Figure 11A). Furthermore, mRNA expression of β-catenin was significantly downregulated to a similar expression level of *Men1* WT cells in the treated *Men1* null cells (Figure 11A). The mRNA expression of *Sox* and β-catenin was not significantly changed in *Men1* WT cells after treatment with 5-aza-2′-deoxycytidine (Figure 11A).

### Decreased levels of global DNA hypermethylation in the *Men1* null MEF cells following treatment with the demethylating agent 5-aza-2′-deoxycytidine

Selective degradation of DNMT1 was induced by 5-aza-2′-deoxycytidine in the *Men1* null cell line. We demonstrated, by LUMA, that levels of global DNA methylation were significantly decreased in the *Men1* null cells following 5-aza-2′-deoxycytidine treatment compared to the *Men1* null cells without 5-aza-2′-deoxycytidine treatment (Figure 11B).

## DISCUSSION

Studies have shown the loss of menin increases DNA hypermethylation at the promoter region of several genes [21–22]. Human cancer genomes are characterized by widespread aberrations in DNA methylation patterns [34–37]. Therefore, the characterization of global DNA methylation patterns in the genomes of tissues with loss of the *MEN1* tumor suppressor gene appeared an attractive area of investigation.

In the present study, we used global DNA methylation profiling to identify the top cellular pathways affected by the loss of menin, using human parathyroid tissue as a starting point. We demonstrated a genome-wide increase in DNA methylation in MEN1-parathyroid tumors. Hypermethylation of the promoter region in particular genes has been analyzed in pancreatic and parathyroid tumors [22, 38–39], and in an unbiased quantification of DNA methylation [26]. Promoter hypermethylation can silence tumor suppressor genes, contributing to the cancer state. Polycomb Group (PcG) complexes have been shown to play an import role in epigenetic regulation [40], and we have observed that Polycomb genes in MEN1 tumors are differentially methylated compared to normal. Further studies on the Polycomb genes involvement in DNA methylation in MEN1 tumors are warranted.

Global hypermethylation of the genome is typically mediated by DNMTs [41–42]. Four DNMTs have been identified: DNMT1, DNMT2, DNMT3a, and DNMT3b [43]. The over-expression of DNMT1 has been shown to increase global DNA methylation [44–45]. We demonstrated that the expression and enzymatic activity of DNMT1 was significantly increased in tumor tissues from MEN1 patients, *Men1* KO mice, and *Men1* null cell lines. We did not observe any significant changes in the expression of *DNMT2*, *DNMT3a*, or *DNMT3b* genes (Supplementary Figure 4). These results indicate inactivation of menin may specifically impact DNMT1, but not DNMT2, DNMT3a, or DNMT3b.

Xu et al. reported that menin represses Pax2 expression through up-regulation of Wilms tumor suppressor protein (WT1) in MEF cells. WT1 recruits PcG proteins to the Pax2 promoter locus inhibiting Pax2 expression by increasing H3K27me3 [46]. WT1 can also bind DNMT1 and recruit it to the Pax2 promoter resulting in hypermethylation of CpGs and decreased expression. In addition, several studies have demonstrated that the expression levels of WT1 were reduced in parathyroid tumors [26, 47]. We have demonstrated that the expression level of WT1 is significantly decreased in MEN1-parathyroid tumors compared to normal parathyroid tissues (Supplementary Figure 6). Xu's report showed that the protein level of DNMT1 was not regulated by menin in MEF cells, but we found the level of DNMT1 in endocrine tumors from MEN1 patients and *Men1* KO mice was increased compared to normal. We believe this discrepancy might be related to tissue specificity. Further studies of menin and WT1 in specific tissues, especially endocrine tumors, are warranted.

In a previously described ChIP-chip assay [7], and confirmed in our ChIP-PCR experiment (Supplementary Figure 2), Rbbp5 was shown to bind menin and the DNMT1 promoter. We found that increasing menin or knocking down Rbbp5 in a mouse *Men1* null cell line led to the inhibition of DNMT1 expression and enzymatic activity. Furthermore, knockdown of menin in a mouse *Men1* WT cell line led to increased expression and enzymatic activity of DNMT1. These results suggest that Rbbp5 is an important co-activator of DNMT1, and menin is perhaps a co-repressor of Rbbp5 and DNMT1.

**Figure 12:**
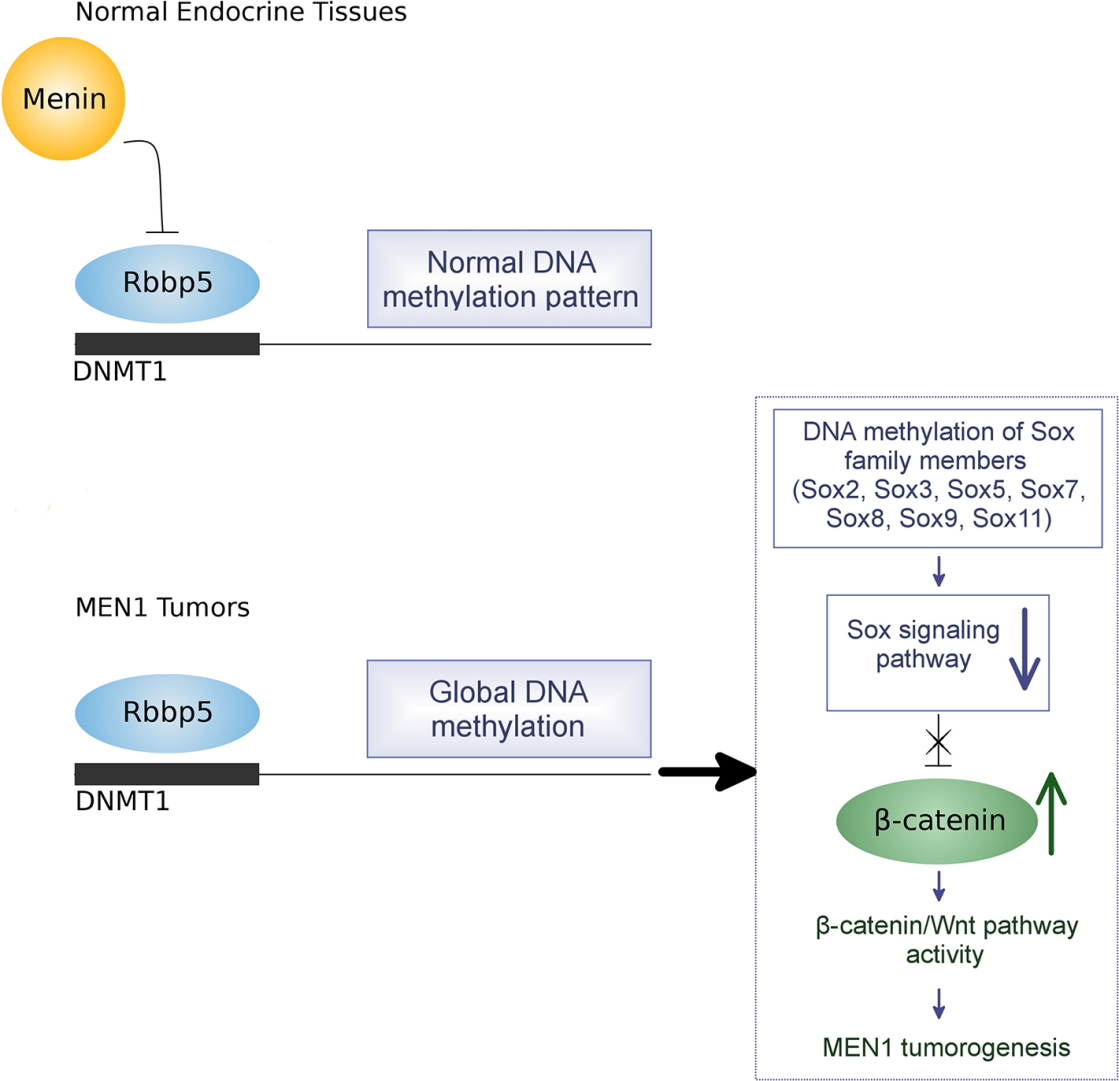
Proposed model of epigenetic regulation of MEN1 tumorigenesis by DNA hypermethylation

A bioinformatics approach was used to identify the most highly dysregulated pathways relevant to tumorgenesis and resulting from methylation changes. The *Sox* gene family in particular was found to have significant hypermethylation in MEN1-parathyroid tumors compared to normal parathyroid tissue. *Sox* genes are highly conserved and suppress β-catenin expression thereby inhibiting the Wnt/β-catenin signaling pathway [33]. Several studies have linked decreased expression of the *Sox* family genes with tumorigenesis [48–50]. For example, the decreased expression of *Sox7* is correlated with poor prognosis in lung cancer patients [48]. The *Sox2* gene is frequently involved in DNA hypermethylation and downregulation of *Sox2* in colorectal and gastric cancers results in increased cell proliferation [49–50]. However, a study reported that downregulation of *Sox2* inhibits cell proliferation in small cell lung cancer [51]. The relationship of the *Sox* gene family to the Wnt/β-catenin pathway was specifically shown when *Sox17* expression was inhibited by hypermethylation of the promoter region, resulting in activation of Wnt signaling in human thyroid cancer [52]. *Sox7* was reportedly downregulated in endometrial cancer showing increased β-catenin, CyclinD1, and fibroblast growth factor 9 (FGF9) [53]. We have examined the methylation status of the secreted Wnt antagonists (sFRP, DKKs and WIF1) and observed no methylation changes in MEN1-parathyroid tumors compared to normal parathyroid tissues. In the present study, we have observed members of the *Sox* family are downregulated by promoter methylation following the loss of menin. This downregulation is accompanied by an increase in both β-catenin mRNA and protein. This was seen in both human MEN1 tumor tissues and in two different *Men1* KO mouse models. To confirm DNMT1 regulation of *Sox* genes by hypermethylation, we treated a mouse *Men1* null cell line with the demethylating agent 5-aza-2′-deoxycytidine. This induced re-expression of the *Sox* gene family and downregulation of β-catenin. Furthermore, downregulating *Sox2* and *Sox5* genes with siRNA treatment resulted in increased cell proliferation of pancreatic endocrine cells. We have proposed a model of how MEN1 loss results in tumorigenesis partly through epigenetic modification and perturbation of signaling pathways (Figure 12). This model observes that menin, controls the expression of DNMT1 through its binding partner Rbbp5. Loss of menin results in unopposed Rbbp5 activation of DNMT1, leading to increased global DNA methylation, predominately in the promoter regions. The resulting decrease in the expression of *Sox* family genes, which are especially hypermethylated, leads to increased Δ-catenin transcription and degradation in Wnt/Δ-catenin signaling. This results in increased cell proliferation and tumorigenesis in endocrine tissue of the pancreas by increasing β-catenin/TCF activity and the expression of oncogenic Wnt-target genes.

The tissue-specific tumorgenesis of MEN1 remains poorly understood. To test if the binding of Rbbp5 to the promoter of DNMT1 was tissue-specific we performed CHIP-PCR analysis in both endocrine and exocrine tissues of the pancreas. We determined that Rbbp5 only binds the DNMT1 promoter in endocrine tissues and not in the exocrine tissues from *Men1* WT and KO mice. These results suggest several possible mechanisms for the tissue specific effects seen following the loss of menin. Pancreatic endocrine and exocrine tissues may possess different co-factors, different binding sequences in the DNMT1 promoter, or may be subject to different histone methylation patterns. We intend to further investigate these possible molecular mechanisms in an attempt to better understand the tissue-specific tumorgenesis seen in MEN1 syndrome. We hypothesize that the interplay between Rbbp5 and the DNMT1 promoter may, at least in part, explain this phenomenon.

Taken as a whole, these observations provide a possible model for MEN1-related tumor development (Figure 12). This study provides the first evidence that global, genome-wide DNA hypermethyaltion results from the loss of menin. As such, it provides important insights into the possible role of epigenetic mechanisms in the pathogenesis of MEN1 tumor development.

## MATERIALS AND METHODS

### Human tissue specimens

Thirty-eight human parathyroid specimens were obtained: 13 sporadic (non-MEN1) parathyroid adenomas, 12 MEN1-parathyroid tumors, 4 parathyroid carcinomas, and 9 normal parathyroids. All tissues were collected from individuals operated on at the National Cancer Institute (NCI) or the Albert Einstein College of Medicine (Einstein). All tissues were stored at −80°C and carefully evaluated by an experienced pathologist. All patients signed consent for a prospective clinical trial to obtain these tissues, and this study was approved by the Institutional Review Board (IRB) at both the NCI and Einstein.

### *Men1* KO Mice

We utilized the Cre-lox system, driven by the tissue-restricted promoters *Pdx1* and *Pth*, to inactivate *Men1* in the pancreas and parathyroids of mice. Mice with homozygous loss of *Men1* develop pancreatic endocrine and parathyroid tumors in *Pdx1*- or *Pth-Cre; Men1 floxed/floxed*, respectively [31–32]. Tissues from these mice were used to confirm and assess the findings from the MEN1 clinical samples and to further explore the molecular mechanisms of global epigenetic changes following the inactivation of menin.

### MEF cell line, human pancreatic endocrine tumor cell line, plasmid, and siRNA

*Men1* null and WT MEF cell lines were used to investigate the findings from MEN1 clinical samples. The plasmid used to transfect the *Men1* null MEF cell line to induce menin expression was provided by Dr. Sunita K. Agarwal, of the National Institute of Diabetes and Digestive and Kidney Diseases (NIDDK). The human endocrine tumor cell line BON-1 was obtained from Dr. Lopez (Uppsala University, Sweden), and used to investigate cell proliferation by inhibiting Sox2 and Sox5 expression with siRNA treatment in an *in vitro* cell proliferation assay. Various specific siRNAs that target menin, Rbbp5, Sox2, and Sox5 and controls were purchased from Santa Cruz biotechnology (Santa Cruz, CA) and were transfected into cell lines to knockdown gene expression.

### DNA extraction for HELP-tagging analysis

Genomic DNA was extracted from parathyroid specimens using a protocol developed by the Einstein Epigenomic Facility. Approximately 0.1 g of frozen tissue was crushed under liquid nitrogen using a mortar and pestle. The crushed tissues were incubated in 10 ml DNA extraction buffer (10 mM Tris-HCl, 0.1 M EDTA, 0.5% SDS, and 10 μl of 20 mg/mL RNaseA) at 37°C for one hour, then incubated with 50 μl Proteinase K at 50°C overnight. An equal volume of saturated phenol was added to the DNA extraction buffer and mixed slowly at room temperature for 15 minutes then centrifuged at 3000 rpm for 10 minutes at room temperature. The supernatant was carefully removed and transferred to a fresh 50 ml falcon tube. This was repeated twice more with saturated phenol and then three times more with chloroform. The sample was then pipetted into a prepared dialysis bag and the open end was clipped shut. The dialysis bag was placed on a pile of polyethylene glycol (PEG) crystals covered with saran wrap and covered with additional PEG crystals, then left to sit for an hour or until ∼0.5–1 ml of fluid remained. The DNA was collected from the dialysis bag and stored at 4°C for further HELP-tagging analysis.

### HELP-tagging for global DNA methylation analysis

First, in order to perform the HELP-tagging assay, DNA samples from patients were digested in parallel with one methylation-sensitive (HpaII) and one methylation-insensitive (MspI) isoschizomers that recognize CCGG sites. Secondly, the digested DNA samples were ligated to a specially designed adaptor with an EcoP151 site, a 27 bp flanking genomic fragment was liberated by digestion with an EcoP151 restriction enzyme, and another adapter was added to the other end for library generation by PCR. The generated libraries were sequenced by the Illumina Genome GAIIx/HiSeq 2000 massively parallel sequencer following the manufacturer's instructions. Thirdly, the HpaII signal was normalized with that of the massively parallel sequenced MspI profiles, as performed previously [29–30]. The methylation at individual CCGG loci was quantified by a comparison of the normalized counts of the 2 isoschizomers. Methylation scores were defined in a continuous variable model and are depicted from a range of 0, fully methylated loci, to 100, fully unmethylated loci [29–30]. To define DNA methylation differences, we observed that approximately 80% of the annotated HpaII sites in the human genome (hg18) were represented by at least one read, for a total of over 1.8 million loci throughout the genome. The mean number of reads per locus was 1.32. All methylation data generated in this study has been deposited in the NCBI repository (accession number GSE64412).

### Quantitative validation for single locus by Bisulfite-Massarray and Pyrosequencing analysis

We used Bisulfite-MassArray and Pyrosequencing to test the quantitative methylation changes of global cytosine loci against those detected by HELP-tagging. Bisulfite conversion was performed with an EZ DNA Methylation kit (Zymo Research, Orange, CA, USA). Bisulfite primers for Bisulfate-MassArray and Pyrosequencing were designed using MethPrimer software [54]. The primer sequences are provided in Supplementary Table 6.

### Quantitative real-time RT-PCR

Total RNA was extracted with the RNeasy total RNA kit (Qiagen, Valencia, CA, USA). mRNA expression was detected by quantitative real-time RT-PCR. PCR products were measured by fluorescent signal intensity after standardization with glyceraldehyde-3-phosphate dehydrogenase (GAPDH) for internal controls. The primers are listed in Supplementary Table 7.

### IF staining

For the detection of protein expression of DNMT1, Sox2, and β-catenin in human and animal tissues, 5 μm-thick paraffin or optimum cutting temperature (OCT) sections were stained by dual IF. The sections were incubated with primary antibodies (1:100 dilution of rabbit anti-DNMT1 antibody [Abcam, Cambridge, MA], 1:50 dilution of rabbit anti-Sox2 antibody [Santa Cruz Biotech, Santa Cruz, CA], 1:100 dilution of rabbit anti-β-catenin antibody [Santa Cruz Biotech, Santa Cruz, CA], 1:200 dilution of mouse anti-PTH antibody [Abcam, Cambridge, CA], and 1:500 dilution of pig anti-insulin antibody [Dako, Carpinteria. CA]) overnight at 4°C. Slides were then incubated with secondary antibodies (1:200 dilutions each of anti-mouse Alexa Fluor 647, anti-pig Alexa Fluor 647, and anti-rabbit Alexa Fluor 488; Invitrogen, Grand Island, NY, USA) for 45 minutes in the dark. The slides were then mounted in Vectashield mounting medium with 4′, 6-diamidino-2-phenylindole (DAPI; Vector Laboratories, Burlingame, CA, USA). Images were taken using a fluorescence microscope with a camera.

### Western blot assay

Total protein was extracted from human (parathyroid tissues) and animal tissues (pancreatic islet tissues), and mouse cell lines using Radioimmunoprecipitation assay (RIPA) lysis buffer (Pierce, Rockford, IL). The extracted proteins were quantified and equal amounts of protein were separated by SDS-PAGE and blotted. Because of the limited amount of mouse parathyroid tissues, western blots with mouse parathyroid tissues were not possible. The primary antibodies used were: 1:200 dilution of mouse anti-GAPDH antibody (Santa Cruz biotechnology, Santa Cruz, CA), 1:300 dilution of rabbit anti-DNMT1 antibody (Abcam, Cambridge, M), 1:400 dilution of rabbit anti-Rbbp5 antibody (Abcam, Cambridge, MA), 1:300 dilution of goat anti-menin antibody (Santa Cruz Biotech, Santa Cruz, CA), 1:200 dilution of goat-anti Sox2 antibody (Santa Cruz Biotech, Santa Cruz, CA), and 1:300 dilution of rabbit anti-β-catenin antibody (Santa Cruz Biotech, Santa Cruz, CA), respectively. The secondary antibodies used were: 1:5000∼10000 dilution of anti-mouse IgG-HRP, anti-rabbit IgG-HRP, and anti-goat IgG-HRP, respectively. The small differences in loading were corrected by comparison to the loading control (GAPDH).

### ChIP-PCR assay

We used ChIP-PCR to validate Rbbp5-DNMT1 promoter interaction in pancreatic endocrine and exocrine tissues from *Men1* WT and *Men1* KO mice, and *Men1* WT and *Men1* null MEF cells with a Magna ChIP kit (Millipore, CA) according to the manufacturer's protocol. Briefly, pancreatic tissues and cell lines were collected and cross-linked with 1% formaldehyde for 10 minutes at room temperature. Cross-linking was stopped by adding glycine for 5 minutes then cells were washed three times with ice-cold PBS/proteinase inhibitor. The nuclear extract was prepared, and chromatin was sonicated to a size of 500–1000 bp. The Rbbp5 ChIP assay was performed by incubating 100 μg of MEF chromatin with 5 μl (1 μg/μl) of Rbbp5 antibody (Bethyl, TX) overnight at 4°C. Rabbit IgG was used as a negative control. Protein G magnetic beads were used to collect the immunoprecipitates. The Rbbp5-ChIP DNA was assayed by PCR using primers corresponding to the promoter regions of DNMT1 gene (Supplementary Table 8).

### DNMT1 enzymatic activity assay

Nuclear complexes were extracted from all human and animal tissue samples and cell lines, and were analyzed using a DNMT1 assay kit (Epignetek, Farmingdale, NY) following the manufacturer's protocol. The enzymatic activity of DNMT1 was detected with a microplate reader at 450 nm.

### 5-aza-2′-deoxycytidine treatment

5-aza-2′-deoxycytidine can induce selective degradation of DNMT1 by a proteasomal pathway [55]. *Men1* WT and *Men1* null MEF cells (1 × 10^5^ cells) were treated with 1 μM of 5-aza-2′-deoxycytidine, (Sigma, MO, USA) for 24 hours, and were subsequently cultured without *5-aza-2*′*-deoxycytidine*, followed by harvesting at 24 and 48 hours, respectively. Total mRNA from treated and untreated cells was extracted and the expression of *Sox* genes and β-catenin was quantified by qRT-PCR. The global DNA hypermethylation was determined in *Men1* null MEF cells with 5-aza-2′-deoxycytidine treatment compared to the *Men1* null cells without 5-aza-2′-deoxycytidine treatment by LUminometric Methylation Assay (LUMA) [56].

### *In vitro* cell proliferation assay

The human endocrine tumor cell line, BON-1, was treated for 48 hours with human Sox2 and Sox5 siRNA, respectively. The cell proliferation of BON-1 cells was detected by IF staining with Ki-67 marker (Abcam, Cambridge, MA) at pre- and post-treatment 48 hours with Sox2 and Sox5 siRNA, respectively.

### Statistical analysis

mRNA expression and enzymatic activity experiments were designed on true biological replicates (*n* = 3) with matched controls. Values are represented as the mean ± standard error of the mean (SEM). The difference between means was analyzed with either the two-tailed independent Student's *t* test for two group analysis, or by one-way analysis of variance (ANOVA) for multiple group analysis. If interactions were found, pair wise comparisons between group levels were calculated with Bonferroni correction for multiple testing. Statistical significance was defined at *P* value < 0.05.

As described above, DNA methylation status at individual CCGG sites was processed from the HELP-tagging assay and the resultant data were represented by values ranging from 0 to 100, corresponding to full methylation and non-methylation, respectively [29, 30]. The locus-specific values were compared between MEN1-parathyroid tumors, parathyroid adenoma, and carcinoma, against normal parathyroids, using paired *t* test. Differentially methylated loci were defined as those with a methylation change of 20% or greater and *P* value < 0.05. These loci were subsequently associated to gene promoter (defined as ± 1000 base pairs from the transcription starting sites) or gene body regions as described previously [57].

### Pathway analysis

We applied the Ingenuity Pathway Analysis (IPA, http://www.ingenuity.com, Redwood City, CA) to determine functional pathways that were enriched among genes with differentially methylated loci. The returned canonical pathways were filtered only for those related to tumorigenesis.

Differentially methylated loci were defined in terms of genomic context (e.g., occurring in a gene promoter, defined as ± 1000 base pairs from the transcription start site). We also analyzed tumor suppressors and PcGs in our data sets.

## SUPPLEMENTARY MATERIALS FIGURES AND TABLES




