# Supplementary material for: Loss of MEN1 activates DNMT1 implicating DNA hypermethylation as a driver of MEN1 tumorigenesis
Source: Oncotarget. 2016 Feb 9;7(11):12633–50. doi: 10.18632/oncotarget.7279 (PMC4914310; doi:10.18632/oncotarget.7279)
Supplement: Supplementary file 1 [file oncotarget-07-12633-s001.pdf]

Loss of *MEN1* activates DNMT1 implicating DNA hypermethylation as a driver of MEN1 tumorigenesis

Supplementary Materials

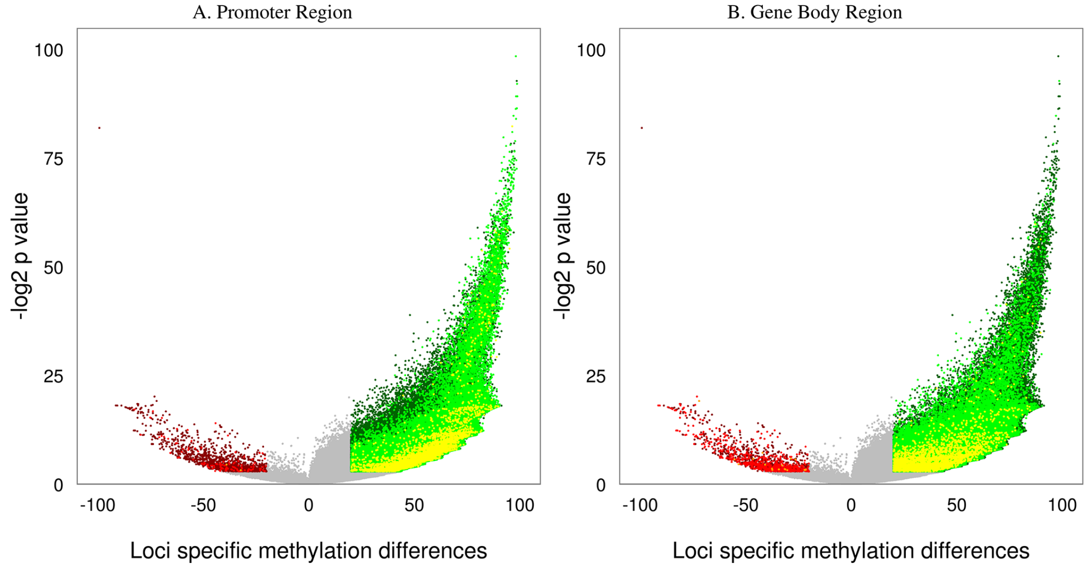

Supplementary Figure S1: Methylation in the promoter and gene body regions between MEN1-parathyroid tumor samples and controls. On the x-axis are the loci specific methylation differences between the two groups and on the y-axis are the -log2 of the corresponding *P* values of those mean differences. (**A**) Volcano plot showing loci specific methylation differences genome-wide (light gray) in the promoter region. On the right, the significant hypermethylated loci (dark green), located in the promoter region (light green) and mapping in the promoter region of tumor suppressor genes (yellow). On the left, the hypomethylated significant loci (dark red), located in the promoter region (light red) and mapping in the promoter region of tumor suppressor genes (orange). (**B**) Volcano plot showing loci specific methylation differences genome-wide (light gray) in the gene body region (same color assignment as in A).

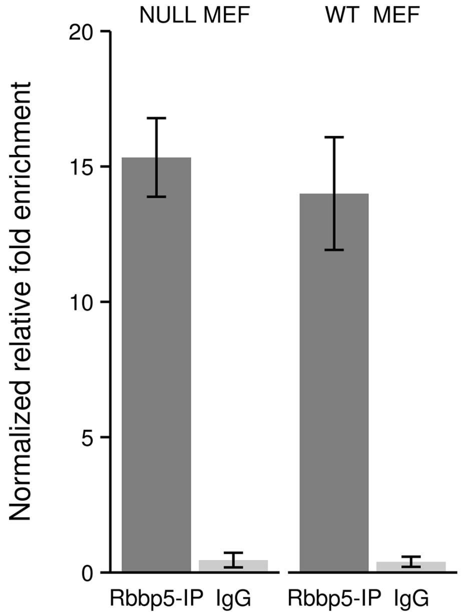

Supplementary Figure S2: ChIP-PCR analysis of Rbbp5 binding to DNMT1 promoter. The Rbbp5-IP products were amplified using quantitative real-time RT-PCR with primers corresponding to the promoter regions of the DNMT1 gene. The relative amount of DNA in the ChIP product was calculated using input for the normalization. IgG was used as a control.

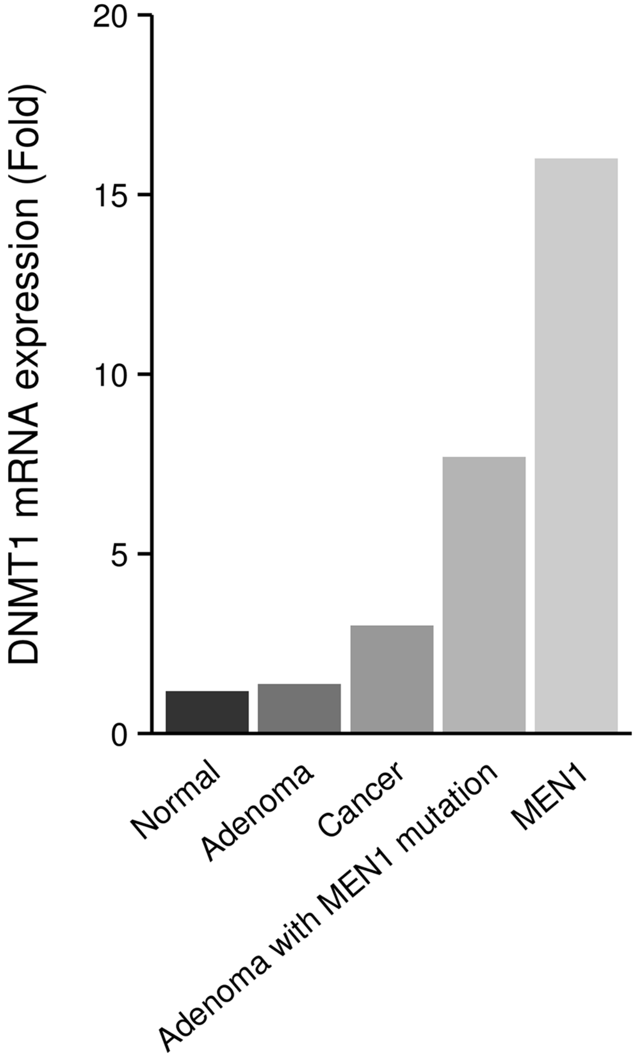

Supplementary Figure S3: Relative mRNA expression of DNMT1 gene in patients with parathyroid adenoma and parathyroid carcinoma. mRNA expression of the DNMT1 gene was not significantly increased in tumor tissues from the patients with parathyroid adenoma and parathyroid carcinoma when compared to normal parathyroid tissues. However, the mRNA expression of DNMT1 in the human parathyroid adenoma with a *MEN1* mutation in exon 6 was increased when compared to normal parathyroid tissues.

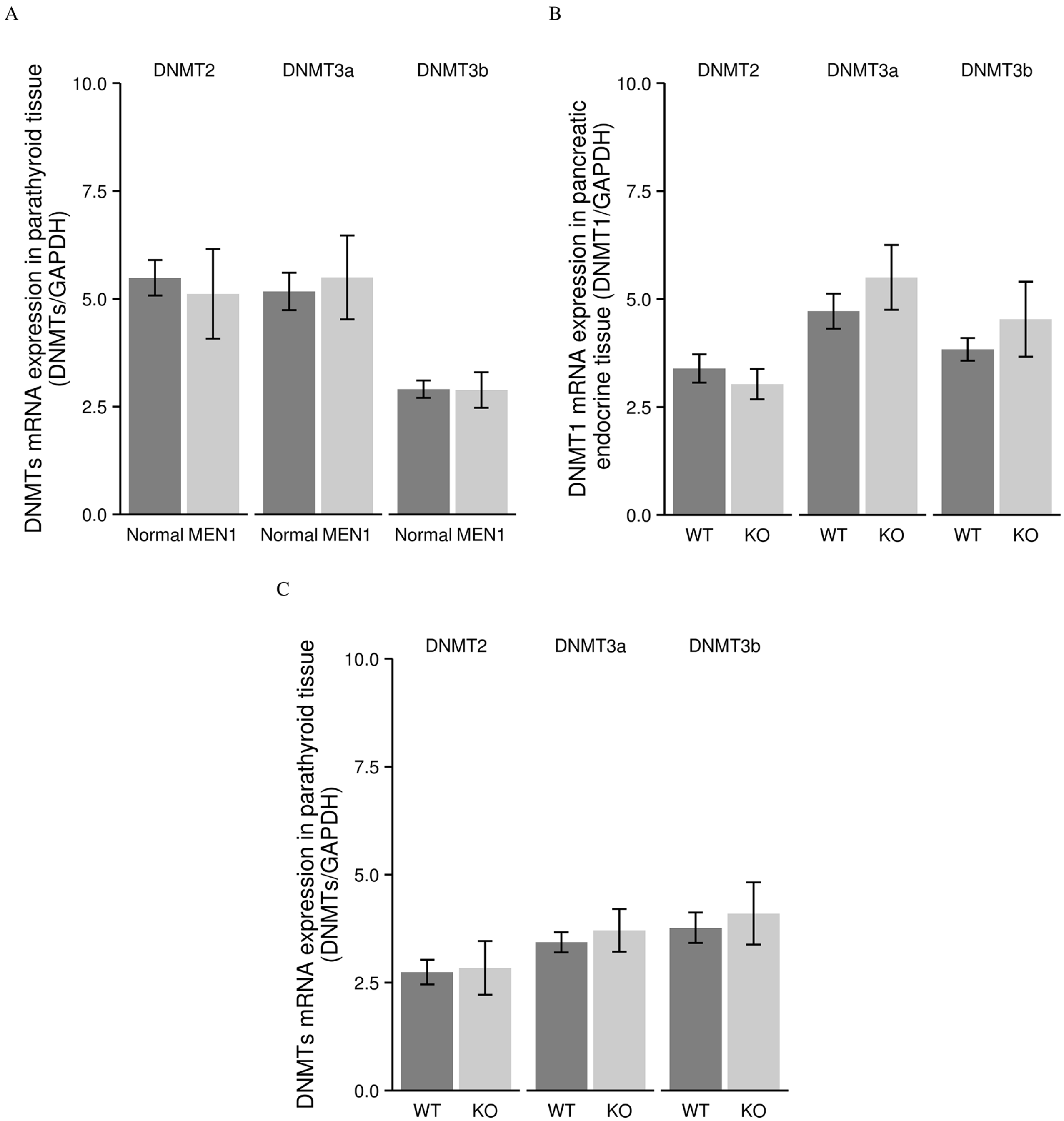

Supplementary Figure S4: The mRNA expression of DNMT2, DNMT3a, and DNMT3b genes in the endocrine tumor tissues from MEN1 patients and *Men1* KO mice. mRNA expression of DNMT2, DNMT3a, and DNMT3b was not significantly increased in the endocrine tumor tissues from MEN1 patients (**A**) and *Men1* KO mice in pancreatic endocrine tissue (**B**) and parathyroid tissues (**C**) when compared to normal endocrine tissues from human and WT mice.

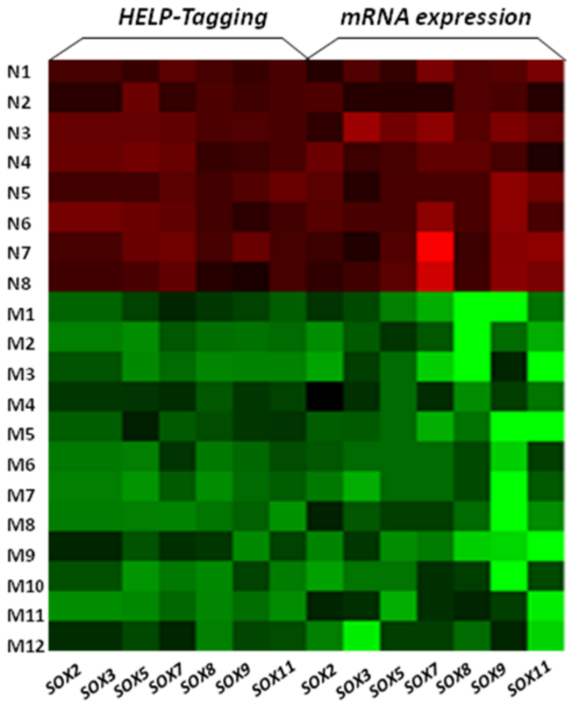

Sox gene	Correlation(R)
Sox2	0.8926
Sox3	0.9108
Sox5	0.8804
Sox7	0.9339
Sox8	0.9045
Sox9	0.9011
Sox11	0.9188

HELP-tagging
Red: Hypomethylation
Green: Hypermethylation

mRNA expression
Red: Upregulation
Green: Downregulation

Supplementary Figure S5: Heatmap of the correlation between HELP-tagging and mRNA expression in *Sox* gene family. The HELP-tagging and mRNA expression data on a gene expression heatmap shows a high correlation for the *Sox* gene family.

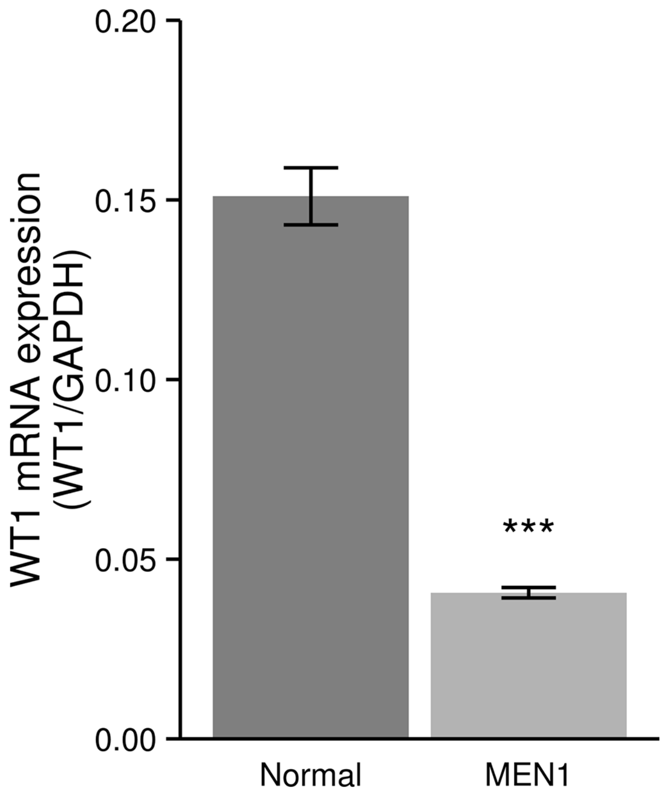

Supplementary Figure S6: The mRNA expression of WT1 in the MEN1-parathyroid tumors. The mRNA expression of WT1 is significantly decreased in MEN1-parathyroid tumors when compared to normal human parathyroid tissues. (MEN1: human MEN1 parathyroids; Normal: human normal parathyroids). (****P* < 0.001).

Supplementary Table S1: List of most methylated genes in the promoter region

Supplemental Table S2: Methylation differences in the promoter region of oncogene and tumor suppressor genes

Gene name	Chr	RefSeq ID	Methylation difference
TNFRSF19	chr13	NM_148957	69.03
SRC	chr20	NM_198291	64.58
TNFSF13B	chr13	NM_006573,NM_001145645	59.54
RAB9A	chrX	NM_004251	59.46
TSSC1	chr2	NM_003310	57.83
SOCS4	chr14	NM_199421,NM_007086,NM_001008396,NM_080867	57.01
BLCAP	chr20	NM_006698,NM_001167823,NM_001167821,NM_001167822, NM_001167820	56.16
BLCAP	chr20	NM_006698,NM_001167821,NM_001167822,NM_001167820	54.79
KIT	chr4	NM_000222,NM_001093772	54.53
CRKL	chr22	NM_005207	54.46
TNFSF11	chr13	NM_003701	54.16
MTUS1	chr8	NM_001001925,NM_001001924	53.14
CT62	chr15	NM_001102658	53.05
RAB34	chr17	NM_031934,NM_001144942,NR_024575,NM_001142625,NM_001 144943,NR_024579,NM_001142624	53.05
RAB43	chr3	NM_198490	52.88
SKIL	chr3	NM_005414,NM_001145098	52.85
BRMS1	chr11	NM_001024957,NM_015399	52.74
TNFRSF11A	chr18	NM_003839	52.66
FLI1	chr11	NM_002017	52.52
BMI1	chr10	NM_005180	52.48
MYBL1	chr8	NM_001080416,NM_001144755	52.17
CASC15	chr6	NR_015410	51.35
SUDS3	chr12	NM_022491	51.29
ORAOV1	chr11	NM_153451	51.28
BCAR1	chr16	NM_001170714,NM_001170718,NM_001170720	51.27
TNFRSF8	chr1	NM_001243	51.11
MAF	chr16	NM_005360,NM_001031804	51.1
MERTK	chr2	NM_006343	51.04
BRCA1	chr17	NM_007297,NM_007298,NR_027676,NR_003108,NM_007299,NM _007300,NM_007294	50.74
MCC	chr5	NM_002387	50.48
ERBB3	chr12	NM_001982,NM_001005915	50.4
FYN	chr6	NM_002037	50.31
TNFAIP3	chr6	NM_006290	50.31
FEV	chr2	NM_017521	50.25
ST7L	chr1	NM_138727,NM_138729,NM_138728,NM_017744,NM_006135	50.18
TNFSF10	chr3	NM_003810	50.01

RAB32	chr6	NM_006834	49.97
WT1	chr11	NM_024424,NM_024426,NM_000378,NR_023920,NM_024425	49.67
SUFU	chr10	NM_016169,NM_005736	49.53
TP53I13	chr17	NM_138349,NM_198147	49.52
RAP1B	chr12	NM_001010942,NM_015646	49.46
HIC1	chr17	NM_001098202,NM_006497	49.38
ERBB4	chr2	NM_005235,NM_001042599	49.32
ABL1	chr9	NM_005157	49.25
C1QTNF2	chr5	NM_031908	48.95
HIC1	chr17	NM_006497	48.84
ST5	chr11	NM_139157,NM_213618	48.57
VOPP1	chr7	NM_030796	48.52
MCC	chr5	NM_001085377	48.49
SUPT4H1	chr17	NM_003168	48.48
SOCS1	chr16	NM_003745	48.47
HIC1	chr17	NM_001098202	48.44
GLTSCR1	chr19	NM_015711	48.34
MAFF	chr22	NM_001161572,NM_001161574,NM_012323,NM_001161573	48.32
SOGA1	chr20	NM_080627	48.23
GREB1L	chr18	NM_001142966	48.17
TNFRSF12A	chr16	NM_016639	47.84
RAB35	chr12	NM_001167606,NM_006861	47.79
ST14	chr11	NM_021978	47.67
RAB6B	chr3	NM_016577	47.63
SHOC2	chr10	NM_007373,NR_024142,NR_024143,NR_024141,NR_024140, NR_015402	47.33
MYB	chr6	NM_001161657,NM_001161656,NM_001161659,NM_001161658,NM_001161660,NM_005375,NM_001130173,NM_001130172	47.21
SUPT7L	chr2	NM_014860,NM_018158	47.05
RELT	chr11	NM_152222,NM_032871	47.04
CASC4	chr15	NM_138423,NM_177974	46.97
RAP1A	chr1	NM_001010935,NM_002884	46.95
RAB33B	chr4	NM_031296	46.77
BCAR1	chr16	NM_014567,NM_001170717	46.76
SOCS2	chr12	NM_003877	46.66
SUV39H1	chrX	NM_003173	46.6
LYN	chr8	NM_001111097,NM_002350	46.57
FGFR1OP2	chr12	NM_001171887,NM_015633,NM_001171888,NM_018164	46.49
RABL3	chr3	NM_173825,NM_005513	46.49
CBL	chr11	NM_005188	46.44
BCAR1	chr16	NM_001170714,NM_001170718,NM_001170720,NM_001170716	46.37
SUV420H1	chr11	NM_016028,NM_017635	46.37
RAB28	chr4	NM_004249,NM_001159601,NM_001017979	46.19
RALA	chr7	NM_005402	46.19
REL	chr2	NM_002908	46.18

MAFA	chr8	NM_201589	46.17
LATS2	chr13	NM_014572	46.14
PVT1	chr8	NR_003367,NR_031609	46.05
TNFRSF21	chr6	NM_014452	45.99
SOCS6	chr18	NM_004232	45.98
TSG101	chr11	NM_006292	45.87
RAB27B	chr18	NM_004163	45.82
CNKSR2	chrX	NM_014927,NM_001168647,NM_001168649,NM_001168648	45.72
RAB8B	chr15	NM_016530	45.68
RAP2B	chr3	NM_002886	45.64
MYCL	chr1	NM_001033081,NM_005376,NM_001033082	45.59
TPRG1L	chr1	NM_182752	45.47
RAP2C	chrX	NM_021183	45.39
PIM2	chrX	NM_006875	45.37
SUV420H2	chr19	NM_032701	45.26
TNFRSF11B	chr8	NM_002546	45.21
SOCS7	chr17	NM_014598	45.11
FLI1	chr11	NM_002017,NM_001167681	45.1
RALB	chr2	NM_002881	44.98
MYC	chr8	NM_002467	44.92
KRAS	chr12	NM_033360,NM_004985	44.89
CBLB	chr3	NM_170662	44.88
LZTS2	chr10	NM_032429	44.83
RRAS	chr19	NM_006270,NM_021228	44.8
MAFB	chr20	NM_005461	44.76
TNFSF15	chr9	NM_005118	44.7
TNFAIP1	chr17	NM_021137,NM_174887	44.59
BCAR3	chr1	NM_003567	44.58
BRAF	chr7	NM_004333	44.27
CRK	chr17	NM_016823,NM_005206	44.08
RAB15	chr14	NM_198686	44.08
FGFR1OP	chr6	NM_007045,NM_194429	44.02
ERBB2	chr17	NM_004448	43.98
RAB5B	chr12	NM_002868	43.98
SFSWAP	chr12	NM_004592	43.87
RELA	chr11	NM_021975,NM_001145138	43.86
SET	chr9	NM_003011	43.76
CASC2	chr10	NR_026941,NM_014904,NR_026940,NR_026939	43.7
RAB11B	chr19	NM_004218	43.63
FOS	chr14	NM_005252	43.61
YES1	chr18	NM_005433	43.61
SEL1L	chr14	NM_005065	43.52
SOCS3	chr17	NM_003955	43.44
RAB12	chr18	NM_001025300	43.4
MRAS	chr3	NM_001085049,NM_012219	43.35

NKTR	chr3	NM_005385	43.3
HRASLS5	chr11	NM_054108,NM_001146728,NM_001146729	43.27
PTTG1IP	chr21	NM_004339	43.27
TNFRSF19	chr13	NM_018647	43.25
RAB1A	chr2	NM_004161,NM_015543	43.22
CDON	chr11	NM_016952	43.14
RAB7A	chr3	NM_004637	43.13
ETS1	chr11	NM_005238,NM_001162422	43.12
SRC	chr20	NM_005417,NM_198291	43.1
PTOV1	chr19	NM_017432	43.04
NTPCR	chr1	NM_032324	43.02
LATS1	chr6	NM_004690	43
CYLD	chr16	NM_001042412,NM_001042355,NM_015247	42.94
RAB11A	chr15	NM_004663	42.94
RAB40B	chr17	NM_006822	42.85
PIM3	chr22	NM_001001852	42.83
PRAC1	chr17	NM_032391	42.83
JUNB	chr19	NM_002229	42.77
ARAF	chrX	NM_001654	42.76
PIM1	chr6	NM_002648	42.66
ABL1	chr9	NM_007313	42.61
MOS	chr8	NM_005372	42.6
BOC	chr3	NM_033254	42.56
AKT2	chr19	NM_001626	42.55
SEL1L3	chr4	NM_015187	42.53
RAB23	chr6	NM_016277,NM_183227	42.47
WTIP	chr19	NM_001080436	42.46
COLCA2	chr11	NM_001136105,NM_207429	42.43
SCAI	chr9	NM_001144877,NM_173690	42.37
TP73	chr1	NM_005427,NM_017818	42.36
MTSS1	chr8	NM_014751	42.28
ETS2	chr21	NM_005239	42.27
RAP2A	chr13	NM_021033	42.27
SUPT16H	chr14	NM_007192	42.26
RAB13	chr1	NM_002870	42.18
RABL6	chr9	NM_001173989,NM_001173988,NM_024718,NM_017995,<br>NR_024580	42.18
ERG	chr21	NM_004449,NM_001136154	42.15
AKT1	chr14	NM_001014431,NM_005163,NM_001014432	41.82
DLEC1	chr3	NM_007335,NM_007337	41.81
RAB2A	chr8	NM_002865	41.72
FLI1	chr11	NM_001167681	41.71
RAB3C	chr5	NM_138453	41.7
RAB40C	chr16	NM_001172666,NM_001172665,NM_001172663,NM_001172664,<br>NM_021168	41.65

RET	chr10	NM_020975,NM_020630	41.52
WTAP	chr6	NM_152858,NM_004906,NM_152857	41.5
CASC3	chr17	NM_007359	41.47
SUPT3H	chr6	NM_003599,NM_181356	41.47
SUGT1	chr13	NM_001130912,NM_006704	41.37
SKI	chr1	NM_003036	41.36
RAB31	chr18	NM_006868	41.29
TP53INP1	chr8	NM_033285,NM_001135733	41.28
RAB6A	chr11	NM_198896,NM_002869	41.24
TP53BP2	chr1	NM_005426,NM_001031685	41.23
SET	chr9	NM_001122821	41.06
TP53BP1	chr15	NM_005657	40.99
FOSB	chr19	NM_001114171,NM_006732	40.92
RAB1B	chr11	NM_030981	40.89
RRAS2	chr11	NM_001102669,NM_001177315,NM_012250	40.78
TUSC3	chr8	NM_006765,NM_178234	40.74
RAF1	chr3	NM_002880	40.65
OVCA2	chr17	NM_080822	40.55
DEK	chr6	NM_001134709,NM_003472	40.43
TP73	chr1	NM_005427	40.39
RAB42	chr1	NM_152304	40.32
SUV39H2	chr10	NM_024670	40.29
JUND	chr19	NM_005354	40.26
SACM1L	chr3	NM_014016	40.25
TNFSF9	chr19	NM_003811	40.24
RAB4A	chr1	NM_004578	40.1
RAB7L1	chr1	NM_003929,NM_001135664,NM_001135663,NM_001135662	40.03
RAB3B	chr1	NM_002867	39.8
RSU1	chr10	NM_012425,NM_152724	39.71
TPD52	chr8	NM_001025253,NM_005079	39.71
JUN	chr1	NM_002228	39.47
TNFRSF1A	chr12	NM_001065	39.42
TPD52L1	chr6	NM_001003397,NM_001003396,NM_003287,NM_001003395	39.34
TNFRSF14	chr1	NM_003820	39.32
LDOC1	chrX	NM_012317	39.1
RAB5A	chr3	NM_004162	39.05
TACSTD2	chr1	NM_002353	39.05
RAB27A	chr15	NM_183235	39.02
GPS1	chr17	NM_212492,NM_002917,NM_004127	38.67
SUPV3L1	chr10	NM_003171	38.66
GPS2	chr17	NM_004489	38.58
TNFRSF10C	chr8	NM_003841	38.43
TP53INP2	chr20	NM_021202	38.39
HRAS	chr11	NM_005343,NM_001130442,NM_198075,NM_176795	38.33
BLCAP	chr20	NM_001167823	38.26

TNFRSF1B	chr1	NM_001066	38.24
RAB20	chr13	NM_017817	38.19
SMEK1	chr14	NM_032560	37.93
TUSC1	chr9	NM_001004125	37.92
RAB14	chr9	NM_016322	37.9
DLC1	chr8	NM_006094	37.84
KSR2	chr12	NM_173598	37.82
SUPT20H	chr13	NM_017569,NM_001014286	37.78
TNFRSF10D	chr8	NM_003840	37.78
RABL2B	chr22	NM_001130921,NM_001130920,NM_001130919,NM_001130922,NM_001003789,NM_007081,NM_001130923,NR_026982	37.76
ABL2	chr1	NM_001168237,NM_001168236,NM_001168238,NM_007314, NM_001136001	37.68
FES	chr15	NM_001143783,NM_001143785,NM_001143784,NM_002005	37.68
MYBL2	chr20	NM_002466	37.63
CBLC	chr19	NM_001130852,NM_012116	37.4
TP53I3	chr2	NM_004881,NM_147184	37.22
MDM2	chr12	NM_002392	36.94
RRAS	chr19	NM_006270	36.87
HRAS	chr11	NM_005343,NM_001130442,NM_176795	36.81
PTTG1	chr5	NM_004219	36.63
RAB4B	chr19	NM_016154	36.57
RAB26	chr16	NM_014353	36.44
MAFK	chr7	NM_002360	36.41
RAB39B	chrX	NM_171998	36.36
TNFRSF6B	chr20	NM_003823	36.33
MTUS2	chr13	NM_015233	35.96
RAB18	chr10	NM_021252	35.81
TNFRSF10A	chr8	NM_003844	35.79
TP53BP1	chr15	NM_001141980,NM_001141979	35.42
SNCG	chr10	NM_003087	35.14
ELK1	chrX	NM_005229,NM_001114123	35.11
BRMS1	chr11	NM_001024957,NM_006876,NM_015399	35.01
AKT1	chr14	NM_001014431,NM_001014432	34.98
SKIL	chr3	NM_005414,NM_001145098,NM_001145097	34.98
SIKE1	chr1	NM_025073,NM_001102396	34.83
RAN	chr12	NM_006325	34.7
RAB40C	chr16	NM_001172666,NM_001172665,NM_001172663,NM_001172664	34.58
RAB5C	chr17	NM_004583,NM_201434	34.27
CASC5	chr15	NM_144508,NM_170589	34.25
RAB37	chr17	NM_001006638,NM_001163990,NM_001163989	34.18
SDCCAG3	chr9	NM_006643,NM_001039708,NM_001039707,NM_015160	34.18
RTDR1	chr22	NM_014433	34.1
RAB3A	chr19	NM_002866	33.83
TNFAIP2	chr14	NM_006291	33.77

TNFAIP8L1	chr19	NM_001167942,NM_152362	33.75
RAB39A	chr11	NM_017516	33.74
HIC2	chr22	NM_015094	33.56
RAB19	chr7	NM_001008749	33.44
SMEK2	chr2	NM_001122964,NM_020463	33.42
SUPT5H	chr19	NM_001111020,NM_001130824,NM_001130825,NM_003169	33.42
TNFRSF18	chr1	NM_004195,NM_148901,NM_148902	33.34
TPD52L2	chr20	NM_199361,NM_199359,NM_199360,NM_199363,NM_199362, NM_003288	33.14
C1QTNF1	chr17	NM_030968,NM_198594	33.12
SOCS5	chr2	NM_014011,NM_144949	33.08
CASC1	chr12	NM_018272,NM_001082972,NM_001082973,NM_001001660	33.03
MACC1	chr7	NM_182762	32.82
MTSS1L	chr16	NM_138383	32.77
GLTSCR2	chr19	NM_015710	32.67
TNFRSF13C	chr22	NM_052945	32.61
PVT1	chr8	NR_031609	32.6
RAB37	chr17	NM_175738	32.4
MET	chr7	NM_001127500,NM_000245	32.13
C1QTNF4	chr11	NM_031909	31.96
RAB6C	chr2	NM_032144	31.79
RAB6A	chr2	NM_001077637	31.78
RELB	chr19	NM_006509	31.72
BRCA2	chr13	NM_000059	31.41
TNFAIP8	chr5	NM_014350	31.17
RAB34	chr17	NM_031934,NM_001144942,NR_000013,NR_024575,NM_0011426 25,NM_001144943,NR_024579,NM_001142624,NM_000984	30.99
RAB3D	chr19	NM_004283	30.95
RAB10	chr2	NM_016131	30.63
SOGA1	chr20	NM_199181,NM_080627	30.58
UCA1	chr19	NR_015379	30.58
VHL	chr3	NM_000551,NM_198156	30.51
RAB33A	chrX	NM_004794	30.46
RAB38	chr11	NM_022337	30.42
SUFU	chr10	NM_016169	30.17
TNFAIP8	chr5	NM_001077654	29.98
FGR	chr1	NM_005248	29.91
SAPCD2	chr9	NM_178448	29.91
RAB34	chr17	NM_031934,NM_001144942,NR_024575,NM_001142625,NM_001 144943,NR_024579,NM_001142624,NM_000984	29.44
BCAR1	chr16	NM_001170714,NM_001170718,NM_001170720,NM_001170715, NM_001170716	29.38
RAB8A	chr19	NM_005370	29.06
BCAR1	chr16	NM_014567,NM_001170717,NM_001170719	29.04
RAB9B	chrX	NM_016370	28.8

MAFG	chr17	NM_032711	28.33
TUSC5	chr17	NM_172367	28.07
BRMS1L	chr14	NM_032352	28.04
NRAS	chr1	NM_002524	28.04
C1QTNF6	chr22	NM_031910,NM_182486	27.97
TNFAIP8L3	chr15	NM_207381	27.78
TNFRSF10B	chr8	NM_003842,NR_027140,NM_147187	27.74
TSSC4	chr11	NM_005706	27.48
C1QTNF8	chr16	NM_207419	27.37
	chr20	NM_014731	27.14
DMBT1	chr10	NM_007329,NM_004406,NM_017579	26.9
BCAR1	chr16	NM_001170719	26.81
C1QTNF3	chr5	NM_181435,NM_030945	26.67
RAB36	chr22	NM_004914	26.62
CASC8	chr8	NR_024393	26.45
MRAS	chr3	NM_001085049	25.42
LDOC1L	chr22	NM_032287	25.04
SMEK3P	chrX	NR_002784	23.88
RAB17	chr2	NM_022449,NR_033308	22.7
RAB21	chr12	NM_014999	22.19
TP73	chr1	NM_001126240,NM_001126241,NM_001126242	21.94
USP4	chr3	NM_003363,NM_199443	21.71
RAB25	chr1	NM_020387	21.26
SPI1	chr11	NM_003120,NM_001080547	20.94
CT47A7	chrX	NM_001080140	20.57

Supplemental Table S3: Methylation differences in the promoter region of Polycomb group genes

Gene name	Chr	RefSeq ID	Methylation difference
BMI1	chr10	NM_005180	52.48
PCGF2	chr17	NM_007144	49.57
EZH2	chr7	NM_152998,NM_004456	48.48
RNF2	chr1	NM_007212	45.64
YY1	chr14	NM_003403	44.76
EPC1	chr10	NM_025209	44.32
SUZ12	chr17	NM_015355	41.08
PCGF5	chr10	NM_032373	40.43
RBM11	chr21	NM_144770	39.07
EPC2	chr2	NM_015630	38.8
RING1	chr6	NM_002931,NR_029633	38.8
PCGF6	chr10	NM_032154,NM_001011663	33.6
PCGF1	chr2	NM_032673	30.06
PCGF3	chr4	NM_006315	26.42
RING1	chr6	NM_002931	9.1

Supplementary Table S4: Validation data from HELP-tagging by mass array and pyrosequence assays

	Correlation Coefficient	Correlation Coefficient
	HELP-tagging/MassArray	HELP-tagging/Pyrosequence
Normal parathyroid tissues	−0.9284	−0.8505
parathyroid adenomas	−0.8678	−0.9298
parathyroid carcinomas	−0.9017	−0.9471
MEN1-parathyroid Hyperplasia	−0.8882	−0.9251
Total	−0.8943	−0.9233

Supplemental Table S5: Somatic mutations of MEN1 gene in sporadic parathyroid adenomas

Samples	Mutation	Location
Ad2	Silent mutation	Leu170Leu, Exon 2
Ad4	Silent mutation	Gly200Gly, Exon 2
Ad7	Silent mutation	Leu 223Leu, Exon 3
Ad7	Slient mutation	His328His, Exon 6
Ad10	Silent mutation	Asn373Asn, Exon 7
Ad17	Slient mutation	Gly390Gly, Exon 7
Ad18	Silent mutation	Gly470Gly, Exon 7
Ad19	Slient mutation	Ser510Ser, Exon 7
**Ad20**	**Missense mutation***	**Leu338Pro, Exon 6**

Supplemental Table S6: Primer sequences for Bisulfate-MassArray and Pyrosequencing assays

MassArray	
DL1.1	Sense: 5′-GGGAGTTTATTTAAGGTTTGTGTTAT-3′<br>Antisense: 5′-TTCCCTAAAAATAACTACTCCAACTC-3
DL1.2	Sense:5′-TTATAGTGGTAAATATGGGGAGAGA-3′<br>Antisense: 5′-CCAACCTAAAAACCAAAACATAATA-3′
DL1.3	Sense: 5′-TTTAAGGGTTGAGTTTTGTTAATAAG-3′<br>Antisense: 5′-AAAAATAACTTTTTACCCTCTCCTAC-3′
DL2.3	Sense: 5′-GTTTTTTTTAGTTGGGATAGGTTTTA-3<br>Antisense:5′-TCCAACTATCTTTCTACTCCCTAACT-3
DL3.1	Sense: 5′-GATTGTGGTGTTCATTTGTTTAGTTTA-3′<br>Antisense: 5′-ATAAATCCAAAAATTTTCTCATACCA-3′
DL3.2	Sense: 5′-TTTAATTTTTAATGATTGGAGATGTT-3′<br>Antisense: 5′-ACTAAACTTTTCATACCCACCTTCTA-3′
DL3.5	Sense: 5′-GTATTGAATAAATATTGGGAGGGTTA-3′<br>Antisense:5′-CACCACCTATAAAAAACTATCCTACA-3′
DL4.2	Sense: 5′-GGGGTGAGTAAGAAGTAGTAGGAGTA-3′<br>Antisense: 5′-TTCCCCTTTTAAAAATAAAACATAAA-3′
DL5.4	Sense: 5′-GATTGAATGGGGATGTTAATTAATAT-3′<br>Antisense: 5′-CTAACCCTAACCAAAAAAAACTTAAA-3

Pyrosequencing	
Pyroseq-1	Sense: 5′-AGGAATGGGGTAGGGAGGAT-3′<br>Antisense: 5′-(BIOTIN)- TACCCAATATCTCTCCCACACCTA-3′<br>Seq: 5′-GAGAGGTTGAGGGAAGT-3′
Pyroseq-2	Sense: 5′-TTTTTTAGTTGGGATAGGTTTTAG-3′<br>Antisense: 5′-(BIOTIN)- CCCACCACCCAATAATAAATAAC-3′<br>Seq: 5′-AGGAGTGTGTGAATTAGAGT-3′
Pyroseq-3	Sense: 5′-(BIOTIN)- TTGAATAAATATTGGGAGGGTTAG-3′<br>Antisense: 5′-CCCACCACCCAATAATAAATAAC-3′<br>Seq: 5′-AGGAGTGTGTGAATTAGAGT-3′
Pyroseq-4	Sense: 5′(BIOTIN)- GGGGTGAGTAAGAAGTAGTAGGA-3′<br>Antisense: 5′-ACTAAACCCCAAATATCAACAAAC-3′<br>Seq: 5′-AAAAAAAAAAAAAAACTAACC-3′

Supplemental Table S7: Primer sequences for Real-Time RT-PCR assay

mDNMT1	Sense: 5′-TGACAGTGGTGCTGAAGAAGCCAT-3′<br>Antisense: 5′-AGAATGGAGCCTCGAATTCTGAGA-3′
mDNMT2	Sense: 5'-GCTGCGAGAAAGTCATATCC-3<br>Antisense: 5'-TGGCATGGAGGGCTCATT-3
mDNMT3a	Sense: 5′-GGACAAGAATGCCACCAAAG-3′<br>Antisense: 5′-CCACTGAGAATTTGCCGTCT-3′
mDNMT3b	Sense: 5′-AGATCAAGCTCGCGACTCTC-3′<br>Antisense: 5-GACAGCTGGGCTTTCTGAA-3′
mMen1	Sense: 5′-GAAGATCATGCTTGGGTGGT-3′<br>Antisense: 5′-CAGGTCTGCCAAGTTCCCTA-3′
mRbbp5	Sense: 5′-CTTGACCAGAGGCATTGCTA-3′<br>Antisense: 5′-CTCTGGTCGCAATCTCCTGA-3′
mSox2	Sense: 5′-ACCAGCTCGCAGACCTACAT-3′<br>Antisense: 5′-GTGGGAGGAAGAGGTAACCA-3′
mSox3	Sense: 5′-GAACGCATCAGGTGAGAGAAG-3′<br>Antisense: 5′-GTCGGAGTGGTGCTCAGG-3′
mSox5	Sense: 5′-AGGATGTCTTCCAAGCGACC-3′<br>Antisense: 5′-CCTCAGAGTGAGGCTTGTTG-3
mSox7	Sense: 5′-CCCCATGAATGCCTTCATGG-3′<br>Antisense: 5′-AGGGTCTCTTCTGGGACAGT-3′
mSox8	Sense: 5′-CATCTCCATAACGCAGAGCT-3′<br>Antisense: 5′-TCGCCTTGGCTGGTATTTGT-3′
mSox9	Sense: 5′-AGCAAGACTCTGGGCAAGCT-3′<br>Antisense: 5′-TCCGTTCTTCACCGACTTCC-3′
mSox11	Sense: 5′-ACCCGGACTGGTGCAAGAC-3′<br>Antisense: 5′-CGACTGCTCCATGATCTTCCT-3′
mβ-catenin	Sense: 5′-AAGGAAGCTTCCAGACATGC-3′<br>Antisense: 5′-AGCTTGCTCTCTTGATTGCC-3′
mGAPDH	Sense: 5′-ATCACTGCCACCCAGAAGACT-3′<br>Antisense: 5′-CATGCCAGTGAGCTTCCCGTT-3′
hDNMT1	Sense: 5′-GAGGAAGCTGCTAAGGACTAGTTC-3′<br>Antisense: 5′-ACTGCACAATTTGATCACTAAATC-3′
hMEN1	Sense: 5′-GTACCTGGCTGGCTACCACTGTC-3′<br>Antisense: 5′-CCGGGCTTCCTCGCCCCACGG-3′

hRbbp5	Sense: 5′-TGGAGTTTGAGGAGGACCTC-3′<br>Antisense: 5′-CCAGATACAGCATCTCTCCC-3′
hSox2	Sense: 5′-GGGCAGCGTGTACTTATCCT-3′<br>Antisense: 5′-AGAACCCCAAGATGCACAAC-3′
hSox3	Sense: 5′-CCCATGAACGCCTTCATGGT-3′<br>Antisense: 5′-CTCGTCGATGAATGGTCGCT-3′
hSox5	Sense: 5′-CTCTCCACCTTCTCCATCTC-3′<br>Antisense: 5′-AATCTCACCAGCTGCTGGTA-3′
hSox7	Sense: 5′-TGAGCCAGGTGGAACTCCT-3′<br>Antisense: 5′-CTGGGAGACCGGAACATGC-3′
hSox8	Sense: 5′-TGTCTCCTTGCTGGCAGAGT-3′<br>Antisense: 5′-GAGCAACGAGCAACGTGATG-3′
hSox9	Sense: 5′-AGCGAACGCACATCAAGAC-3′<br>Antisense: 5′-GCTGTAGTGTGGGAGGTTGAA-3′
hSox11	Sense: 5′-GGTGGATAAGGATTTGGATTCG-3′<br>Antisense: 5′-GCTCCGGCGTGCAGTAGT-3′
hβ-catenin	Sense: 5-AAAATGGCAGTGCCTTTAG-3′<br>Antisense: 5′-TTTGAAGGCAGTCTGTCGTA-3′
hGAPDH	Sense: 5′-CGGGAAACTGTGGCGTGAT-3′<br>Antisense: 5′-ATGCCAGTGAGCTTCCCGT-3′
WT1	Sense: 5′-AGCACGGTCACTTTCGACG-3′<br>Antisense: 5′-GTTTGAAGGAATGGTTGGGGAA-3′

Supplemental Table S8: Primer sequences for ChIP-PCR assay

mDNMT1	Sense: 5′- TATAGCCAGGAGGTGTGGGTG -3′<br>Antisense: 5′-AAGGAGACCCCGGCTTTTT-3′
