# Supplementary material for: Loss of MEN1 activates DNMT1 implicating DNA hypermethylation as a driver of MEN1 tumorigenesis
Source: Oncotarget. 2016 Feb 9;7(11):12633–50. doi: 10.18632/oncotarget.7279 (PMC4914310; doi:10.18632/oncotarget.7279)
Supplement: Supplementary file 2 [file oncotarget-07-12633-s002.doc]

**Supplemental Table 1**: List of most methylated genes in the promoter region

| **Gene name** | **Chr** | **RefSeq ID** | **Methylation difference** |
| --- | --- | --- | --- |
| CITED2 | chr6 | NM_006079 | 89.59 |
| ZBTB37 | chr1 | NM_032522,NM_001122770 | 81.13 |
| GBP3 | chr1 | NM_018284 | 80.06 |
| TCAP | chr17 | NM_003673,NM_002686 | 79.47 |
| RNASEL | chr1 | NM_021133 | 78.47 |
| MFAP4 | chr17 | NM_002404 | 78.29 |
| HIST1H2BO | chr6 | NM_003527,NM_003514,NM_003535 | 77.72 |
| XAGE2B | chrX | NM_001079538 | 76.98 |
| XAGE2 | chrX | NM_001079538 | 76.98 |
| ARMCX5 | chrX | NM_001168482,NM_001168479,NM_001168480,NM_001168485,NM_022838,NM_001168478 | 76.41 |
| HOXB8 | chr17 | NM_024016,NM_004502 | 75.3 |
| HNRNPF | chr10 | NM_001098206,NM_001098205 | 74.05 |
| PPP1R10 | chr6 | NM_002714 | 73.91 |
| DUOXA2 | chr15 | NM_207581 | 73.18 |
| KCNJ16 | chr17 | NM_018658,NM_170741 | 71.81 |
| TCF7 | chr5 | NM_003202,NM_001134851,NM_201632,NM_213648,NM_201634,NR_033449,NM_201633 | 71.49 |
| ZNF484 | chr9 | NM_001007101,NM_031486 | 71.4 |
| MIR146B | chr10 | NR_030169,NM_024040 | 71.14 |
| EIF4A2 | chr3 | NR_002989,NR_031650,NR_002587,NR_002588,NR_002586 | 71.07 |
| SNORA81 | chr3 | NR_002989,NR_031650,NR_002587,NR_002588,NR_002586 | 71.07 |
| CTXN2 | chr15 | NM_001145668 | 70.86 |
| RNF5 | chr6 | NM_006913,NM_032741,NM_006411 | 70.46 |
| RBFOX2 | chr22 | NM_001031695,NM_001082576,NM_014309,NM_001082577 | 69.81 |
| MIR106A | chrX | NR_029523,NR_029949 | 69.74 |
| FXYD1 | chr19 | NM_005031 | 69.68 |
| TNFRSF19 | chr13 | NM_148957 | 69.03 |
| MAT2B | chr5 | NM_013283,NM_182796 | 68.68 |
| TGIF1 | chr18 | NM_170695,NM_003244,NM_173209,NM_173208,NM_173210,NM_173211 | 68.67 |
| MIR503 | chrX | NR_030228 | 68.46 |
| SNORD109B | chr15 | NR_001289,NR_001295 | 68.31 |
| SNORD109A | chr15 | NR_001289,NR_001295 | 68.31 |
| DUSP19 | chr2 | NM_080876,NM_001142314 | 68.1 |
| TSEN54 | chr17 | NM_207346 | 67.64 |
| PNPLA6 | chr19 | NM_001166113,NM_001166111,NM_001166112,NM_001166114 | 67.53 |
| UBE2D3 | chr4 | NM_181892,NM_181887,NM_181891 | 67.39 |
| IPMK | chr10 | NM_152230 | 67.14 |
| RNF5 | chr6 | NM_006913,NM_032741,NM_006411,NR_003129 | 67.03 |
| MUCL1 | chr12 | NM_058173 | 66.6 |
| TCEB3C | chr18 | NM_145653 | 66.48 |
| ISG20L2 | chr1 | NM_030980 | 66.4 |
| CDKN2B-AS1 | chr9 | NR_003529 | 66.26 |
| DNAJC16 | chr1 | NM_015291,NM_032996,NM_001229 | 66.24 |
| CTBP1 | chr4 | NM_001328,NM_001012614 | 65.94 |
| TMEM27 | chrX | NM_020665 | 65.94 |
| MOB3C | chr1 | NM_201403 | 65.87 |
| CHD2 | chr15 | NM_001271,NM_001042572 | 65.8 |
| CAV1 | chr7 | NM_001172897,NM_001172896 | 65.74 |
| PBX3 | chr9 | NM_006195,NR_024122,NR_024123 | 65.57 |
| MIR17HG | chr13 | NR_027349,NR_029487,NR_027350,NR_029488 | 65.35 |
| NEFM | chr8 | NM_001105541 | 65.16 |
| UBA1 | chrX | NM_153280 | 65.11 |
| SNORD42B | chr17 | NR_000013,NM_000984 | 64.99 |
| PPP2R2B | chr5 | NM_181676,NM_181674 | 64.87 |
| PENK | chr8 | NM_001135690 | 64.84 |
| NDUFAF3 | chr3 | NM_199074,NR_029948,NM_001009996 | 64.63 |
| NEFM | chr8 | NM_005382 | 64.6 |
| SRC | chr20 | NM_198291 | 64.58 |
| DOPEY1 | chr6 | NM_015018 | 64.32 |
| GOSR2 | chr17 | NM_001012511,NM_004287,NM_054022 | 64.22 |
| TRIM38 | chr6 | NM_006355 | 64.2 |
| CSMD3 | chr8 | NM_198123,NM_052900 | 64.1 |
| ZFAS1 | chr20 | NR_003604,NR_003605,NM_021035,NR_003606,NR_002433 | 63.93 |
| CCNE1 | chr19 | NM_001238 | 63.84 |
| FHL1 | chrX | NM_001159700,NM_001449,NM_001159703,NM_001159702 | 63.69 |
| YIPF5 | chr5 | NM_001024947,NM_030799,NM_020768 | 63.65 |
| ACAA1 | chr3 | NM_001607,NR_024024,NM_001130410 | 63.64 |
| HDGF | chr1 | NM_004494 | 63.64 |
| SLX4IP | chr20 | NM_001009608,NM_018848,NM_170784 | 63.47 |
| GID4 | chr17 | NM_024052 | 63.44 |
| NACC1 | chr19 | NM_052876,NM_001136035 | 63.43 |
| RTBDN | chr19 | NM_001080997 | 63.32 |
| UBE2D3 | chr4 | NM_181892 | 63.29 |
| PORCN | chrX | NM_203475,NM_203474 | 63.29 |
| FAM63A | chr1 | NM_001163258,NM_018379,NM_001040217,NM_021222 | 63.21 |
| RGMA | chr15 | NM_001166286,NM_001166288,NM_001166289 | 63.1 |
| CA10 | chr17 | NM_020178 | 63.07 |
| FAM211A-AS1 | chr17 | NR_003054,NR_003043,NR_002744 | 63.03 |
| MAN2B1 | chr19 | NM_000528,NM_001173498,NM_001099737,NR_029375,NM_016145,NM_032332 | 62.65 |
| FAM111A | chr11 | NM_022074,NM_198847 | 62.64 |
| MBNL1-AS1 | chr3 | NR_027037,NR_027038 | 62.6 |
| FHL1 | chrX | NM_001159700 | 62.5 |
| ZNF334 | chr20 | NM_018102,NM_199441 | 62.5 |
| PTCH1 | chr9 | NM_000264 | 62.21 |
| LIG4 | chr13 | NM_001098268,NM_032859 | 62.11 |
| QSER1 | chr11 | NM_001076786 | 62.03 |
| AKAP12 | chr6 | NM_144497 | 62.01 |
| GAS2 | chr11 | NM_001143830,NM_177553 | 61.97 |
| CDH12 | chr5 | NM_004061 | 61.93 |
| SYMPK | chr19 | NM_004819 | 61.74 |
| ZNF582 | chr19 | NM_144690 | 61.62 |
| RXRG | chr1 | NM_006917,NM_001009598 | 61.44 |
| CC2D2A | chr4 | NM_020785,NM_001164720,NM_001080522 | 61.35 |
| ANKAR | chr2 | NM_144708 | 61.12 |
| SP3 | chr2 | NM_001017371 | 61.07 |
| FOXO4 | chrX | NM_005938,NM_001170931 | 61.07 |
| KLK11 | chr19 | NM_144947,NM_001136032 | 61.04 |
| CSNK1E | chr22 | NM_152221 | 60.95 |
| CHST15 | chr10 | NM_015892 | 60.94 |
| PLEKHA8 | chr7 | NM_032639 | 60.88 |
| KDM2B | chr12 | NM_001005366 | 60.79 |
| MME | chr3 | NM_000902,NM_007287,NM_007288,NM_007289 | 60.7 |
| PRIM2 | chr6 | NM_000947 | 60.48 |
| FGFR2 | chr10 | NM_001144916,NM_001144915 | 60.47 |
| CABLES1 | chr18 | NM_001100619 | 60.39 |
| NHLRC2 | chr10 | NM_198514 | 60.39 |
|  | chrX | NR_026975 | 60.35 |
| GBE1 | chr3 | NM_000158 | 60.28 |
| RPS8 | chr1 | NM_001012,NR_000024,NR_000015,NR_001456 | 60.28 |
| EMC2 | chr8 | NM_014673 | 60.21 |
| TCEAL4 | chrX | NM_001006937,NM_001006936,NM_001006935,NM_024863 | 60.16 |
| SYT4 | chr18 | NM_020783 | 60.15 |
| C18orf32 | chr18 | NM_001035005,NR_031720 | 60.08 |
| ZNF436 | chr1 | NM_001077195 | 60.01 |
| EEF2 | chr19 | NM_001961,NR_002602 | 59.98 |
| LEF1-AS1 | chr4 | NR_029374,NM_001130714,NM_016269,NM_001130713 | 59.97 |
| ALDH16A1 | chr19 | NM_153329,NM_001145396 | 59.9 |
| LYRM7 | chr5 | NM_181705 | 59.89 |
| NDP | chrX | NM_000266 | 59.86 |
| ZFAND6 | chr15 | NM_019006 | 59.64 |
| TNFSF13B | chr13 | NM_006573,NM_001145645 | 59.54 |
| GOPC | chr6 | NM_020399,NM_001017408 | 59.47 |
| RAB9A | chrX | NM_004251 | 59.46 |
| TRIM7 | chr5 | NM_033342,NM_203293 | 59.4 |
| KCTD1 | chr18 | NM_001136205 | 59.38 |
| ACIN1 | chr14 | NM_001164816,NM_001164817 | 59.27 |
| CA10 | chr17 | NM_020178,NM_001082533 | 59.25 |
| ALDOA | chr16 | NM_184041,NM_001127617 | 59.21 |
| ZNF536 | chr19 | NM_014717 | 59.17 |
| SAYSD1 | chr6 | NM_018322 | 59.16 |
| RBBP6 | chr16 | NM_006910,NM_018703,NM_032626 | 59.15 |
| LYPD1 | chr2 | NM_144586 | 59.15 |
| SPG20 | chr13 | NM_015087,NM_001142296,NM_001142295 | 59.14 |
| YBEY | chr21 | NM_058181,NM_001006114,NM_003906 | 59.07 |
| SPRY1 | chr4 | NM_199327 | 59 |
| HIST1H2BD | chr6 | NM_138720,NM_021063 | 58.91 |
| SMAP2 | chr1 | NM_022733 | 58.89 |
| GAS5 | chr1 | NR_002578,NR_003942,NR_003943,NR_003941,NR_002579,NM_032522,NM_001122770 | 58.85 |
| CNOT2 | chr12 | NM_014515 | 58.84 |
| RFXAP | chr13 | NM_000538 | 58.79 |
| FAM214A | chr15 | NM_019600 | 58.71 |
| WRAP53 | chr17 | NM_001143991,NM_001143990,NM_001126114,NM_001126112,NM_001126113,NM_000546 | 58.66 |
| RASGRP2 | chr11 | NM_001098671 | 58.61 |
| KLHL7 | chr7 | NM_001031710,NR_033329,NM_001172428,NR_033328,NM_018846 | 58.56 |
| ALOXE3 | chr17 | NM_001165960 | 58.51 |
| PYURF | chr4 | NM_032906,NM_001042616 | 58.47 |
| SMC4 | chr3 | NM_005496 | 58.45 |
| BPI | chr20 | NM_001725 | 58.42 |
| ATRX | chrX | NM_000489,NM_138270 | 58.36 |
| LPCAT2 | chr16 | NM_017839 | 58.28 |
| SRP14 | chr15 | NM_003134 | 58.24 |
| HDHD2 | chr18 | NM_032124 | 58.21 |
| EGR2 | chr10 | NM_001136178 | 58.18 |
| CCNE2 | chr8 | NM_057749 | 58.13 |
| MAPT | chr17 | NM_016841,NM_016835,NM_005910,NM_001123067,NM_001123066,NM_016834,NR_024560,NR_024559 | 58.02 |
| CENPC | chr4 | NM_001812 | 58 |
| FGFRL1 | chr4 | NM_001004356 | 57.98 |
| MKLN1 | chr7 | NM_001145354,NR_015431,NR_024153 | 57.97 |
| TFAP2A | chr6 | NM_001032280,NM_003220 | 57.92 |
| MYO6 | chr6 | NM_004999 | 57.87 |
| ZBTB25 | chr14 | NM_006977 | 57.87 |
| SHISA2 | chr13 | NM_001007538 | 57.85 |
| TSSC1 | chr2 | NM_003310 | 57.83 |
| MEOX2 | chr7 | NM_005924 | 57.83 |
| RBMS3 | chr3 | NM_001003792,NM_014483,NM_001003793 | 57.81 |
| ARIH1 | chr15 | NM_005744 | 57.8 |
| NRXN1 | chr2 | NM_001135659,NM_004801 | 57.76 |
| GLTP | chr12 | NM_016433 | 57.74 |
| STAG1 | chr3 | NM_005862 | 57.68 |
| NAALAD2 | chr11 | NM_005467 | 57.6 |
| RPL41 | chr12 | NM_021104,NM_001035267,NM_032786 | 57.55 |
| ZNF503 | chr10 | NM_032772,NR_024421 | 57.55 |
| MRPL3 | chr3 | NM_007208 | 57.54 |
| TSLP | chr5 | NM_138551 | 57.5 |
| CAMK4 | chr5 | NM_001744 | 57.47 |
| ZIC4 | chr3 | NM_032153,NM_001168379 | 57.47 |
| CDC45 | chr22 | NM_003504,NM_005659,NM_001035247 | 57.42 |
| NR3C2 | chr4 | NM_000901,NM_001166104 | 57.28 |
| PLEKHA3 | chr2 | NM_019091 | 57.25 |
| RASL12 | chr15 | NM_016563 | 57.22 |
| ATP5D | chr19 | NM_001687,NM_152769,NM_001001975 | 57.19 |
| NOP10 | chr15 | NM_018648 | 57.19 |
| ZNF530 | chr19 | NM_020880 | 57.18 |
| EIF4H | chr7 | NM_022170,NM_031992 | 57.17 |
| MEIS2 | chr15 | NM_170677,NM_172315,NM_172316,NM_170675,NM_170674,NM_170676,NM_002399 | 57.11 |
| ZNF197 | chr3 | NM_001024855,NM_006991 | 57.1 |
| RPL7A | chr9 | NR_002447,NM_000972,NM_181491,NM_133640,NR_000017,NR_002448 | 57.1 |
|  | chr9 | NR_002447,NM_000972,NM_181491,NM_133640,NR_000017,NR_002448 | 57.1 |
| SNORD24 | chr9 | NR_002447,NM_000972,NM_181491,NM_133640,NR_000017,NR_002448 | 57.1 |
| ZFP37 | chr9 | NM_003408 | 57.09 |
| C10orf25 | chr10 | NM_001039380,NM_006963 | 57.07 |
| CEP44 | chr4 | NM_001040157,NM_001145314,NM_012180 | 57.02 |
| SOCS4 | chr14 | NM_199421,NM_007086,NM_001008396,NM_080867 | 57.01 |
| USP37 | chr2 | NM_020935,NM_005444 | 57 |
| ERLEC1 | chr2 | NM_001127397,NM_015701,NM_001127398,NM_016115,NM_145863 | 56.98 |
| INSC | chr11 | NM_001042536 | 56.93 |
| FAM135B | chr8 | NM_015912 | 56.93 |
| C10orf107 | chr10 | NM_173554 | 56.92 |
| LMAN1 | chr18 | NM_005570 | 56.9 |
| SNORD43 | chr22 | NR_002439,NR_000026 | 56.9 |
| FOXA1 | chr14 | NM_004496 | 56.87 |
| CFC1 | chr2 | NM_032545 | 56.87 |
| CXorf58 | chrX | NM_001169574,NM_024122,NR_026545,NM_152761 | 56.8 |
| TGIF1 | chr18 | NM_003244,NM_173209,NM_173207,NM_173208 | 56.8 |
| GFOD2 | chr16 | NM_030819,NR_027398,NR_027399 | 56.78 |
| PTGER4 | chr5 | NM_000958 | 56.77 |
| TMEM26 | chr10 | NM_178505 | 56.76 |
| RPS25 | chr11 | NM_001028,NM_016146 | 56.74 |
| AMER3 | chr2 | NM_001105195,NM_001105193,NM_001105194 | 56.74 |
| POP5 | chr12 | NM_198202,NM_015918 | 56.74 |
| GORASP2 | chr2 | NM_015530 | 56.73 |
| RASA2 | chr3 | NM_006506 | 56.72 |
| PPP1R9A | chr7 | NM_001166162,NM_001166163,NM_017650,NM_001166160,NM_001166161 | 56.7 |
| ZIC4 | chr3 | NM_001168379,NM_001168378 | 56.7 |
| ESR1 | chr6 | NM_001122742 | 56.69 |
| SRD5A1 | chr5 | NM_001047,NM_017755 | 56.66 |
| TOMM70A | chr3 | NM_014820,NM_001085451 | 56.65 |
| BEND6 | chr6 | NM_152731,NM_001144769 | 56.62 |
| NAGS | chr17 | NM_153006 | 56.58 |
|  | chr12 | NR_024457 | 56.57 |
| SPON2 | chr4 | NM_012445 | 56.53 |
| FAM150A | chr8 | NM_207413 | 56.52 |
| CBR1 | chr21 | NM_001757 | 56.51 |
| HEBP2 | chr6 | NM_014320 | 56.48 |
| LRFN5 | chr14 | NM_152447 | 56.43 |
| NRG3 | chr10 | NM_001010848,NM_001165972,NM_001165973 | 56.39 |
| TASP1 | chr20 | NM_017714 | 56.39 |
| NR2F2 | chr15 | NM_021005 | 56.38 |
| MIR574 | chr4 | NR_030300,NM_138389,NR_033290 | 56.36 |
|  | chrX | NM_001013742 | 56.35 |
| OGT | chrX | NM_181672,NM_181673 | 56.35 |
| SPATA2 | chr20 | NM_006038,NM_001135773 | 56.34 |
| BTG4 | chr11 | NM_017589,NR_029840,NR_029839 | 56.32 |
| MRPS11 | chr15 | NM_022839,NM_022163,NM_176805 | 56.32 |
| EDNRB | chr13 | NM_001122659,NM_003991 | 56.3 |
| ZNF580 | chr19 | NM_001163423,NM_016535 | 56.3 |
| SALL4 | chr20 | NM_020436 | 56.29 |
| MAGI3 | chr1 | NM_001142782,NM_152900 | 56.28 |
| ZFP69 | chr1 | NM_198494 | 56.28 |
| FANCI | chr15 | NM_001113378,NM_018193 | 56.26 |
| ZKSCAN3 | chr6 | NM_024493 | 56.26 |
| FRZB | chr2 | NM_001463 | 56.23 |
| TBC1D5 | chr3 | NM_014744,NM_001134380 | 56.22 |
| SMIM4 | chr3 | NM_001124767,NM_022908 | 56.21 |
| EIF4A1 | chr17 | NR_002918,NM_001416 | 56.21 |
| SNORA48 | chr17 | NR_002918,NM_001416 | 56.21 |
| FST | chr5 | NM_006350,NM_013409 | 56.19 |
| SNORD114-29 | chr14 | NR_003222,NR_003219,NR_003220,NR_003221 | 56.19 |
| GNG2 | chr14 | NM_053064 | 56.18 |
| BLCAP | chr20 | NM_006698,NM_001167823,NM_001167821,NM_001167822,NM_001167820 | 56.16 |
| FAM160B1 | chr10 | NM_001135051,NM_020940 | 56.14 |
| C11orf88 | chr11 | NM_001100388,NM_207430,NM_017589,NR_029840,NR_029839 | 56.1 |
| ST8SIA6 | chr10 | NM_001004470 | 56.07 |
| MRPS24 | chr7 | NM_032014 | 56.05 |
| PTCH1 | chr9 | NM_000264,NM_001083605,NM_001083604 | 56.04 |
| DNAJC3 | chr13 | NM_006260 | 56.04 |
| NR2F2 | chr15 | NM_021005,NM_001145156 | 56.04 |
| SLC22A18 | chr11 | NM_183233 | 55.98 |
| NRP2 | chr2 | NM_201279,NM_201267,NM_201264,NM_018534,NM_003872,NM_201266 | 55.97 |
| ARSI | chr5 | NM_001012301 | 55.93 |
| SLC38A1 | chr12 | NM_001077484,NM_030674 | 55.92 |
| TBL1XR1 | chr3 | NM_024665 | 55.91 |
| FYTTD1 | chr3 | NM_032288,NM_001145642,NM_001011537,NR_027840 | 55.85 |
| PYROXD1 | chr12 | NM_024854 | 55.84 |
| SLIT3 | chr5 | NM_003062 | 55.83 |
| SMARCD3 | chr7 | NM_001003801 | 55.81 |
| SALL1 | chr16 | NM_002968 | 55.81 |
| BANK1 | chr4 | NM_017935,NM_001127507 | 55.8 |
| ZEB2 | chr2 | NM_014795,NM_001171653,NR_033258 | 55.78 |
| BBX | chr3 | NM_001142568,NM_020235 | 55.77 |
| HDHD1 | chrX | NM_012080,NM_001135565 | 55.76 |
| EIF1B | chr3 | NM_005875 | 55.75 |
| WT1-AS | chr11 | NR_023920 | 55.75 |
| PRDM1 | chr6 | NM_001198 | 55.74 |
| RANBP3L | chr5 | NM_145000,NM_001161429 | 55.73 |
| LDB1 | chr10 | NM_001113407 | 55.72 |
| ANAPC15 | chr11 | NM_014042 | 55.69 |
| NMRAL1 | chr16 | NM_020677,NM_002134,NM_001127204 | 55.69 |
| SNORD35A | chr19 | NR_000018,NR_000019,NR_000020 | 55.69 |
| RPL13A | chr19 | NR_000018,NR_000019,NR_000020 | 55.69 |
| MIR129-2 | chr11 | NR_029697 | 55.67 |
| MSH5 | chr6 | NM_025259,NM_002441,NM_172166,NM_001288,NM_172165 | 55.64 |
| RGP1 | chr9 | NM_001080496,NM_020944 | 55.63 |
| DGKI | chr7 | NM_004717 | 55.62 |
| OTUD3 | chr1 | NM_015207 | 55.6 |
| NDNL2 | chr15 | NM_138704 | 55.58 |
| NAV2 | chr11 | NM_001111019 | 55.57 |
|  | chr8 | NR_015374 | 55.54 |
| CYP4A11 | chr1 | NM_000778 | 55.52 |
| BCOR | chrX | NM_001123385,NM_017745 | 55.52 |
| SESN3 | chr11 | NM_144665 | 55.52 |
| PCDH19 | chrX | NM_001105243,NM_020766 | 55.49 |
| MLLT3 | chr9 | NM_004529 | 55.47 |
| IL5RA | chr3 | NM_175727,NM_175725,NM_175726,NM_175724,NM_175728,NM_000564 | 55.47 |
| ENC1 | chr5 | NM_003633 | 55.45 |
| ARID3B | chr15 | NM_006465 | 55.45 |
| COPS8 | chr2 | NM_006710,NM_198189 | 55.45 |
| KLHL4 | chrX | NM_019117,NM_057162 | 55.44 |
| KCNJ6 | chr21 | NM_002240 | 55.42 |
| IMPACT | chr18 | NM_018439 | 55.41 |
| HMGA1 | chr6 | NM_145899,NM_002131,NM_145903,NM_145905,NM_145901,NM_145902 | 55.41 |
| HIST2H2AC | chr1 | NM_003517,NM_003528 | 55.39 |
| GATA6 | chr18 | NM_005257 | 55.36 |
| TMEM132E | chr17 | NM_207313 | 55.35 |
| PIR | chrX | NM_001018109,NM_003662 | 55.34 |
| NOP56 | chr20 | NM_006392,NR_027700,NR_003078,NR_031699 | 55.33 |
| ZBBX | chr3 | NM_024687 | 55.33 |
| ISLR2 | chr15 | NM_001130136,NR_027073,NM_001130137 | 55.28 |
| KLHL31 | chr6 | NM_001003760 | 55.26 |
| NAP1L3 | chrX | NM_004538,NM_173698,NM_001171109,NM_001171110,NM_001171111 | 55.21 |
| DCAF12 | chr9 | NM_015397 | 55.21 |
| ADAM12 | chr10 | NM_003474,NM_021641 | 55.2 |
| GFI1 | chr1 | NM_001127216,NM_001127215 | 55.17 |
| TWF1 | chr12 | NM_002822 | 55.17 |
| ASCL1 | chr12 | NM_004316 | 55.17 |
| PDIA3P1 | chr1 | NR_002305 | 55.17 |
| ETV4 | chr17 | NM_001986,NM_001079675 | 55.16 |
| MINPP1 | chr10 | NM_004897 | 55.15 |
| KIAA0020 | chr9 | NM_014878 | 55.15 |
| CYYR1 | chr21 | NM_052954 | 55.15 |
| PTHLH | chr12 | NM_198964,NM_002820,NM_198966,NM_198965 | 55.14 |
| NLGN4X | chrX | NM_020742 | 55.13 |
| GABRA2 | chr4 | NM_000807,NM_001114175 | 55.12 |
| MB21D2 | chr3 | NM_178496 | 55.12 |
| EBF1 | chr5 | NM_024007 | 55.11 |
| OTX2-AS1 | chr14 | NR_029385 | 55.11 |
| CYFIP2 | chr5 | NM_001037333,NM_001037332 | 55.09 |
| KCNJ8 | chr12 | NM_004982 | 55.09 |
| ATP7B | chr13 | NM_000053,NM_001005918,NM_001004127 | 55.08 |
| IGFBP3 | chr7 | NM_000598,NM_001013398 | 55.08 |
| SPRY2 | chr13 | NM_005842 | 55.08 |
| TENC1 | chr12 | NM_015319,NM_198316,NM_170754 | 55.08 |
| C16orf72 | chr16 | NM_014117 | 55.07 |
| ADCY5 | chr3 | NM_183357 | 55.05 |
| ITPKA | chr15 | NM_002220 | 55.04 |
| SCARB2 | chr4 | NM_005506 | 55.04 |
| UBA6 | chr4 | NM_018227,NR_015439 | 55.04 |
| SGK1 | chr6 | NM_001143676 | 55.03 |
| KLHL14 | chr18 | NM_020805 | 55.03 |
| HNRNPH2 | chrX | NM_001032393,NM_019597,NM_000169 | 55.01 |
| DIAPH3 | chr13 | NM_001042517 | 55 |
| EIF4A2 | chr3 | NM_001967,NR_002587 | 55 |
| HIST1H1E | chr6 | NM_005321,NM_138720,NM_021063 | 54.99 |
| C9orf171 | chr9 | NM_207417 | 54.98 |
| RELN | chr7 | NM_005045,NM_173054 | 54.97 |
| SUCO | chr1 | NM_016227,NM_014283 | 54.96 |
| FTSJ3 | chr17 | NM_017647 | 54.96 |
| EHBP1 | chr2 | NM_015252,NM_001142616,NM_001142614 | 54.95 |
| CDK6 | chr7 | NM_001145306,NM_001259 | 54.9 |
| AP1S2 | chrX | NM_003916 | 54.89 |
| RAD23B | chr9 | NM_002874 | 54.88 |
| LRRC29 | chr16 | NM_001004055,NM_012163,NM_014187 | 54.87 |
| PPM1N | chr19 | NM_001080401 | 54.85 |
| PCDH10 | chr4 | NM_032961,NM_020815 | 54.81 |
| MIR503HG | chrX | NR_024607,NR_030228,NR_029946 | 54.81 |
| CDC14B | chr9 | NM_001077181 | 54.8 |
| ADARB1 | chr21 | NM_001160230,NM_015833,NM_001112,NR_027674,NR_027672,NR_027673,NM_015834 | 54.8 |
| BLCAP | chr20 | NM_006698,NM_001167821,NM_001167822,NM_001167820 | 54.79 |
| TICAM2 | chr5 | NM_021649 | 54.78 |
| AFAP1 | chr4 | NM_198595,NM_001134647 | 54.78 |
| NT5E | chr6 | NM_002526 | 54.77 |
| HTR2C | chrX | NM_000868 | 54.76 |
| IL32 | chr16 | NM_001012636,NM_001012635,NM_001012634,NM_001012632 | 54.75 |
| KAT2B | chr3 | NM_003884 | 54.75 |
| GDNF | chr5 | NM_199231 | 54.75 |
| SNORA37 | chr18 | NR_002970,NM_015832,NM_003927 | 54.75 |
| ILK | chr11 | NM_004517,NM_015324,NM_001014794,NM_001014795 | 54.74 |
| RALYL | chr8 | NM_001100392,NM_173848,NM_001100393 | 54.72 |
| ANO1 | chr11 | NM_018043 | 54.72 |
| ARHGEF12 | chr11 | NM_015313 | 54.71 |
| PRR7 | chr5 | NM_001174101,NM_030567 | 54.69 |
| DRAM1 | chr12 | NM_018370 | 54.69 |
| HIPK1 | chr1 | NM_198268,NM_152696 | 54.69 |
| MYADM | chr19 | NM_001020821,NM_138373,NM_001020819,NM_001020818 | 54.66 |
| SDK2 | chr17 | NM_001144952 | 54.64 |
| GOLM1 | chr9 | NM_016548,NM_177937 | 54.63 |
| ZNF683 | chr1 | NM_173574,NM_001114759 | 54.63 |
| VTN | chr17 | NM_000638,NM_015077 | 54.59 |
| SEBOX | chr17 | NM_000638,NM_015077 | 54.59 |
| KCNMB2 | chr3 | NM_181361 | 54.59 |
| BRS3 | chrX | NM_001727 | 54.57 |
| NHLH2 | chr1 | NM_001111061 | 54.56 |
| FDFT1 | chr8 | NM_004462 | 54.55 |
| MIR17HG | chr13 | NR_027349,NR_029487,NR_027350,NR_029489,NR_029488 | 54.55 |
| ARHGAP12 | chr10 | NM_018287 | 54.54 |
| KIT | chr4 | NM_000222,NM_001093772 | 54.53 |
| SP3 | chr2 | NM_003111,NM_001172712 | 54.52 |
| RCL1 | chr9 | NM_005772 | 54.52 |
| ARMCX3 | chrX | NM_016607,NM_177948,NM_177947 | 54.52 |
| C16orf71 | chr16 | NM_139170,NM_133450 | 54.52 |
| ALKBH6 | chr19 | NM_198867,NM_032878 | 54.52 |
| LINC00461 | chr5 | NR_024384 | 54.52 |
| NTRK2 | chr9 | NM_001018065,NM_001007097,NM_001018066 | 54.49 |
| BAHD1 | chr15 | NM_014952 | 54.49 |
| ZNF460 | chr19 | NM_006635 | 54.48 |
| KRR1 | chr12 | NM_007043 | 54.47 |
| CRKL | chr22 | NM_005207 | 54.46 |
| NIPAL4 | chr5 | NM_001099287,NM_001172292 | 54.45 |
| BUB1 | chr2 | NM_004336 | 54.45 |
| FBXO3 | chr11 | NM_033406,NM_012175 | 54.45 |
| UNC5C | chr4 | NM_003728 | 54.44 |
| MAMDC2 | chr9 | NM_153267 | 54.43 |
| ATP8B4 | chr15 | NM_024837 | 54.42 |
| GTDC1 | chr2 | NM_001164629 | 54.41 |
| ARHGEF9 | chrX | NM_001173479,NR_031567 | 54.41 |
| SMAD5 | chr5 | NM_005903,NM_001001420,NM_001001419,NR_026763 | 54.41 |
| LHX5 | chr12 | NM_022363 | 54.41 |
| VAMP4 | chr1 | NM_003762 | 54.39 |
| SIAH2 | chr3 | NM_005067 | 54.39 |
| TLX1 | chr10 | NM_005521 | 54.38 |
| C11orf88 | chr11 | NM_001100388,NM_207430,NR_029840,NR_029839 | 54.36 |
| RNF14 | chr5 | NM_004290,NM_183398,NM_183400,NM_183401 | 54.36 |
| ISLR2 | chr15 | NM_020851,NM_001130137,NM_001130138 | 54.36 |
| ACVR2B | chr3 | NM_001106,NR_028389 | 54.34 |
| SNHG12 | chr1 | NR_003035,NR_002976,NR_002987,NR_024127 | 54.33 |
| ZNF232 | chr17 | NM_014519 | 54.31 |
| HOXB2 | chr17 | NM_002145 | 54.3 |
| MEIS2 | chr15 | NM_172315,NM_172316 | 54.3 |
| SYNDIG1 | chr20 | NM_024893 | 54.29 |
| LINC00667 | chr18 | NR_015389,NR_026849 | 54.27 |
| NOL6 | chr9 | NM_022917,NM_139235 | 54.26 |
| GMNC | chr3 | NM_001146686 | 54.25 |
| HIST1H3I | chr6 | NM_003533,NM_003546 | 54.25 |
| YOD1 | chr1 | NM_018566 | 54.25 |
| POLI | chr18 | NM_007195 | 54.24 |
| SLC44A1 | chr9 | NM_080546 | 54.24 |
| LINC00909 | chr18 | NR_024484 | 54.23 |
| DDX3X | chrX | NM_001356 | 54.22 |
| WLS | chr1 | NM_024911,NM_001002292 | 54.22 |
| NR6A1 | chr9 | NM_033334,NM_001489 | 54.2 |
| EVC | chr4 | NM_153717,NM_001166136 | 54.2 |
| NOS1 | chr12 | NM_000620 | 54.16 |
| TNFSF11 | chr13 | NM_003701 | 54.16 |
| CITED1 | chrX | NM_001144885,NM_001144887 | 54.15 |
| KDM5C | chrX | NM_004187,NM_001146702 | 54.15 |
| BAG3 | chr10 | NM_004281 | 54.13 |
| GREM1 | chr15 | NM_013372 | 54.12 |
| PLEC | chr8 | NM_201378,NM_000445 | 54.12 |
| CCND3 | chr6 | NM_001136017,NM_001136126 | 54.1 |
| MECOM | chr3 | NM_001164000 | 54.1 |
| CCDC85B | chr11 | NM_006848 | 54.1 |
| SEZ6L2 | chr16 | NM_012410,NM_001114100,NM_201575,NM_001114099 | 54.1 |
| COL9A2 | chr1 | NM_001852 | 54.09 |
| MMP2 | chr16 | NM_004530,NM_001127891 | 54.09 |
| GRB7 | chr17 | NM_001030002 | 54.08 |
| OTX2 | chr14 | NM_021728,NR_029385 | 54.08 |
| DUOXA2 | chr15 | NM_207581,NM_014080 | 54.07 |
| CYP26A1 | chr10 | NM_057157 | 54.06 |
| CASR | chr3 | NM_000388 | 54.05 |
| SIAH3 | chr13 | NM_198849 | 54.05 |
| MAGI1 | chr3 | NM_001033057,NM_004742,NM_015520 | 54.04 |
| CYP26A1 | chr10 | NM_057157,NM_000783 | 54.03 |
| ZFP36L1 | chr14 | NM_004926 | 54.02 |
| CDC14B | chr9 | NM_033331,NM_003671 | 54.02 |
| PPM1J | chr1 | NM_005167 | 54.01 |
| SLC9A8 | chr20 | NM_015266 | 54.01 |
| TUBB | chr6 | NM_178014 | 54.01 |
| SCAMP2 | chr15 | NM_005697 | 53.99 |
| MSMO1 | chr4 | NM_006745,NM_001017369 | 53.98 |
| ITGA11 | chr15 | NM_001004439 | 53.96 |
| POLR2K | chr8 | NM_005034 | 53.96 |
| ZNF518A | chr10 | NM_014803 | 53.96 |
| CRH | chr8 | NM_000756 | 53.95 |
| ATP6V0C | chr16 | NM_001694 | 53.92 |
| SPAG6 | chr10 | NM_012443,NM_172242 | 53.91 |
| FOXA2 | chr20 | NM_021784 | 53.91 |
|  | chr12 | NR_027249,NM_003299 | 53.91 |
| PDGFRA | chr4 | NM_006206 | 53.9 |
| NPTX2 | chr7 | NM_002523 | 53.88 |
| PAWR | chr12 | NM_002583 | 53.88 |
| RNF146 | chr6 | NM_030963 | 53.87 |
| MBNL1-AS1 | chr3 | NR_027037,NM_207292,NM_021038,NR_027038 | 53.87 |
| LINC00936 | chr12 | NR_028138 | 53.87 |
| NOX1 | chrX | NM_007052,NM_013955 | 53.86 |
| MIR375 | chr2 | NR_029867 | 53.86 |
| MYO1E | chr15 | NM_004998 | 53.84 |
| IREB2 | chr15 | NM_004136 | 53.83 |
| PON1 | chr7 | NM_000446 | 53.82 |
| ZNF516 | chr18 | NM_014643 | 53.82 |
| ST6GAL1 | chr3 | NM_173216,NM_173217 | 53.8 |
| GPX3 | chr5 | NM_002084 | 53.78 |
| TCF7 | chr5 | NM_003202,NM_001134851,NM_201632,NM_213648,NM_201634,NM_201633 | 53.78 |
| LYPLA2 | chr1 | NM_007260 | 53.76 |
| PTPN6 | chr12 | NM_080548,NM_138425 | 53.74 |
| NDFIP2 | chr13 | NM_001161407,NM_019080 | 53.73 |
| FGF12 | chr3 | NM_004113 | 53.73 |
| CDH6 | chr5 | NM_004932 | 53.72 |
| SATB2 | chr2 | NM_001172509 | 53.71 |
| GAB3 | chrX | NM_001081573,NM_080612 | 53.7 |
| NKAIN3 | chr8 | NM_173688 | 53.67 |
| GALNT10 | chr5 | NM_198321 | 53.67 |
| TMEM51 | chr1 | NM_001136217,NR_027136,NM_001136216,NM_001136218,NM_018022 | 53.66 |
| MSI2 | chr17 | NM_138962,NM_170721 | 53.66 |
| QSOX1 | chr1 | NM_002826,NM_001004128 | 53.65 |
| TSC22D3 | chrX | NM_004089 | 53.63 |
| CITED2 | chr6 | NM_001168389,NM_006079,NM_001168388 | 53.62 |
| TRAF3 | chr14 | NM_145726,NM_003300,NM_145725 | 53.62 |
| ATP1B1 | chr1 | NM_001677,NM_001001787 | 53.61 |
| SYT1 | chr12 | NM_005639,NM_001135805 | 53.6 |
| SREK1IP1 | chr5 | NM_173829,NM_005869 | 53.59 |
| TMEM30B | chr14 | NM_001017970 | 53.57 |
| FBXL15 | chr10 | NM_024326,NM_002779 | 53.57 |
| RERG | chr12 | NM_032918 | 53.57 |
| H2AFV | chr7 | NM_138635,NM_201517,NM_201516,NM_201436,NM_012412 | 53.57 |
| NDUFAF3 | chr3 | NM_199070,NM_199074,NM_199073,NM_018114,NR_029690,NM_199069 | 53.55 |
| SBK1 | chr16 | NM_001024401 | 53.54 |
| ZIC1 | chr3 | NM_003412,NM_032153 | 53.54 |
| KIAA1033 | chr12 | NM_015275 | 53.54 |
| ADH1B | chr4 | NM_000668 | 53.53 |
| MPPED2 | chr11 | NM_001145399 | 53.53 |
| IRS2 | chr13 | NM_003749 | 53.53 |
| MNX1 | chr7 | NM_001165255 | 53.52 |
| LRRIQ1 | chr12 | NM_001079910,NM_001100917,NM_032165 | 53.51 |
| ARAP2 | chr4 | NM_015230 | 53.5 |
| RCBTB2 | chr13 | NM_001268 | 53.48 |
| UGCG | chr9 | NM_003358 | 53.48 |
| LFNG | chr7 | NM_001040168,NM_001040167 | 53.47 |
| FAM163A | chr1 | NM_173509 | 53.46 |
| TMCC3 | chr12 | NM_020698 | 53.45 |
| PSMG1 | chr21 | NM_203433,NM_003720 | 53.45 |
| NR2F1 | chr5 | NM_005654 | 53.44 |
| AGBL3 | chr7 | NM_178563 | 53.44 |
| CAV1 | chr7 | NM_001172897,NM_001753,NM_001172895,NM_001172896 | 53.43 |
| GALNT3 | chr2 | NM_004482 | 53.43 |
| EBF2 | chr8 | NM_022659 | 53.43 |
| C2orf88 | chr2 | NM_001042519,NM_032321 | 53.42 |
| RFX7 | chr15 | NM_022841 | 53.42 |
| C19orf43 | chr19 | NM_024038 | 53.42 |
| MYL12B | chr18 | NM_033546,NM_001144946,NM_001144944,NM_001144945 | 53.42 |
| DLG2 | chr11 | NM_001142702 | 53.41 |
| MTHFS | chr15 | NM_006441 | 53.41 |
| LRRC28 | chr15 | NM_144598,NM_001040659,NM_001040658,NM_001040657,NM_001040656,NM_001040655,NM_001040660,NM_022905 | 53.41 |
| C15orf39 | chr15 | NM_015492 | 53.4 |
| ACBD6 | chr1 | NM_032360 | 53.4 |
| ANKRD17 | chr4 | NM_032217,NM_198889 | 53.39 |
| NKX1-2 | chr10 | NM_001146340 | 53.38 |
| ZEB1 | chr10 | NM_001174096,NM_001174095,NR_024286,NR_024287,NR_024285,NR_024284,NM_001128128,NM_030751,NM_001174093,NM_001174094 | 53.38 |
| ZNF112 | chr19 | NM_013380,NM_001083335 | 53.38 |
| RIMS3 | chr1 | NM_014747 | 53.38 |
| GPBP1 | chr5 | NM_022913 | 53.38 |
| WASF3 | chr13 | NM_006646 | 53.36 |
| RBM17 | chr10 | NM_032905,NM_001145547 | 53.36 |
| IQSEC2 | chrX | NM_001111125 | 53.34 |
| FANCL | chr2 | NM_001114636,NM_018062 | 53.34 |
| KLF10 | chr8 | NM_005655 | 53.34 |
| KIAA1328 | chr18 | NM_020776,NM_015476 | 53.34 |
| GAS5 | chr1 | NR_002578,NR_003942,NR_003941,NR_002579,NM_032522,NM_001122770 | 53.33 |
| SAMD8 | chr10 | NM_001174156,NM_144660 | 53.32 |
| KPNA4 | chr3 | NM_002268 | 53.3 |
| DDR1 | chr6 | NM_013993 | 53.3 |
| TRAPPC2 | chrX | NM_014563,NM_001011658,NM_001128835,NM_003611 | 53.3 |
| MRPS16 | chr10 | NM_016065 | 53.3 |
| ZNF395 | chr8 | NM_018660 | 53.3 |
| CPNE4 | chr3 | NM_130808 | 53.3 |
| TMEFF1 | chr9 | NM_003692 | 53.29 |
| ARHGEF3 | chr3 | NM_019555 | 53.29 |
| PPFIBP2 | chr11 | NM_003621 | 53.24 |
| LRAT | chr4 | NM_004744 | 53.24 |
| HMMR | chr5 | NM_001142557,NM_145266,NM_012484,NM_012485,NM_001142556 | 53.23 |
| COL4A6 | chrX | NM_033641,NM_000495,NM_001847,NM_033381,NM_033380 | 53.23 |
| RPH3A | chr12 | NM_001143854,NM_014954 | 53.21 |
| KDM6A | chrX | NM_021140 | 53.2 |
| SALL3 | chr18 | NM_171999 | 53.2 |
| PTP4A1 | chr6 | NM_003463 | 53.18 |
| PDZRN3 | chr3 | NM_015009 | 53.18 |
| HOXB5 | chr17 | NM_002147,NR_033204,NR_033201,NR_033202,NR_033203 | 53.17 |
| VIM | chr10 | NM_003380 | 53.17 |
| ZNF451 | chr6 | NM_015555,NM_001031623 | 53.17 |
| NGLY1 | chr3 | NM_018297,NM_001145295,NM_001145293 | 53.17 |
| FUNDC1 | chrX | NM_173794 | 53.17 |
| BRD2 | chr6 | NM_001113182 | 53.15 |
| MTUS1 | chr8 | NM_001001925,NM_001001924 | 53.14 |
| TFAP2D | chr6 | NM_172238 | 53.13 |
| LHX9 | chr1 | NM_020204 | 53.12 |
| CCDC140 | chr2 | NM_153038 | 53.12 |
| C3orf70 | chr3 | NM_001025266 | 53.1 |
| DLG2 | chr11 | NM_001142699,NM_018480 | 53.1 |
| SMAD4 | chr18 | NM_005359 | 53.09 |
| GAB2 | chr11 | NM_080491 | 53.08 |
| CT62 | chr15 | NM_001102658 | 53.05 |
| RIMBP3B | chr22 | NM_001128635 | 53.05 |
| RGL3 | chr19 | NM_001161616,NM_001035223 | 53.05 |
| RAB34 | chr17 | NM_031934,NM_001144942,NR_024575,NM_001142625,NM_001144943,NR_024579,NM_001142624 | 53.05 |
| CDKN2B-AS1 | chr9 | NR_003529,NM_058195 | 53.05 |
| MEIS2 | chr15 | NM_170677,NM_170675,NM_170674,NM_170676,NM_002399 | 53.04 |
| TRMT2A | chr22 | NM_182984,NM_022727,NM_002882 | 53.04 |
| CCT7 | chr2 | NM_001166285,NR_029402,NM_006429,NM_032319,NM_001009570,NR_029403,NM_001166284 | 53.03 |
| EIF2B2 | chr14 | NM_014239 | 53 |
| DTX4 | chr11 | NM_015177 | 53 |
| TRIM72 | chr16 | NM_001008274,NM_152901 | 52.99 |
| CDH2 | chr18 | NM_001792 | 52.99 |
| USP6NL | chr10 | NM_014688 | 52.98 |
| FRMPD1 | chr9 | NM_014907 | 52.98 |
| TMED7 | chr5 | NM_181836,NM_001164468,NM_001164469 | 52.98 |
| PRKDC | chr8 | NM_001081640,NM_006904,NM_005914,NM_182746 | 52.97 |
| TSLP | chr5 | NM_033035 | 52.97 |
| KAL1 | chrX | NM_000216 | 52.96 |
| GTF2F2 | chr13 | NM_004128 | 52.96 |
| SPTBN1 | chr2 | NM_178313 | 52.95 |
| SATB2 | chr2 | NM_015265 | 52.94 |
| EFHC2 | chrX | NM_025184 | 52.93 |
| NELFE | chr6 | NM_002904,NM_006929,NR_031601 | 52.9 |
| MID1IP1 | chrX | NM_021242 | 52.9 |
| UBE2D3 | chr4 | NM_181892,NM_181887,NM_181891,NM_181889,NM_181888,NM_003340,NM_181886 | 52.9 |
| LPAR1 | chr9 | NM_001401,NM_057159 | 52.89 |
| RAB43 | chr3 | NM_198490 | 52.88 |
| AQP4-AS1 | chr18 | NR_026908,NM_004028 | 52.88 |
| RGL1 | chr1 | NM_015149,NM_005717 | 52.87 |
| DLG3 | chrX | NM_020730 | 52.87 |
| C18orf25 | chr18 | NM_145055,NM_001008239 | 52.86 |
| GOLGB1 | chr3 | NM_004487 | 52.85 |
| SKIL | chr3 | NM_005414,NM_001145098 | 52.85 |
| FLRT2 | chr14 | NM_013231 | 52.85 |
| NFKBIA | chr14 | NM_020529 | 52.85 |
| FOXI2 | chr10 | NM_207426 | 52.85 |
| ARHGAP5 | chr14 | NM_001173,NR_027263,NM_001030055 | 52.84 |
| VAV3 | chr1 | NM_006113 | 52.84 |
| RAX | chr18 | NM_013435 | 52.84 |
| HMGN3 | chr6 | NM_138730,NM_004242 | 52.84 |
| PPT2 | chr6 | NM_138717,NM_005155 | 52.83 |
| METTL5 | chr2 | NM_014168 | 52.82 |
| KCNJ9 | chr1 | NM_004983 | 52.81 |
| MAGED2 | chrX | NM_014599,NM_177433,NM_201222 | 52.81 |
| M6PR | chr12 | NM_002355 | 52.8 |
| SLC1A5 | chr19 | NM_005628,NM_001145144 | 52.8 |
| PLK2 | chr5 | NM_006622 | 52.8 |
| CLMP | chr11 | NM_024769 | 52.8 |
| WNT5A | chr3 | NM_003392 | 52.78 |
| STT3A | chr11 | NM_152713 | 52.78 |
| LRP5 | chr11 | NM_002335 | 52.77 |
| CCNDBP1 | chr15 | NM_012142,NR_027513,NM_037370,NR_027514 | 52.77 |
| ANKS1B | chr12 | NM_152788 | 52.77 |
| TK2 | chr16 | NM_001172644,NM_001172645,NM_001172643,NM_004614 | 52.76 |
| EPDR1 | chr7 | NM_017549 | 52.76 |
| ZC3H14 | chr14 | NM_024824,NM_001160104,NM_001160103,NM_207661,NM_207660 | 52.76 |
| TMTC1 | chr12 | NM_175861 | 52.75 |
| BRMS1 | chr11 | NM_001024957,NM_015399 | 52.74 |
| PTPRD | chr9 | NM_002839 | 52.74 |
| PAPD7 | chr5 | NM_006999,NM_001171805 | 52.74 |
| FBXW2 | chr9 | NM_012164,NR_027442 | 52.74 |
| CCDC132 | chr7 | NM_024553,NM_017667 | 52.73 |
| PATL1 | chr11 | NM_152716 | 52.73 |
| DLX6 | chr7 | NM_005222 | 52.72 |
| RXFP1 | chr4 | NM_021634 | 52.71 |
| CAMKV | chr3 | NM_024046 | 52.71 |
| GNAS | chr20 | NM_001077489,NM_080426,NM_000516,NM_001077488 | 52.7 |
| CUL1 | chr7 | NM_003592 | 52.7 |
| CELF4 | chr18 | NM_001025088,NM_020180,NM_001025087,NM_001025089 | 52.69 |
| MORC2 | chr22 | NM_014941,NR_002323 | 52.69 |
| MAPK6 | chr15 | NM_002748 | 52.67 |
| NFATC4 | chr14 | NM_001136022,NM_004554 | 52.66 |
| TNFRSF11A | chr18 | NM_003839 | 52.66 |
| MT2A | chr16 | NM_005953 | 52.65 |
| ENPEP | chr4 | NM_001977 | 52.64 |
| KIAA1199 | chr15 | NM_018689 | 52.63 |
| ZFP30 | chr19 | NM_014898 | 52.62 |
| PIGK | chr1 | NM_005482 | 52.61 |
| GRIK3 | chr1 | NM_000831 | 52.6 |
| KCNA4 | chr11 | NM_002233 | 52.6 |
| REV1 | chr2 | NM_016316,NM_001037872 | 52.6 |
| PRRX1 | chr1 | NM_022716,NM_006902 | 52.6 |
| FBN1 | chr15 | NM_000138 | 52.59 |
| BSCL2 | chr11 | NM_001122955,NM_001130702 | 52.59 |
| DPYSL2 | chr8 | NM_001386 | 52.59 |
| LINC00461 | chr5 | NR_024383 | 52.59 |
| GTF2F1 | chr19 | NM_002096 | 52.57 |
| ZIC3 | chrX | NM_003413 | 52.57 |
| NCOR1 | chr17 | NM_006311 | 52.57 |
| KIF21B | chr1 | NM_017596 | 52.57 |
| SEMA6A | chr5 | NM_020796 | 52.57 |
| H2AFY2 | chr10 | NM_018649 | 52.56 |
| SLC7A14 | chr3 | NM_020949 | 52.56 |
| RASGRP2 | chr11 | NM_001098670,NM_001098671,NM_153819 | 52.55 |
| RTKN | chr2 | NM_033046,NM_001015055,NM_001015056 | 52.55 |
| RPL38 | chr17 | NM_000999,NM_001035258 | 52.52 |
| FLI1 | chr11 | NM_002017 | 52.52 |
| GPBP1 | chr5 | NM_001127235 | 52.51 |
| SHMT2 | chr12 | NM_005412,NM_001166356,NR_029417 | 52.51 |
| REV3L | chr6 | NM_002912 | 52.5 |
| CHL1 | chr3 | NM_006614 | 52.5 |
| UGGT2 | chr13 | NM_020121 | 52.5 |
| CACNA1A | chr19 | NM_023035,NM_001127222,NM_001174080,NM_001127221,NM_000068 | 52.5 |
| DSCC1 | chr8 | NM_024094 | 52.5 |
| ADAMTS12 | chr5 | NM_030955 | 52.5 |
| MEF2C | chr5 | NM_002397 | 52.49 |
| PTPRM | chr18 | NM_002845,NM_001105244 | 52.49 |
| FBXO10 | chr9 | NM_012166 | 52.49 |
| HIPK2 | chr7 | NM_022740,NM_001113239 | 52.49 |
| DUSP5 | chr10 | NM_004419 | 52.48 |
| BMI1 | chr10 | NM_005180 | 52.48 |
| TRIM26 | chr6 | NM_003449 | 52.47 |
| XPR1 | chr1 | NM_004736,NM_001135669 | 52.47 |
| SRRM4 | chr12 | NM_194286 | 52.47 |
| CUL4B | chrX | NM_001079872 | 52.46 |
| ARHGAP31 | chr3 | NM_020754 | 52.46 |
| C7orf60 | chr7 | NM_152556 | 52.46 |
| GMDS | chr6 | NM_001500 | 52.45 |
| TMEM185A | chrX | NM_001174092,NM_032508 | 52.44 |
| TMED3 | chr15 | NM_007364 | 52.44 |
| LHX9 | chr1 | NM_001014434 | 52.43 |
| MPPE1 | chr18 | NM_023075 | 52.43 |
| JKAMP | chr14 | NM_016475,NM_001098625,NM_144581 | 52.42 |
| FGF13 | chrX | NM_001139498,NM_001139500,NM_001139501 | 52.41 |
| MTM1 | chrX | NM_000252 | 52.4 |
| FGFR1 | chr8 | NM_001174065,NM_001174066,NM_001174067 | 52.4 |
| CUX1 | chr7 | NM_001913,NM_181552,NM_181500 | 52.4 |
| DLL1 | chr6 | NM_005618 | 52.4 |
| ZNF675 | chr19 | NM_138330 | 52.39 |
| MAPK3 | chr16 | NM_001040056,NM_002746,NM_001109891 | 52.38 |
| WSB2 | chr12 | NM_018639 | 52.38 |
| NAV2 | chr11 | NM_145117,NM_182964 | 52.38 |
|  | chr2 | NR_033389,NM_014562 | 52.38 |
| WBP5 | chrX | NM_001006612,NM_016303,NM_001006614,NM_001006613 | 52.37 |
| LCOR | chr10 | NM_001170766,NM_001170765,NM_032440 | 52.37 |
| USP9X | chrX | NM_001039591,NM_001039590 | 52.36 |
| C17orf96 | chr17 | NM_001130677 | 52.36 |
| AMER1 | chrX | NM_152424 | 52.36 |
| FGF3 | chr11 | NM_005247 | 52.35 |
| C16orf45 | chr16 | NM_033201 | 52.34 |
| IGF1R | chr15 | NM_000875 | 52.33 |
| ARHGEF2 | chr1 | NM_001162384,NM_001162383,NM_004723 | 52.33 |
| GRIA3 | chrX | NM_007325,NM_000828 | 52.33 |
| CHRDL2 | chr11 | NM_015424 | 52.33 |
| ZNF624 | chr17 | NM_020787 | 52.33 |
| MCHR2 | chr6 | NM_001040179,NM_032503 | 52.32 |
| LIMD1 | chr3 | NM_014240 | 52.32 |
| TNPO2 | chr19 | NM_001136196,NM_013433,NM_001136195 | 52.31 |
| ZBTB14 | chr18 | NM_001143823,NM_003409,NR_026569 | 52.31 |
| SEC11A | chr15 | NM_014300 | 52.31 |
| JAM2 | chr21 | NM_021219 | 52.31 |
| SCARNA13 | chr14 | NR_003002,NM_016417,NR_001459,NR_003138 | 52.31 |
| IGFBP5 | chr2 | NM_000599 | 52.3 |
| EXPH5 | chr11 | NM_015065 | 52.3 |
| MIR548AA2 | chr1 | NR_030385 | 52.3 |
| MIR548D2 | chr1 | NR_030385 | 52.3 |
| ERCC5 | chr13 | NM_000123 | 52.29 |
| PEX2 | chr8 | NM_000318,NM_001079867,NM_001172086,NM_001172087 | 52.29 |
| LMTK3 | chr19 | NM_001080434 | 52.29 |
| CABLES1 | chr18 | NM_001100619,NR_023359 | 52.29 |
| STXBP5 | chr6 | NM_139244,NM_001127715 | 52.29 |
| GALR2 | chr17 | NM_003857 | 52.28 |
| VPS45 | chr1 | NM_007259 | 52.28 |
| MAGEH1 | chrX | NM_014061 | 52.28 |
| SLC25A37 | chr8 | NM_016612 | 52.28 |
| TLE4 | chr9 | NM_007005 | 52.27 |
| ZNF385B | chr2 | NM_152520,NR_031659 | 52.27 |
| TBL1X | chrX | NM_001139466,NM_001139467,NM_001139468,NM_005647 | 52.26 |
| GDF9 | chr5 | NM_005260,NM_014402 | 52.26 |
| RFWD2 | chr1 | NM_001001740,NM_022457 | 52.25 |
| N4BP2 | chr4 | NM_018177,NR_027277 | 52.25 |
| HMX1 | chr4 | NM_018942 | 52.25 |
| SRI | chr7 | NM_003130 | 52.24 |
| TOPBP1 | chr3 | NM_007027 | 52.24 |
| VWC2 | chr7 | NM_198570 | 52.23 |
| ZNF71 | chr19 | NM_021216 | 52.22 |
| MCTP1 | chr5 | NM_024717 | 52.22 |
| ARF5 | chr7 | NM_001662 | 52.21 |
| GABARAPL2 | chr16 | NM_007285 | 52.21 |
| MSL2 | chr3 | NM_018133,NM_001145417 | 52.21 |
| KCTD12 | chr13 | NM_138444 | 52.21 |
| FOXB2 | chr9 | NM_001013735 | 52.19 |
| HOXB7 | chr17 | NM_004502 | 52.19 |
| ST8SIA3 | chr18 | NM_015879 | 52.19 |
| MAML3 | chr4 | NM_018717 | 52.19 |
| MBIP | chr14 | NM_016586,NM_001144891 | 52.18 |
| MYBL1 | chr8 | NM_001080416,NM_001144755 | 52.17 |
| NME1-NME2 | chr17 | NM_002512,NM_001018137,NM_001018139,NM_001018138 | 52.17 |
| FGF14 | chr13 | NM_004115 | 52.17 |
| NCBP2 | chr3 | NM_007362,NM_001042540,NR_024388 | 52.17 |
| HOXA10 | chr7 | NM_018951 | 52.16 |
| NEFH | chr22 | NM_021076 | 52.16 |
| ZNF670 | chr1 | NM_033213 | 52.16 |
| HMX3 | chr10 | NM_001105574 | 52.15 |
| TTF2 | chr1 | NM_003594 | 52.15 |
| NEUROG1 | chr5 | NM_006161 | 52.15 |
| BCL11A | chr2 | NM_022893,NM_138559,NM_018014 | 52.15 |
| NIPAL1 | chr4 | NM_207330 | 52.15 |
| FGFR2 | chr10 | NM_001144919,NM_001144918,NM_001144915,NM_000141,NM_022970,NM_001144917 | 52.14 |
| PLCL1 | chr2 | NM_006226 | 52.14 |
|  | chr16 | NR_027264,NM_001145667,NM_012201,NR_027265,NM_001145666 | 52.13 |
| CAMK1D | chr10 | NM_020397,NM_153498 | 52.12 |
| ORMDL3 | chr17 | NM_139280 | 52.12 |
| MAPRE2 | chr18 | NM_014268,NR_026570 | 52.11 |
| ELMSAN1 | chr14 | NM_194278 | 52.11 |
| AVEN | chr15 | NM_020371 | 52.1 |
| SOWAHB | chr4 | NM_001029870 | 52.09 |
| MT1G | chr16 | NM_005950,NM_005951 | 52.09 |
| RCBTB1 | chr13 | NM_018191 | 52.09 |
| UBN2 | chr7 | NM_173569 | 52.09 |
| FBXO15 | chr18 | NM_152676,NM_001142958,NM_014177 | 52.08 |
| TCERG1L | chr10 | NM_174937 | 52.08 |
| PAF1 | chr19 | NM_019088,NM_017592 | 52.07 |
| CADPS | chr3 | NM_183394,NM_183393,NM_003716 | 52.07 |
| FAM96A | chr15 | NM_001014812,NM_032231 | 52.06 |
| AMN1 | chr12 | NM_001113402,NR_004854 | 52.05 |
| ICAM4 | chr19 | NM_001544,NM_001039132,NM_022377,NM_003259 | 52.05 |
| PTRH2 | chr17 | NM_016077,NM_030938 | 52.05 |
| DPP10 | chr2 | NM_020868 | 52.05 |
| FAM92A1 | chr8 | NM_145269 | 52.05 |
| SATB1 | chr3 | NM_002971 | 52.04 |
| LAMP5 | chr20 | NM_012261 | 52.04 |
| GOLGA7B | chr10 | NM_001010917 | 52.03 |
| BBC3 | chr19 | NM_001127242,NM_014417,NM_001127241,NM_001127240 | 52.03 |
| SYP | chrX | NM_003179 | 52.03 |
| ZNF646 | chr16 | NM_014699,NM_024706,NM_001172668 | 52.03 |
| WNT3 | chr17 | NM_030753 | 52.03 |
| HNRNPUL1 | chr19 | NM_007040 | 52.02 |
| FOXO3 | chr6 | NM_201559 | 52.01 |
| SLC12A2 | chr5 | NM_001046,NR_015360 | 52 |
| TRIM2 | chr4 | NM_001130067 | 51.99 |
| FNDC3B | chr3 | NM_001135095,NM_022763 | 51.99 |
| SYNC | chr1 | NM_030786,NM_001161708 | 51.99 |
| DMXL2 | chr15 | NM_001174116,NM_001174117,NM_015263 | 51.98 |
| PYGB | chr20 | NM_002862 | 51.98 |
| TIPARP | chr3 | NM_015508,NR_027954 | 51.98 |
| USF1 | chr1 | NM_207005,NM_007122 | 51.98 |
| CSK | chr15 | NM_001127190,NM_004383 | 51.97 |
| RDX | chr11 | NM_002906 | 51.97 |
| SDC2 | chr8 | NM_002998 | 51.97 |
| CRY2 | chr11 | NM_021117,NM_001127457 | 51.97 |
| C6orf62 | chr6 | NM_030939 | 51.97 |
| PSAT1 | chr9 | NM_058179,NM_021154 | 51.97 |
| RUNX1 | chr21 | NM_001001890,NM_001122607 | 51.96 |
| NEUROD1 | chr2 | NM_002500 | 51.96 |
| SMG5 | chr1 | NM_015327,NM_032323,NR_026678 | 51.95 |
| NAPB | chr20 | NM_022080 | 51.95 |
| HAPLN1 | chr5 | NM_001884 | 51.94 |
| ZFPM2 | chr8 | NM_012082 | 51.93 |
| ZFHX4-AS1 | chr8 | NR_024360,NM_024721 | 51.93 |
| OARD1 | chr6 | NM_145063,NM_002505,NM_021705 | 51.92 |
| ESR1 | chr6 | NM_000125,NM_001122740 | 51.91 |
| PAX6 | chr11 | NM_001127612 | 51.91 |
| SYK | chr9 | NM_003177,NM_001135052,NR_024156,NM_001174168 | 51.9 |
| CCPG1 | chr15 | NM_004748,NM_020739 | 51.9 |
| CCDC25 | chr8 | NM_018246 | 51.9 |
| GPR150 | chr5 | NM_199243 | 51.89 |
| TSEN15 | chr1 | NM_001127394,NR_023349,NM_052965 | 51.88 |
| UQCRB | chr8 | NM_006294 | 51.88 |
| HECA | chr6 | NM_016217 | 51.88 |
| BAMBI | chr10 | NM_012342 | 51.87 |
| GLIS3 | chr9 | NM_001042413 | 51.86 |
| RASAL2 | chr1 | NM_004841 | 51.86 |
| DENND1C | chr19 | NM_024898 | 51.85 |
| ASXL3 | chr18 | NM_030632 | 51.85 |
| FAM110B | chr8 | NM_147189 | 51.85 |
| GPR158-AS1 | chr10 | NR_027333,NM_020752 | 51.85 |
| ITGA8 | chr10 | NM_003638 | 51.84 |
| ATP6V1C2 | chr2 | NM_144583,NM_001039362 | 51.84 |
| PHLDB1 | chr11 | NM_001144758,NM_015157,NM_001144759 | 51.83 |
| HCFC1 | chrX | NM_005334 | 51.83 |
| ARHGAP6 | chrX | NM_006125,NM_013427 | 51.83 |
| KIF26B | chr1 | NM_018012 | 51.83 |
| PSIP1 | chr9 | NM_021144,NM_033222,NM_001128217 | 51.83 |
| MEX3B | chr15 | NM_032246 | 51.83 |
| SRSF11 | chr1 | NM_004768,NM_017768 | 51.82 |
| DKK1 | chr10 | NM_012242 | 51.82 |
| PSMD11 | chr17 | NM_002815 | 51.81 |
| TRIO | chr5 | NM_007118 | 51.81 |
| UBA3 | chr3 | NM_003968,NM_198195 | 51.8 |
| MXRA5 | chrX | NM_015419 | 51.79 |
| NRXN2 | chr11 | NM_138734 | 51.79 |
| C21orf91 | chr21 | NM_017447,NM_001100421,NM_001100420 | 51.78 |
| UBTF | chr17 | NM_001076683 | 51.77 |
| NFE2L2 | chr2 | NM_001145413,NM_001145412 | 51.77 |
| FAM24B | chr10 | NM_152644,NR_027282 | 51.77 |
| CD59 | chr11 | NM_203330,NM_203329,NM_000611,NM_203331 | 51.77 |
| C17orf107 | chr17 | NM_001145536 | 51.76 |
| CCNG2 | chr4 | NM_004354 | 51.76 |
| TAF4B | chr18 | NM_005640 | 51.76 |
| SMC2 | chr9 | NM_006444,NM_001042551,NM_001042550 | 51.76 |
| LINC00476 | chr9 | NR_023390,NR_023389,NM_001010895 | 51.75 |
| CXCR4 | chr2 | NM_003467,NM_001008540 | 51.74 |
| SLC1A4 | chr2 | NM_003038,NM_001135581 | 51.73 |
| PDCD4 | chr10 | NM_014456,NR_026932,NM_145341 | 51.73 |
| SMPD4 | chr2 | NM_017751,NR_033230,NM_001171083,NM_025029,NR_033231,NR_033232,NM_017951,NM_001171084 | 51.73 |
| TEX2 | chr17 | NM_018469 | 51.73 |
| NOG | chr17 | NM_005450 | 51.72 |
| SERPINB6 | chr6 | NM_004568 | 51.71 |
| PTCH1 | chr9 | NM_001083607,NM_001083606 | 51.7 |
| BARHL1 | chr9 | NM_020064 | 51.7 |
| ARL6 | chr3 | NM_177976,NM_032146 | 51.7 |
| CCDC59 | chr12 | NR_033192,NM_032230,NM_014167 | 51.7 |
| RCCD1 | chr15 | NM_001017919,NM_033544 | 51.69 |
| DSE | chr6 | NM_013352 | 51.68 |
| BTN2A3P | chr6 | NR_027795 | 51.68 |
| CITED1 | chrX | NM_001144886,NM_004143,NM_001144885,NM_001144887 | 51.67 |
| FUT10 | chr8 | NM_032664 | 51.67 |
| PLGRKT | chr9 | NM_018465 | 51.66 |
| AGPAT1 | chr6 | NM_032741,NM_006411 | 51.66 |
| SLC16A14 | chr2 | NM_152527 | 51.66 |
| RCOR3 | chr1 | NM_001136223,NM_001136225,NM_001136224,NM_018254 | 51.65 |
| ZBED1 | chrX | NM_001171136,NM_145177,NM_004729 | 51.65 |
| AXIN2 | chr17 | NM_004655 | 51.65 |
| PELI2 | chr14 | NM_021255 | 51.65 |
| DHRSX | chrY | NM_145177,NM_004729,NM_001171136 | 51.65 |
| CXorf40A | chrX | NM_001171909,NM_001171908,NM_001171907,NM_178124 | 51.64 |
| DNAJC1 | chr10 | NM_022365 | 51.64 |
| CDKN2C | chr1 | NM_078626 | 51.64 |
| RPL30 | chr8 | NM_000989 | 51.63 |
| MARK2 | chr11 | NM_001163297,NM_001039469,NM_001163296,NM_004954 | 51.63 |
| PRDX4 | chrX | NM_006406 | 51.63 |
| HIST1H2BB | chr6 | NM_021062,NM_003531 | 51.63 |
| TRNP1 | chr1 | NM_001013642 | 51.62 |
| DPYSL3 | chr5 | NM_001387 | 51.62 |
| HIST1H3H | chr6 | NM_003536 | 51.62 |
| NAA15 | chr4 | NM_057175 | 51.62 |
| RBM33 | chr7 | NM_053043 | 51.61 |
| TMEM237 | chr2 | NM_001044385,NM_152388 | 51.6 |
| TENM4 | chr11 | NM_001098816 | 51.6 |
| PHLDB2 | chr3 | NM_001134439,NM_001134438,NM_145753 | 51.6 |
| FOXP4 | chr6 | NM_138457,NM_001012426,NM_001012427 | 51.6 |
| TLCD1 | chr17 | NM_138463,NM_178170,NM_001160407 | 51.6 |
| LRCH1 | chr13 | NM_015116,NM_001164213,NM_001164211 | 51.59 |
| RGMA | chr15 | NM_020211,NM_001166287 | 51.59 |
| HID1 | chr17 | NM_030630 | 51.59 |
| CNOT6L | chr4 | NM_144571 | 51.59 |
| ATP9B | chr18 | NM_198531 | 51.59 |
| CCDC66 | chr3 | NM_001141947,NR_024460,NM_001012506 | 51.58 |
| HACL1 | chr3 | NM_012260,NM_000060 | 51.58 |
| CCKBR | chr11 | NM_176875 | 51.58 |
| CDKN1C | chr11 | NM_001122631,NM_001122630,NM_000076 | 51.57 |
| PAFAH1B2 | chr11 | NM_002572 | 51.57 |
| TMCC2 | chr1 | NM_014858 | 51.57 |
| ELMOD1 | chr11 | NM_018712,NM_001130037,NR_028328 | 51.57 |
| GAS5 | chr1 | NR_002578,NR_002579,NM_032522,NM_001122770 | 51.57 |
| UROS | chr10 | NM_000375,NM_078468,NM_016567,NM_078469 | 51.56 |
| FHL1 | chrX | NM_001159700,NR_027621,NM_001449,NM_001159703,NM_001159702 | 51.56 |
| DYNC1LI2 | chr16 | NM_006141 | 51.56 |
| NGFRAP1 | chrX | NM_014380 | 51.56 |
| FAM184A | chr6 | NM_024581 | 51.56 |
| GJB2 | chr13 | NM_004004 | 51.55 |
| DLX3 | chr17 | NM_005220 | 51.55 |
| RNF6 | chr13 | NM_183044,NM_183043,NM_005977 | 51.55 |
| ZIC1 | chr3 | NM_003412,NM_032153,NM_001168379 | 51.54 |
| TFAP2B | chr6 | NM_003221 | 51.53 |
| CSTF2T | chr10 | NM_015235 | 51.53 |
| FEM1B | chr15 | NM_015322 | 51.53 |
| FAM129A | chr1 | NM_052966 | 51.53 |
| ITGB1 | chr10 | NM_133376,NM_002211 | 51.53 |
| PHKA2 | chrX | NM_000292 | 51.52 |
| NEDD4 | chr15 | NM_006154 | 51.52 |
| BSCL2 | chr11 | NM_001122955,NM_012202,NM_032667 | 51.51 |
| PLEKHJ1 | chr19 | NM_018049,NM_007165 | 51.51 |
| ARHGEF2 | chr1 | NM_004723 | 51.5 |
| CGRRF1 | chr14 | NM_006568 | 51.5 |
| SAP30L | chr5 | NM_024632,NR_024084,NM_001131063,NM_001131062 | 51.5 |
| PMP22 | chr17 | NM_153321 | 51.49 |
| GNB2L1 | chr5 | NR_002591,NM_006098 | 51.49 |
| SNORD95 | chr5 | NR_002591,NM_006098 | 51.49 |
| L2HGDH | chr14 | NM_024884,NM_001003803,NM_015684,NM_001003805 | 51.48 |
| ABCC8 | chr11 | NM_000352 | 51.47 |
| ZNF774 | chr15 | NM_001004309 | 51.47 |
| NRG3 | chr10 | NM_001010848,NM_001165972 | 51.47 |
| TOP2B | chr3 | NM_001068 | 51.47 |
| TRPC6 | chr11 | NM_004621 | 51.46 |
| LINC00338 | chr17 | NR_003013,NR_027058 | 51.46 |
| COA1 | chr7 | NM_018224 | 51.45 |
| DBX1 | chr11 | NM_001029865 | 51.44 |
| EML1 | chr14 | NM_004434,NM_001008707 | 51.43 |
| DHX29 | chr5 | NM_019030,NM_015360 | 51.43 |
| HOXB9 | chr17 | NM_024017 | 51.43 |
| TTC32 | chr2 | NM_001008237 | 51.41 |
| LHFPL2 | chr5 | NM_005779 | 51.41 |
| APH1B | chr15 | NM_031301,NM_001145646 | 51.41 |
| ADAMTS9 | chr3 | NM_182920 | 51.41 |
| CCDC88C | chr14 | NM_001080414 | 51.4 |
| SIN3A | chr15 | NM_001145357 | 51.4 |
| CCDC148 | chr2 | NM_001171637,NM_138803,NM_003628,NM_001005476 | 51.4 |
| FKBP2 | chr11 | NM_001135208,NM_004470,NM_057092 | 51.39 |
| EMP2 | chr16 | NM_001424 | 51.39 |
| SORCS3 | chr10 | NM_014978 | 51.39 |
| RNF43 | chr17 | NM_017763 | 51.39 |
| SPCS3 | chr4 | NM_021928 | 51.39 |
| SNHG5 | chr6 | NR_003044,NR_002743,NR_003038 | 51.39 |
| HNRNPK | chr9 | NM_002140,NM_031263,NM_031262,NM_024945 | 51.38 |
| CYB5R2 | chr11 | NM_016229 | 51.38 |
| MEIS3 | chr19 | NM_001009813,NM_020160 | 51.37 |
| PTPN13 | chr4 | NM_006264,NM_080683,NM_080684,NM_080685 | 51.37 |
| PSMB3 | chr17 | NM_002795 | 51.36 |
| PPIL1 | chr6 | NM_016059 | 51.36 |
| PARD3 | chr10 | NM_019619 | 51.36 |
| CHRNA7 | chr15 | NM_000746 | 51.35 |
| CASC15 | chr6 | NR_015410 | 51.35 |
| CSDE1 | chr1 | NM_007158,NM_001007553,NM_001130523 | 51.34 |
| HIVEP2 | chr6 | NM_006734 | 51.33 |
| ADRA1A | chr8 | NM_033302,NM_000680,NM_033304,NM_033303 | 51.33 |
| MIR548Q | chr9 | NR_031752 | 51.33 |
| NPY2R | chr4 | NM_000910 | 51.32 |
| PTMS | chr12 | NM_002824 | 51.31 |
| FBXO36 | chr2 | NM_174899,NM_004238 | 51.31 |
| NEK5 | chr13 | NM_199289 | 51.31 |
| IKZF2 | chr2 | NM_001079526,NM_016260 | 51.3 |
| SPAG9 | chr17 | NM_001130528,NM_003971 | 51.3 |
| GOLIM4 | chr3 | NM_014498 | 51.3 |
| URB1 | chr21 | NM_014825,NR_026845 | 51.3 |
| ZNF778 | chr16 | NM_182531 | 51.3 |
| IFNGR1 | chr6 | NM_000416 | 51.29 |
| NIPSNAP3A | chr9 | NM_015469 | 51.29 |
| SUDS3 | chr12 | NM_022491 | 51.29 |
| CXorf36 | chrX | NM_176819,NM_024689 | 51.29 |
| MYADM | chr19 | NM_001020820,NM_138373,NM_001020819 | 51.28 |
| CLTC | chr17 | NM_004859 | 51.28 |
| DDX20 | chr1 | NM_007204,NM_198926 | 51.28 |
| PIEZO2 | chr18 | NM_022068 | 51.28 |
| DCAF11 | chr14 | NM_025230,NM_001163484,NR_028099,NM_181357,NR_028100 | 51.28 |
| SNX1 | chr15 | NM_148955,NM_152826,NM_003099 | 51.28 |
| ORAOV1 | chr11 | NM_153451 | 51.28 |
| BCAR1 | chr16 | NM_001170714,NM_001170718,NM_001170720 | 51.27 |
| FGF2 | chr4 | NM_002006 | 51.27 |
| PIK3CD | chr1 | NM_005026 | 51.27 |
| NXPH2 | chr2 | NM_007226 | 51.27 |
| GFRA1 | chr10 | NM_145793,NM_001145453,NM_005264 | 51.27 |
| SNORD42B | chr17 | NR_000013,NR_024575,NM_001142625,NM_001144943,NR_024579,NM_001142624,NM_000984 | 51.27 |
| ATMIN | chr16 | NM_015251 | 51.26 |
| ZFX | chrX | NM_003410 | 51.25 |
| SH2B3 | chr12 | NM_005475 | 51.25 |
| SIX1 | chr14 | NM_005982 | 51.25 |
| FZD3 | chr8 | NM_017412 | 51.25 |
| UBE2Q1 | chr1 | NM_017582 | 51.25 |
| SLC7A2 | chr8 | NM_001008539 | 51.24 |
| ADM | chr11 | NM_001124 | 51.24 |
| MX1 | chr21 | NM_002462 | 51.24 |
| POU3F2 | chr6 | NM_005604 | 51.24 |
| ZFYVE1 | chr14 | NM_021260 | 51.24 |
| FBXL14 | chr12 | NM_152441 | 51.24 |
| KLHL5 | chr4 | NM_001007075,NM_001171654 | 51.23 |
| CD2AP | chr6 | NM_012120 | 51.23 |
| GNL3 | chr3 | NM_014366,NM_018313,NM_206826,NM_206825 | 51.23 |
| ARID2 | chr12 | NM_152641,NR_028408 | 51.23 |
| IQSEC2 | chrX | NM_015075,NR_024449 | 51.22 |
| SESTD1 | chr2 | NM_178123 | 51.22 |
| MARCKS | chr6 | NM_002356 | 51.21 |
| PHF19 | chr9 | NM_001009936,NM_015651 | 51.2 |
| TAZ | chrX | NM_000116,NR_024048,NM_181311,NM_181312,NM_181313,NM_001009934,NM_001009933,NM_001009932 | 51.19 |
| OCIAD1 | chr4 | NM_001079840,NM_017830,NM_001079841,NM_001079842,NM_001079839,NM_001168254 | 51.19 |
| DGKZ | chr11 | NM_003646 | 51.19 |
| SATB2 | chr2 | NM_001172517 | 51.18 |
| LMX1B | chr9 | NM_002316,NM_001174147,NM_001174146 | 51.18 |
| MAPK4 | chr18 | NM_002747 | 51.18 |
| FAM19A2 | chr12 | NM_178539 | 51.18 |
| CYP4V2 | chr4 | NM_207352 | 51.18 |
| UNC13A | chr19 | NM_001080421 | 51.17 |
| TCEAL8 | chrX | NM_153333,NM_001006684 | 51.17 |
| RIC3 | chr11 | NM_024557,NM_001135109 | 51.16 |
| SIRT2 | chr19 | NM_030593,NM_002503,NM_012237,NM_001001716 | 51.16 |
| SHOX | chrX | NM_000451,NM_006883 | 51.15 |
| GATA3 | chr10 | NM_002051,NM_001002295,NR_024255,NR_024256 | 51.15 |
| SLC7A1 | chr13 | NM_003045 | 51.15 |
| SHOX | chrY | NM_006883,NM_000451 | 51.15 |
| GPANK1 | chr6 | NM_033177,NM_001320 | 51.15 |
| BDNF | chr11 | NM_001143807 | 51.14 |
| YWHAZ | chr8 | NM_003406,NM_001135699,NM_145690 | 51.14 |
| PTCD2 | chr5 | NM_024754,NM_015084 | 51.14 |
| HAS2 | chr8 | NM_005328,NR_002835 | 51.13 |
| GPC6 | chr13 | NM_005708 | 51.13 |
| PPM1A | chr14 | NM_021003,NM_177951 | 51.13 |
| ST3GAL1 | chr8 | NM_173344,NM_003033 | 51.13 |
| ZCCHC16 | chrX | NM_001004308,NM_012471 | 51.12 |
| TC2N | chr14 | NM_001128595,NM_152332 | 51.12 |
| FAM189A1 | chr15 | NM_015307 | 51.12 |
| UFM1 | chr13 | NM_016617 | 51.12 |
| PCDH17 | chr13 | NM_001040429 | 51.11 |
| ISLR2 | chr15 | NM_001130136,NR_027073,NM_001130137,NM_001130138 | 51.11 |
| TNFRSF8 | chr1 | NM_001243 | 51.11 |
| TMEM50B | chr21 | NM_006134 | 51.11 |
| PTCH1 | chr9 | NM_000264,NM_001083605,NM_001083607,NM_001083606,NM_001083604 | 51.1 |
| SPIRE1 | chr18 | NM_001128627,NM_001128626,NM_020148 | 51.1 |
| MAF | chr16 | NM_005360,NM_001031804 | 51.1 |
| DNER | chr2 | NM_139072 | 51.1 |
| OPRK1 | chr8 | NM_000912 | 51.09 |
| DBP | chr19 | NM_001352 | 51.08 |
| ZMYM3 | chrX | NM_005096,NM_201599,NM_001171163 | 51.08 |
| ZBTB5 | chr9 | NM_014872 | 51.08 |
| BCOR | chrX | NM_001123383,NM_001123384 | 51.07 |
| IFRD1 | chr7 | NM_001550 | 51.06 |
| KL | chr13 | NM_004795 | 51.06 |
| FXYD7 | chr19 | NM_022006,NM_005031 | 51.06 |
| TP53RK | chr20 | NM_033550 | 51.06 |
| ZMIZ1-AS1 | chr10 | NR_024431,NR_015429,NR_024429,NM_020338 | 51.06 |
| GRM7 | chr3 | NM_000844,NM_181874 | 51.05 |
| SLC25A29 | chr14 | NM_001039355 | 51.05 |
| IDH2 | chr15 | NM_002168 | 51.05 |
| ANKRD16 | chr10 | NM_001009941,NM_019046,NM_001009943,NM_178150 | 51.04 |
| MERTK | chr2 | NM_006343 | 51.04 |
| UBC | chr12 | NM_021009 | 51.04 |
| PCDHGA11 | chr5 | NM_032092,NM_018914,NM_032091 | 51.04 |
| ZDHHC14 | chr6 | NM_153746,NM_024630 | 51.04 |
| PTPN9 | chr15 | NM_002833 | 51.03 |
| NFIC | chr19 | NM_005597 | 51.03 |
| HOXD9 | chr2 | NM_014213 | 51.03 |
| ZNF280D | chr15 | NM_001002843,NM_017661,NM_001002844 | 51.02 |
| CABP1 | chr12 | NM_001033677 | 51.02 |
| BZW2 | chr7 | NM_014038,NM_001159767,NR_027624,NM_020319 | 51.02 |
| POU3F1 | chr1 | NM_002699 | 51.01 |
| DACH1 | chr13 | NM_004392,NM_080760,NM_080759 | 51.01 |
| KLF13 | chr15 | NM_015995 | 51.01 |
| TMEM206 | chr1 | NM_018252 | 51.01 |
| KLHL13 | chrX | NM_001168301,NM_001168303,NM_001168302 | 51 |
| FJX1 | chr11 | NM_014344 | 51 |
| IFT140 | chr16 | NM_014714,NM_020825 | 51 |
| CCDC146 | chr7 | NM_020879 | 51 |
| ZNF763 | chr19 | NM_001012753 | 50.99 |
| GGA3 | chr17 | NM_001172703,NR_033345,NM_138619,NM_015971,NM_014001,NM_001172704 | 50.99 |
| STRN | chr2 | NM_003162 | 50.99 |
| RPRD2 | chr1 | NM_015203 | 50.99 |
| BTBD9 | chr6 | NM_052893,NM_001099272 | 50.99 |
| SPATA5 | chr4 | NM_145207,NM_007083,NM_198041 | 50.99 |
| MAP3K11 | chr11 | NM_002419,NM_032223 | 50.98 |
| OLIG1 | chr21 | NM_138983 | 50.98 |
| TSPAN14 | chr10 | NM_001128309,NM_030927 | 50.97 |
| EGR2 | chr10 | NM_001136179,NM_000399,NM_001136177,NM_001136178 | 50.97 |
| BTG1 | chr12 | NM_001731 | 50.97 |
| PRKACA | chr19 | NM_002730 | 50.97 |
| TGIF1 | chr18 | NM_170695,NM_003244,NM_173210,NM_173211 | 50.97 |
| MGAT1 | chr5 | NM_001114618,NM_001114617 | 50.96 |
| CCT5 | chr5 | NM_012073,NM_199133 | 50.96 |
| RDH10 | chr8 | NM_172037 | 50.96 |
| FFAR4 | chr10 | NM_181745 | 50.96 |
| CRMP1 | chr4 | NM_001313 | 50.95 |
| EFS | chr14 | NM_032459,NM_005864 | 50.95 |
| SPSB4 | chr3 | NM_080862 | 50.95 |
| PTPRF | chr1 | NM_130440,NM_002840 | 50.94 |
| MSX2 | chr5 | NM_002449 | 50.93 |
| GADD45B | chr19 | NM_015675 | 50.93 |
| METTL9 | chr16 | NM_016025,NM_001077180 | 50.93 |
| LRRK2 | chr12 | NM_198578 | 50.93 |
| GNAS-AS1 | chr20 | NR_002785,NM_080425,NM_001077490 | 50.93 |
| SAP30 | chr4 | NM_003864 | 50.92 |
| HDGF | chr1 | NM_004494,NM_001126051,NM_001126050 | 50.91 |
| SUN2 | chr22 | NM_015374 | 50.91 |
| PCDHGB3 | chr5 | NM_018924,NM_032097,NM_018919,NM_032086 | 50.91 |
| VGLL2 | chr6 | NM_153453,NM_182645 | 50.91 |
| RFX4 | chr12 | NM_213594 | 50.91 |
| SNHG7 | chr9 | NR_024543,NR_002975,NR_002958,NR_024542,NR_003672 | 50.91 |
| LRP4 | chr11 | NM_002334 | 50.9 |
| KDM1A | chr1 | NM_015013,NM_001009999 | 50.9 |
| EVA1C | chr21 | NM_058187 | 50.9 |
| RAI14 | chr5 | NM_001145520,NM_001145522,NM_015577 | 50.89 |
| ADRBK1 | chr11 | NM_001619 | 50.89 |
| BCL3 | chr19 | NM_005178 | 50.89 |
| LRRC1 | chr6 | NM_018214 | 50.89 |
| CADM4 | chr19 | NM_145296 | 50.89 |
| TMEM255A | chrX | NM_001104544,NM_001104545,NM_017938 | 50.88 |
| TEAD1 | chr11 | NM_021961 | 50.88 |
| LRP11 | chr6 | NM_032832 | 50.88 |
| MIR1915 | chr10 | NR_031736,NM_001010911 | 50.88 |
| LAMTOR2 | chr1 | NM_014017,NM_020131,NM_001145264 | 50.87 |
| BBC3 | chr19 | NM_014417 | 50.87 |
| FAM220A | chr7 | NM_001037163 | 50.86 |
| AP3M2 | chr8 | NM_001134296,NM_006803 | 50.86 |
| STIM2 | chr4 | NM_001169117,NM_001169118,NM_020860 | 50.86 |
| TRAPPC10 | chr21 | NM_003274 | 50.86 |
| PHC1 | chr12 | NM_004426 | 50.86 |
| ZNF607 | chr19 | NM_032689,NM_001172677 | 50.86 |
| HAR1A | chr20 | NR_003244 | 50.86 |
| CTNNB1 | chr3 | NM_001098209,NM_001098210,NM_001904 | 50.85 |
| KLHL15 | chrX | NM_030624 | 50.85 |
| PKD2 | chr4 | NM_000297 | 50.84 |
| KLF7 | chr2 | NM_003709 | 50.84 |
| USP11 | chrX | NM_004651 | 50.84 |
| NGFR | chr17 | NM_002507 | 50.83 |
| MYBPC2 | chr19 | NM_004533 | 50.83 |
| MTMR7 | chr8 | NM_004686 | 50.83 |
| CAP2 | chr6 | NM_006366 | 50.83 |
| RNF180 | chr5 | NM_178532,NM_001113561 | 50.83 |
| RFPL2 | chr22 | NM_001098527,NM_001159546,NM_001159545 | 50.82 |
| ABLIM2 | chr4 | NM_001130086,NM_001130085,NM_032432,NM_001130087,NM_001130083,NM_001130088,NM_001130084 | 50.82 |
| CDK17 | chr12 | NM_002595,NM_001170464 | 50.82 |
| GLIPR1L1 | chr12 | NM_152779 | 50.82 |
| MUM1 | chr19 | NM_032853,NR_024247 | 50.81 |
| NTRK3 | chr15 | NM_001012338,NM_002530,NM_001007156 | 50.8 |
| DUSP6 | chr12 | NM_001946,NM_022652 | 50.8 |
| UBXN4 | chr2 | NM_014607 | 50.8 |
| KLHL24 | chr3 | NM_017644 | 50.8 |
| CCDC181 | chr1 | NM_021179 | 50.8 |
| HNF1B | chr17 | NM_000458,NM_001165923 | 50.79 |
| POP4 | chr19 | NM_006627,NR_027368 | 50.79 |
|  | chr3 | NM_016275 | 50.79 |
| NCAPG2 | chr7 | NM_017760 | 50.79 |
| GSX1 | chr13 | NM_145657 | 50.79 |
| HSP90AA1 | chr14 | NM_005348 | 50.78 |
| NDUFAF2 | chr5 | NM_174889,NM_000082 | 50.78 |
| EWSR1 | chr22 | NM_001163287,NM_012265,NM_005243,NM_001163285,NM_001163286,NM_013986 | 50.77 |
| GYS1 | chr19 | NM_002103,NM_001161587,NR_027763,NM_006666 | 50.77 |
| PTPN2 | chr18 | NM_002828,NM_080422,NM_080423 | 50.77 |
| SLCO3A1 | chr15 | NM_013272,NM_001145044 | 50.77 |
| COLGALT2 | chr1 | NM_015101 | 50.77 |
| TRMT12 | chr8 | NM_017956 | 50.77 |
| TSC22D1 | chr13 | NM_183422 | 50.77 |
| LINC00271 | chr6 | NR_026805,NM_001134831,NM_001134832,NM_001134830,NM_017651 | 50.77 |
| RSPO1 | chr1 | NM_001038633 | 50.76 |
| ADCY8 | chr8 | NM_001115 | 50.76 |
| CDS1 | chr4 | NM_001263 | 50.76 |
| CDH8 | chr16 | NM_001796 | 50.76 |
| CKS2 | chr9 | NM_001827 | 50.76 |
| PJA2 | chr5 | NM_014819 | 50.76 |
| PRR18 | chr6 | NM_175922 | 50.76 |
| ZNF580 | chr19 | NM_001163423,NM_016202,NM_016535 | 50.75 |
| KRTAP5-AS1 | chr11 | NR_021489,NM_004420 | 50.75 |
| PAX9 | chr14 | NM_006194 | 50.74 |
| RBP4 | chr10 | NM_006744 | 50.74 |
| BRCA1 | chr17 | NM_007297,NM_007298,NR_027676,NR_003108,NM_007299,NM_007300,NM_007294 | 50.74 |
| DGAT2 | chr11 | NM_032564 | 50.74 |
| SLAIN1 | chr13 | NM_001040153 | 50.73 |
| HES7 | chr17 | NM_001165967,NM_032580 | 50.73 |
| EPN1 | chr19 | NM_013333,NM_001130072,NM_001130071 | 50.73 |
| FBXL19-AS1 | chr16 | NR_024348 | 50.73 |
| MIR17HG | chr13 | NR_027349,NR_029492,NR_029487,NR_027350,NR_029489,NR_029488 | 50.73 |
| ALDH1L1 | chr3 | NM_012190 | 50.72 |
| STK39 | chr2 | NM_013233 | 50.72 |
| GATAD1 | chr7 | NM_021167 | 50.72 |
| FAM188A | chr10 | NM_024948 | 50.71 |
| PITPNM3 | chr17 | NM_031220,NM_001165966 | 50.71 |
| TSC22D2 | chr3 | NM_014779 | 50.7 |
| SNTG1 | chr8 | NM_018967 | 50.7 |
| PITHD1 | chr1 | NM_020362 | 50.7 |
| TARBP2 | chr12 | NM_004178,NM_006301,NM_134323 | 50.69 |
| COL12A1 | chr6 | NM_004370,NM_080645 | 50.69 |
| NUAK1 | chr12 | NM_014840 | 50.69 |
| VPS54 | chr2 | NM_016516,NM_001005739 | 50.69 |
| MIR132 | chr17 | NR_029674,NR_029625 | 50.69 |
| TMEM151A | chr11 | NM_153266 | 50.68 |
| FOXM1 | chr12 | NM_202002,NM_202003,NM_021953,NR_027365,NR_027364,NR_027363 | 50.68 |
| SP5 | chr2 | NM_001003845 | 50.67 |
| CELF2 | chr10 | NM_001083591,NM_001025076 | 50.67 |
| ZNF222 | chr19 | NM_001129996,NM_013360 | 50.67 |
| RASAL2 | chr1 | NM_170692,NR_027982 | 50.67 |
| FLOT1 | chr6 | NM_005803,NM_003897 | 50.66 |
| WDFY3 | chr4 | NM_014991,NR_015359 | 50.66 |
| DLX5 | chr7 | NM_005221 | 50.65 |
| ARNT2 | chr15 | NM_014862 | 50.65 |
| YAE1D1 | chr7 | NM_020192 | 50.65 |
| CFLAR | chr2 | NM_003879,NM_001127184 | 50.64 |
| RBL2 | chr16 | NM_005611 | 50.64 |
|  | chr9 | NR_015375,NM_003383,NM_001018056 | 50.64 |
| EEF1B2 | chr2 | NM_001037663,NM_005006,NM_021121,NM_001959 | 50.63 |
| SMIM20 | chr4 | NM_001145432 | 50.63 |
| HSPH1 | chr13 | NM_006644 | 50.63 |
| KMT2E | chr7 | NM_018682,NM_182931,NR_024586 | 50.63 |
| DCBLD2 | chr3 | NM_080927 | 50.63 |
| ESRRG | chr1 | NM_001438,NR_024099 | 50.62 |
| KIAA0232 | chr4 | NM_014743,NM_001100590 | 50.62 |
| ABHD5 | chr3 | NM_016006 | 50.62 |
| C14orf166 | chr14 | NM_016039 | 50.62 |
| RARG | chr12 | NM_000966 | 50.61 |
| DHTKD1 | chr10 | NM_018706 | 50.61 |
| NPAS4 | chr11 | NM_178864 | 50.61 |
| C9orf47 | chr9 | NM_001001938,NM_001142413 | 50.59 |
| GPC4 | chrX | NM_001448 | 50.59 |
| JARID2 | chr6 | NM_004973 | 50.59 |
| RIMKLB | chr12 | NM_020734 | 50.59 |
| HOXA7 | chr7 | NM_006896 | 50.58 |
| VEGFC | chr4 | NM_005429 | 50.57 |
| REST | chr4 | NM_005612 | 50.57 |
| TMOD2 | chr15 | NM_014548,NM_001143917,NM_001142885 | 50.57 |
| KHDRBS2 | chr6 | NM_152688 | 50.57 |
| MYCBP2 | chr13 | NM_015057 | 50.56 |
| ELOVL5 | chr6 | NM_021814 | 50.56 |
| GABRB3 | chr15 | NM_021912,NM_000814 | 50.56 |
| NPTN | chr15 | NM_001161364,NM_001161363,NM_012428,NM_017455 | 50.55 |
| EMX2 | chr10 | NM_001165924,NR_002791,NM_004098 | 50.55 |
| C1orf116 | chr1 | NM_023938,NM_001083924 | 50.55 |
| TMEM65 | chr8 | NM_194291 | 50.55 |
| ZFP62 | chr5 | NM_152283,NM_001172638 | 50.54 |
| NME9 | chr3 | NM_178130 | 50.54 |
| GATA4 | chr8 | NM_002052 | 50.53 |
| NKX2-8 | chr14 | NM_014360 | 50.53 |
| SLC32A1 | chr20 | NM_080552 | 50.53 |
| SCGB1D4 | chr11 | NM_206998 | 50.53 |
| HTR1E | chr6 | NM_000865 | 50.52 |
| BMF | chr15 | NM_001003943,NM_001003942 | 50.52 |
| CDV3 | chr3 | NM_001134423,NM_001134422,NM_017548 | 50.52 |
| DDX18 | chr2 | NM_006773 | 50.52 |
| XPNPEP3 | chr22 | NM_022098,NM_003932 | 50.52 |
| C18orf21 | chr18 | NM_031446 | 50.52 |
| PANK1 | chr10 | NM_148977,NM_148978,NM_138316 | 50.51 |
| EEA1 | chr12 | NM_003566 | 50.5 |
| CRLS1 | chr20 | NM_019095,NM_001127458 | 50.5 |
| ATE1 | chr10 | NM_001001976,NM_007041 | 50.49 |
| PBX3 | chr9 | NM_006195,NR_024122,NM_001134778,NR_024123 | 50.49 |
| FLVCR2 | chr14 | NM_017791 | 50.49 |
| BRINP2 | chr1 | NM_021165 | 50.49 |
| FIBIN | chr11 | NM_203371 | 50.49 |
| C18orf32 | chr18 | NM_001035005,NR_031720,NR_002573 | 50.48 |
| PHF11 | chr13 | NM_001040443,NM_001040444 | 50.48 |
| MCC | chr5 | NM_002387 | 50.48 |
| IRX5 | chr16 | NM_005853 | 50.48 |
| HECW1 | chr7 | NM_015052 | 50.48 |
| HOXB8 | chr17 | NM_024016 | 50.48 |
| SNX30 | chr9 | NM_001012994 | 50.47 |
| SGCZ | chr8 | NM_139167 | 50.47 |
| LRP2 | chr2 | NM_004525 | 50.46 |
| ADAMTS1 | chr21 | NM_006988 | 50.46 |
| MAP7D2 | chrX | NM_152780,NM_001168466,NM_001168465,NM_001168467 | 50.46 |
| PALD1 | chr10 | NM_014431 | 50.45 |
| DDN | chr12 | NM_015086 | 50.45 |
| MYO1D | chr17 | NM_015194 | 50.45 |
| DTX3 | chr12 | NM_178502 | 50.45 |
| VPS13C | chr15 | NM_001018088,NM_020821,NM_017684,NM_018080 | 50.44 |
| MTHFD2L | chr4 | NM_001144978 | 50.44 |
| SCMH1 | chr1 | NM_001172218,NM_012236,NM_001172220,NM_001172219 | 50.44 |
| MAN2A1 | chr5 | NM_002372 | 50.44 |
| SEMA3F | chr3 | NM_004186 | 50.44 |
| ONECUT2 | chr18 | NM_004852 | 50.44 |
| PFN1 | chr17 | NM_005022,NM_001976,NM_053013 | 50.44 |
| NRGN | chr11 | NM_006176,NM_001126181 | 50.44 |
| GAREM | chr18 | NM_022751 | 50.44 |
| TBK1 | chr12 | NM_013254 | 50.43 |
| JDP2 | chr14 | NM_130469,NM_001135048,NM_001135047 | 50.43 |
| KCTD1 | chr18 | NM_001142730,NM_001136205 | 50.42 |
| PSMD1 | chr2 | NM_002807 | 50.42 |
| POLG2 | chr17 | NM_007215 | 50.42 |
| ZC2HC1A | chr8 | NM_016010 | 50.42 |
| FNIP2 | chr4 | NM_020840 | 50.42 |
| FNDC3A | chr13 | NM_001079673 | 50.41 |
| HOMER3 | chr19 | NM_001145721,NR_027297,NM_001145724,NM_001145722,NM_004838 | 50.41 |
| HMGXB3 | chr5 | NM_014983,NM_030953 | 50.41 |
| ADAMTS6 | chr5 | NM_197941 | 50.41 |
| PRDM2 | chr1 | NM_001007257 | 50.4 |
| ACTR3C | chr7 | NM_001164459 | 50.4 |
| ERBB3 | chr12 | NM_001982,NM_001005915 | 50.4 |
| UBE2D1 | chr10 | NM_003338 | 50.4 |
| ADAMTS2 | chr5 | NM_014244,NM_021599 | 50.4 |
| UPRT | chrX | NM_145052,NR_030774 | 50.4 |
| FBXL5 | chr4 | NM_012161,NM_033535 | 50.39 |
| MAML1 | chr5 | NM_014757 | 50.39 |
| CHPF2 | chr7 | NM_019015 | 50.39 |
| WBSCR17 | chr7 | NM_022479 | 50.39 |
| SERINC2 | chr1 | NM_178865 | 50.39 |
| TSC22D3 | chrX | NM_001015881,NM_004089 | 50.38 |
| PTPRG | chr3 | NM_002841 | 50.38 |
| LRRC32 | chr11 | NM_005512,NM_001128922 | 50.37 |
| CCT3 | chr1 | NM_005998,NM_001008883,NM_144627,NM_001008800 | 50.37 |
| ELMO1 | chr7 | NM_130442 | 50.37 |
| GABRA5 | chr15 | NM_001165037,NM_000810 | 50.36 |
| DLX4 | chr17 | NM_001934 | 50.36 |
| MSC | chr8 | NM_005098 | 50.36 |
| HOTAIR | chr12 | NR_003716 | 50.36 |
| PTPN18 | chr2 | NM_001142370,NM_014369 | 50.35 |
| SLITRK2 | chrX | NM_001144006,NM_001144009,NM_001144010,NM_001144008 | 50.35 |
| CHIC2 | chr4 | NM_012110 | 50.35 |
| TRHDE | chr12 | NM_013381 | 50.35 |
| PCDHGB2 | chr5 | NM_018923,NM_032096 | 50.35 |
| GFRA1 | chr10 | NM_145793,NM_001145453 | 50.35 |
| NLGN4X | chrX | NM_181332,NM_020742 | 50.35 |
| CUX1 | chr7 | NM_001913,NM_181500 | 50.34 |
| TMEM159 | chr16 | NM_020422,NM_017539 | 50.33 |
| MRPL34 | chr19 | NM_023937 | 50.33 |
| STK24 | chr13 | NM_001032296 | 50.32 |
| RYR3 | chr15 | NM_001036 | 50.32 |
| BICC1 | chr10 | NM_001080512 | 50.31 |
| FYN | chr6 | NM_002037 | 50.31 |
| PRKX | chrX | NM_005044 | 50.31 |
| TNFAIP3 | chr6 | NM_006290 | 50.31 |
| SLC35C2 | chr20 | NM_015945,NM_173179,NM_173073 | 50.31 |
| TBC1D24 | chr16 | NM_020705 | 50.31 |
| EGLN3 | chr14 | NM_022073 | 50.31 |
| ABTB2 | chr11 | NM_145804 | 50.31 |
| EIF2B4 | chr2 | NM_172195,NM_015636,NM_001034116,NM_014748 | 50.31 |
| RBPMS | chr8 | NM_001008712,NM_006867,NM_001008711,NM_001008710 | 50.3 |
| HS3ST3A1 | chr17 | NM_006042 | 50.3 |
| JAGN1 | chr3 | NM_032492 | 50.3 |
| CERKL | chr2 | NM_001160277,NM_001030313,NM_001030312,NR_027690,NM_001030311,NM_201548,NR_027689 | 50.29 |
| TM9SF3 | chr10 | NM_020123 | 50.29 |
| ZBTB49 | chr4 | NM_145291,NM_001145725,NM_017816 | 50.29 |
| FAM115C | chr7 | NM_001130026 | 50.28 |
| GRIK2 | chr6 | NM_001166247,NM_021956,NM_175768 | 50.28 |
| YWHAZ | chr8 | NM_003406,NM_001135699,NM_001135701,NM_001135700,NM_145690 | 50.28 |
| DNAJC4 | chr11 | NM_005528 | 50.28 |
| PRTG | chr15 | NM_173814 | 50.28 |
| MAPK8IP2 | chr22 | NM_016431,NM_012324 | 50.27 |
| ZCCHC2 | chr18 | NM_017742 | 50.27 |
| ALCAM | chr3 | NM_001627 | 50.26 |
|  | chr17 | NR_029376,NM_001164637,NM_173627,NM_001164638 | 50.26 |
| MIR22HG | chr17 | NR_029494 | 50.26 |
| GBX2 | chr2 | NM_001485 | 50.25 |
| FRAT2 | chr10 | NM_012083 | 50.25 |
| MAGI2 | chr7 | NM_012301 | 50.25 |
| CCDC22 | chrX | NM_014008 | 50.25 |
| FEV | chr2 | NM_017521 | 50.25 |
| ALG13 | chrX | NM_018466,NR_033124,NM_001099922,NM_001039210,NR_033125,NR_033132,NR_033129,NM_001168385,NR_033134,NR_033128,NR_033127,NR_033131 | 50.25 |
| ZFYVE28 | chr4 | NM_020972,NM_001172657,NM_001172656,NM_001172658 | 50.25 |
| IRS4 | chrX | NM_003604 | 50.24 |
| TNK2 | chr3 | NM_005781 | 50.23 |
| SRD5A3 | chr4 | NM_024592 | 50.23 |
| IFNAR1 | chr21 | NM_000629 | 50.22 |
| DMTF1 | chr7 | NM_001142327,NM_001142326,NR_024549,NR_024550,NM_021145 | 50.22 |
| SLITRK2 | chrX | NM_001144005,NM_032539,NM_001144004,NM_001144003 | 50.22 |
| FLII | chr17 | NM_002018,NM_139162,NM_001144900 | 50.22 |
| PDGFC | chr4 | NM_016205 | 50.22 |
| ZRSR2 | chrX | NM_005089 | 50.21 |
| FOXN3 | chr14 | NM_005197 | 50.21 |
| ORC6 | chr16 | NM_014321,NM_018206 | 50.21 |
| CTPS2 | chrX | NM_175859,NM_001144002,NM_019857 | 50.21 |
| HOXA9 | chr7 | NR_029911 | 50.21 |
| HOXA10-AS | chr7 | NR_029911 | 50.21 |
| MIR196B | chr7 | NR_029911 | 50.21 |
| NDUFA5 | chr7 | NM_005000 | 50.2 |
| ARPC3 | chr12 | NM_005719 | 50.2 |
| MYO10 | chr5 | NM_012334 | 50.2 |
| ZNF219 | chr14 | NM_016423,NM_001146683,NM_001101672 | 50.2 |
| LRRK1 | chr15 | NM_024652 | 50.2 |
| FIZ1 | chr19 | NM_032836,NM_153219 | 50.2 |
| STRIP1 | chr1 | NM_033088 | 50.2 |
| ARID1B | chr6 | NM_020732,NM_175863,NM_017519 | 50.19 |
| KRTAP5-AS1 | chr11 | NR_021489 | 50.19 |
| DUSP4 | chr8 | NM_001394,NM_057158 | 50.18 |
| ICAM5 | chr19 | NM_003259 | 50.18 |
| UVRAG | chr11 | NM_003369 | 50.18 |
| ST7L | chr1 | NM_138727,NM_138729,NM_138728,NM_017744,NM_006135 | 50.18 |
| TMEM92 | chr17 | NM_153229,NM_001168215 | 50.18 |
| DNM2 | chr19 | NM_001005360,NM_001005361,NM_004945,NM_001005362,NR_030368 | 50.17 |
| HIP1 | chr7 | NM_005338 | 50.17 |
| PAXBP1 | chr21 | NM_013329,NR_027873,NR_024622,NM_016631,NR_024623 | 50.17 |
| GJA3 | chr13 | NM_021954 | 50.17 |
| MSH5 | chr6 | NM_025259,NM_002441,NM_172166,NM_172165 | 50.17 |
| GAPDH | chr12 | NM_002046 | 50.16 |
| UCHL1 | chr4 | NM_004181 | 50.16 |
| BCAS3 | chr17 | NM_001099432,NM_017679 | 50.15 |
| STAT6 | chr12 | NM_003153 | 50.15 |
| JAK2 | chr9 | NM_004972 | 50.15 |
| MYEF2 | chr15 | NM_016132 | 50.15 |
| WNK4 | chr17 | NM_032387 | 50.15 |
| SCNN1G | chr16 | NM_001039 | 50.14 |
| SPATS2L | chr2 | NM_001100424,NM_015535,NM_001100422,NM_001100423 | 50.14 |
| IRF4 | chr6 | NM_002460 | 50.14 |
| ZRANB2 | chr1 | NM_005455,NM_203350 | 50.14 |
| WDR11 | chr10 | NM_018117 | 50.14 |
| ZAR1 | chr4 | NM_175619 | 50.14 |
| CRY1 | chr12 | NM_004075 | 50.13 |
| KIAA0753 | chr17 | NM_014804,NM_032731 | 50.13 |
| NLGN3 | chrX | NM_181303,NM_018977,NM_001166660 | 50.13 |
| TRMT11 | chr6 | NM_001031712 | 50.12 |
| GYG1 | chr3 | NM_004130 | 50.12 |
| PIK3CA | chr3 | NM_006218 | 50.12 |
| HMGN2 | chr1 | NM_005517 | 50.11 |
| RPL7A | chr9 | NR_002447,NM_000972,NM_181491,NM_133640,NR_000017 | 50.11 |
|  | chr9 | NR_002447,NM_000972,NM_181491,NM_133640,NR_000017 | 50.11 |
| SNORD24 | chr9 | NR_002447,NM_000972,NM_181491,NM_133640,NR_000017 | 50.11 |
| HIST1H1B | chr6 | NM_005322 | 50.1 |
| SYBU | chr8 | NM_001099744,NM_001099743 | 50.09 |
| ATP2C1 | chr3 | NM_014382,NM_001001486,NM_001001487,NM_001001485 | 50.09 |
| RTTN | chr18 | NM_173630 | 50.09 |
| APC | chr5 | NM_001127511 | 50.08 |
| FDX1 | chr11 | NM_004109 | 50.08 |
| FBXL15 | chr10 | NM_024326 | 50.08 |
| FAM57A | chr17 | NM_024792 | 50.08 |
| HEXA | chr15 | NM_000520,NR_027262 | 50.07 |
| CTNND2 | chr5 | NM_001332 | 50.07 |
| FBXL7 | chr5 | NM_012304 | 50.07 |
| GPHN | chr14 | NM_020806,NM_001024218 | 50.07 |
| TMEM38A | chr19 | NM_024074,NM_024104 | 50.07 |
| SGMS1 | chr10 | NM_147156 | 50.07 |
| MIR124-2 | chr8 | NR_029669 | 50.07 |
| PROK2 | chr3 | NM_001126128,NM_021935 | 50.06 |
| RNASEH2B | chr13 | NM_001142279,NM_024570 | 50.06 |
| RBBP7 | chrX | NM_002893 | 50.06 |
| RANBP3 | chr19 | NM_007320,NM_003624,NM_007322 | 50.06 |
| GLIPR2 | chr9 | NM_022343 | 50.06 |
| CYBRD1 | chr2 | NM_024843,NM_001127383 | 50.06 |
| GATA3 | chr10 | NM_002051,NM_001002295 | 50.05 |
| GPR137B | chr1 | NM_003272 | 50.05 |
| FER | chr5 | NM_005246 | 50.05 |
| PITPNC1 | chr17 | NM_012417,NM_181671 | 50.05 |
| USP47 | chr11 | NM_017944 | 50.05 |
| MDGA1 | chr6 | NM_153487 | 50.05 |
| RUNX1T1 | chr8 | NM_175635 | 50.05 |
| HCN1 | chr5 | NM_021072 | 50.04 |
| PROKR1 | chr2 | NM_138964 | 50.04 |
| GABPB1 | chr15 | NM_002041,NM_005254,NR_024490,NR_026891,NM_016655,NM_016654,NM_181427 | 50.03 |
| PLXDC1 | chr17 | NM_020405 | 50.03 |
| ACYP2 | chr2 | NM_138448 | 50.03 |
| UXT | chrX | NM_004182,NM_153477,NR_027444,NR_028119 | 50.02 |
| TNFSF10 | chr3 | NM_003810 | 50.01 |
| LGI2 | chr4 | NM_018176 | 50.01 |
| SLC35G1 | chr10 | NM_153226,NM_001134658 | 50.01 |
| APLP1 | chr19 | NM_001024807,NM_005166 | 50 |
| HMX2 | chr10 | NM_005519 | 50 |
| VSX2 | chr14 | NM_182894 | 50 |
| CARHSP1 | chr16 | NM_001042476 | 49.99 |
| GAD2 | chr10 | NM_001134366,NM_000818 | 49.99 |
| TLL2 | chr10 | NM_012465 | 49.99 |
| PVRL2 | chr19 | NM_001042724,NM_002856 | 49.98 |
| SYBU | chr8 | NM_001099754,NM_001099753,NM_001099755,NM_001099752,NM_001099751 | 49.98 |
| SCG3 | chr15 | NM_013243,NM_001165257 | 49.98 |
| FERMT1 | chr20 | NM_017671 | 49.98 |
| ANKRD34C | chr15 | NM_001146341 | 49.97 |
| POU4F1 | chr13 | NM_006237 | 49.97 |
| RAB32 | chr6 | NM_006834 | 49.97 |
| UBTD2 | chr5 | NM_152277 | 49.97 |
| MIR503HG | chrX | NR_024607,NR_030228 | 49.97 |
| CDC20B | chr5 | NM_152623,NM_001145734,NM_001170402,NR_031572 | 49.95 |
| SEC1P | chr19 | NR_004401 | 49.95 |
| GABRA3 | chrX | NM_000808 | 49.94 |
| CCND1 | chr11 | NM_053056 | 49.94 |
| ACE | chr17 | NM_000789 | 49.93 |
| HOXB5 | chr17 | NM_002147,NR_033204,NR_033203,NR_033205 | 49.93 |
| KLF10 | chr8 | NM_001032282 | 49.92 |
| BCAM | chr19 | NM_005581,NM_001013257 | 49.92 |
| HOXA2 | chr7 | NM_006735 | 49.92 |
| BLOC1S6 | chr15 | NM_012388 | 49.92 |
| NEUROD4 | chr12 | NM_021191 | 49.92 |
| AMOT | chrX | NM_133265 | 49.92 |
| ACVRL1 | chr12 | NM_000020 | 49.91 |
| MEIS1 | chr2 | NM_002398 | 49.91 |
| U2AF1 | chr21 | NM_006758,NM_001025204,NM_001025203 | 49.91 |
| PTDSS1 | chr8 | NM_014754 | 49.91 |
| AFTPH | chr2 | NM_017657,NM_203437,NM_001002243 | 49.91 |
| CTTNBP2 | chr7 | NM_033427 | 49.91 |
| SLC24A4 | chr14 | NM_153646,NM_153648,NM_153647 | 49.91 |
| GSC | chr14 | NM_173849 | 49.91 |
| CHRDL1 | chrX | NM_001143981,NM_001143982,NM_145234,NM_001143983 | 49.9 |
| FOSL1 | chr11 | NM_005438 | 49.9 |
| SDR16C5 | chr8 | NM_138969 | 49.9 |
| STXBP5L | chr3 | NM_014980 | 49.89 |
| C3orf58 | chr3 | NM_173552 | 49.89 |
| BSX | chr11 | NM_001098169 | 49.88 |
| BRD3 | chr9 | NM_007371 | 49.88 |
| TRIM44 | chr11 | NM_017583 | 49.88 |
| CHSY3 | chr5 | NM_175856 | 49.88 |
| NTF3 | chr12 | NM_001102654 | 49.87 |
| FEZF2 | chr3 | NM_018008 | 49.87 |
| JMY | chr5 | NM_152405 | 49.87 |
| SBSPON | chr8 | NM_153225 | 49.87 |
| MIER1 | chr1 | NM_001077704,NM_001077701 | 49.86 |
| ERGIC2 | chr12 | NM_016570 | 49.86 |
| NTN4 | chr12 | NM_021229 | 49.85 |
| FOXQ1 | chr6 | NM_033260 | 49.85 |
| MMAA | chr4 | NM_172250 | 49.85 |
| FAM19A4 | chr3 | NM_182522,NM_001005527 | 49.85 |
| RBM39 | chr20 | NM_004902,NM_184234 | 49.84 |
| KLF10 | chr8 | NM_005655,NM_001032282 | 49.84 |
| SNW1 | chr14 | NM_012245,NM_001173978,NM_174943 | 49.84 |
| NFKBIZ | chr3 | NM_031419 | 49.84 |
| GPM6B | chrX | NM_001001994 | 49.83 |
| NR2F6 | chr19 | NM_005234 | 49.83 |
| CDKN2AIP | chr4 | NM_017632 | 49.83 |
| G2E3 | chr14 | NM_017769 | 49.83 |
| DPH6 | chr15 | NM_080650,NM_001141972 | 49.83 |
| ARX | chrX | NM_139058 | 49.83 |
| TNRC18 | chr7 | NM_001080495 | 49.82 |
| TCF7L2 | chr10 | NM_001146284,NM_001146274,NM_030756,NM_001146283,NM_001146286,NM_001146285 | 49.82 |
| PTMA | chr2 | NM_002823,NM_001099285 | 49.82 |
| CFL1 | chr11 | NM_005507,NM_025128 | 49.82 |
| KRT222 | chr17 | NM_152349 | 49.82 |
| YTHDF3 | chr8 | NM_152758 | 49.82 |
| BDNF | chr11 | NM_170732,NM_001143807,NM_001143806,NM_001143805 | 49.82 |
| PPP3CA | chr4 | NM_001130691,NM_000944,NM_001130692 | 49.81 |
| FNDC1 | chr6 | NM_032532 | 49.81 |
| STARD8 | chrX | NM_001142504 | 49.8 |
| VSTM2B | chr19 | NM_001146339 | 49.8 |
| HIST1H2AB | chr6 | NM_003513,NM_003537 | 49.8 |
| ARHGEF18 | chr19 | NM_015318 | 49.8 |
| SPRY1 | chr4 | NM_199327,NM_005841 | 49.8 |
| KIAA1755 | chr20 | NM_001029864 | 49.79 |
| STAG2 | chrX | NM_001042751,NM_001042749,NM_006603,NM_001042750 | 49.79 |
| PHACTR2 | chr6 | NM_001100166,NM_014721 | 49.78 |
| MRPL36 | chr5 | NM_032479,NM_004553 | 49.78 |
| PCDH11X | chrX | NM_032967 | 49.78 |
| KATNAL1 | chr13 | NM_001014380,NM_032116 | 49.77 |
| ANK3 | chr10 | NM_001149 | 49.77 |
| NEUROG2 | chr4 | NM_024019 | 49.77 |
| RNF19A | chr8 | NM_183419 | 49.77 |
| PDE4C | chr19 | NM_001098818,NM_001098819 | 49.76 |
| SQLE | chr8 | NM_003129 | 49.76 |
| ATG2B | chr14 | NM_018036 | 49.76 |
| SYT9 | chr11 | NM_175733 | 49.76 |
| CHTF8 | chr16 | NM_001039690,NM_001040146,NR_033227,NM_032830 | 49.75 |
| GNAI1 | chr7 | NM_002069 | 49.75 |
| ITGAL | chr16 | NM_002209,NM_001114380 | 49.75 |
| EPB41L4B | chr9 | NM_019114,NM_018424 | 49.75 |
| PDZD4 | chrX | NM_032512 | 49.75 |
| CREBZF | chr11 | NM_001039618,NR_028024,NR_028026,NR_028025,NR_028027 | 49.74 |
| TMEM171 | chr5 | NM_001161342,NM_173490 | 49.74 |
| ZNF24 | chr18 | NM_006965 | 49.74 |
| NNT | chr5 | NM_012343,NM_182977 | 49.74 |
| FXYD7 | chr19 | NM_022006 | 49.74 |
| TBC1D10A | chr22 | NM_031937 | 49.74 |
| PGBD1 | chr6 | NM_032507 | 49.74 |
| ASH1L-AS1 | chr1 | NR_027023,NM_018489 | 49.74 |
| PGM1 | chr1 | NM_002633,NM_001172819 | 49.73 |
| FADS1 | chr11 | NM_013402,NR_031729 | 49.73 |
| AGPAT3 | chr21 | NM_020132 | 49.73 |
| ZFAS1 | chr20 | NR_003604,NR_003605,NM_021035,NR_003606,NR_002433,NR_003695,NR_031660 | 49.73 |
| BARD1 | chr2 | NM_000465 | 49.72 |
| WEE1 | chr11 | NM_001143976,NM_003390 | 49.72 |
| GPR137 | chr11 | NM_001177358,NM_032989,NM_004322,NM_001170880,NM_001170881,NM_020155 | 49.72 |
| LHX2 | chr9 | NM_004789 | 49.72 |
| NME6 | chr3 | NM_005793 | 49.72 |
| FIBP | chr11 | NM_198897,NM_004214,NM_006848 | 49.72 |
| HNRNPUL2 | chr11 | NM_001079559,NM_173810 | 49.71 |
| ZNF674 | chrX | NM_001146291,NR_015378,NM_001039891 | 49.71 |
| STRAP | chr12 | NM_007178 | 49.71 |
| TRAM1 | chr8 | NM_014294 | 49.71 |
| ABCD3 | chr1 | NM_002858,NM_001122674 | 49.7 |
| KLF9 | chr9 | NM_001206 | 49.69 |
| RGS10 | chr10 | NM_001005339 | 49.68 |
| NUCB2 | chr11 | NM_005013 | 49.68 |
| SARS | chr1 | NM_006513 | 49.68 |
| ZNF428 | chr19 | NM_182498 | 49.68 |
| SNORD59A | chr12 | NR_002737,NR_003046,NM_001686 | 49.68 |
| ITPRIPL2 | chr16 | NM_001034841,NR_028028 | 49.67 |
| FAM168A | chr11 | NM_015159 | 49.67 |
| WT1 | chr11 | NM_024424,NM_024426,NM_000378,NR_023920,NM_024425 | 49.67 |
| SIX5 | chr19 | NM_175875 | 49.67 |
| SLC4A11 | chr20 | NM_001174090,NM_032034,NM_001174089 | 49.66 |
| AASS | chr7 | NM_005763 | 49.66 |
| TLX1NB | chr10 | NM_001085398,NM_005521 | 49.65 |
| ARID4A | chr14 | NM_002892,NR_029434,NR_029435,NM_023001,NM_023000 | 49.65 |
| TLR5 | chr1 | NM_003268 | 49.65 |
| AGPAT9 | chr4 | NM_032717 | 49.65 |
| MARVELD2 | chr5 | NM_001038603 | 49.64 |
| PSMA4 | chr15 | NM_001102668,NM_001102667,NM_002789 | 49.64 |
| BAZ2A | chr12 | NM_013449 | 49.64 |
| RGS3 | chr9 | NM_134427 | 49.64 |
| AKAP12 | chr6 | NM_005100 | 49.63 |
| RNF44 | chr5 | NM_014901 | 49.63 |
| SP1 | chr12 | NM_138473,NM_003109 | 49.63 |
| PARD3B | chr2 | NM_152526,NM_057177,NM_205863 | 49.63 |
| SATB2-AS1 | chr2 | NR_026830,NM_015265 | 49.63 |
| GABRB2 | chr5 | NM_000813,NM_021911 | 49.62 |
| EML6 | chr2 | NM_001039753 | 49.62 |
| ATXN1 | chr6 | NM_001128164,NM_000332 | 49.62 |
| EN2 | chr7 | NM_001427 | 49.62 |
| DOCK11 | chrX | NM_144658 | 49.62 |
| SLC10A5 | chr8 | NM_001010893 | 49.61 |
| PDE4B | chr1 | NM_001037339 | 49.61 |
| GNAQ | chr9 | NM_002072 | 49.61 |
| NAA38 | chr7 | NM_016200 | 49.61 |
| ZFP64 | chr20 | NM_018197,NM_022088,NM_199426,NM_199427 | 49.61 |
| ZBTB2 | chr6 | NM_020861 | 49.61 |
| MRM1 | chr17 | NM_024864 | 49.61 |
| MYCNOS | chr2 | NR_026766,NM_005378 | 49.61 |
| ONECUT1 | chr15 | NM_004498 | 49.6 |
| TRIM52 | chr5 | NM_032765 | 49.6 |
| PKIB | chr6 | NM_181795,NM_032471 | 49.6 |
| TBX3 | chr12 | NM_005996,NM_016569 | 49.59 |
| KCNK10 | chr14 | NM_138317 | 49.59 |
| HELT | chr4 | NM_001029887 | 49.58 |
| TBC1D14 | chr4 | NM_001113361 | 49.58 |
| HIST1H2BL | chr6 | NM_003519,NM_003509 | 49.58 |
| NRXN1 | chr2 | NM_138735 | 49.58 |
| ITGB3 | chr17 | NM_000212 | 49.57 |
| PHB2 | chr12 | NM_001144831,NM_007273,NM_006331 | 49.57 |
| SLC6A1 | chr3 | NM_003042 | 49.57 |
| PCGF2 | chr17 | NM_007144 | 49.57 |
| YPEL2 | chr17 | NM_001005404 | 49.56 |
| AGAP2 | chr12 | NM_001122772 | 49.56 |
| GRAMD1A | chr19 | NM_020895,NM_001136199 | 49.56 |
| SLC35F3 | chr1 | NM_173508 | 49.56 |
| ATP6V1A | chr3 | NM_001690,NM_025146 | 49.55 |
| PKDREJ | chr22 | NM_006071 | 49.55 |
| ZBTB43 | chr9 | NM_014007,NM_001135776 | 49.55 |
| HSPA12A | chr10 | NM_025015 | 49.55 |
| NKD1 | chr16 | NM_033119 | 49.55 |
| CEP85L | chr6 | NM_206921,NM_001042475 | 49.55 |
| CA5BP1 | chrX | NR_026551 | 49.55 |
| ZNF816 | chr19 | NM_001031665 | 49.54 |
| SYNCRIP | chr6 | NM_001159677,NM_001159674,NM_001159676,NM_001159675,NM_006372,NM_001159673 | 49.54 |
| TRIP10 | chr19 | NM_004240,NM_001080452 | 49.54 |
| VAX1 | chr10 | NM_199131,NM_001112704 | 49.54 |
| CDK8 | chr13 | NM_001260 | 49.53 |
| SUFU | chr10 | NM_016169,NM_005736 | 49.53 |
| CPEB2 | chr4 | NM_001177383,NM_001177384,NM_182485,NM_182646,NM_001177381,NM_001177382 | 49.52 |
| RASGRF2 | chr5 | NM_006909 | 49.52 |
| KIAA0226 | chr3 | NM_014687 | 49.52 |
| KIAA1210 | chrX | NM_020721 | 49.52 |
| PDE5A | chr4 | NM_033430,NM_001083,NM_033437 | 49.52 |
| TP53I13 | chr17 | NM_138349,NM_198147 | 49.52 |
| LYSMD2 | chr15 | NM_153374 | 49.52 |
| ECEL1P2 | chr2 | NR_028501 | 49.52 |
| ITPR3 | chr6 | NM_002224 | 49.51 |
| TFEB | chr6 | NM_007162 | 49.51 |
| ATF6 | chr1 | NM_007348 | 49.51 |
| E2F8 | chr11 | NM_024680 | 49.51 |
| RASGEF1B | chr4 | NM_152545 | 49.51 |
| C17orf105 | chr17 | NM_001136483,NM_004090 | 49.5 |
| EI24 | chr11 | NM_004879,NM_001007277 | 49.5 |
| POU2F3 | chr11 | NM_014352 | 49.5 |
| TRAPPC8 | chr18 | NM_014939 | 49.5 |
| PLCD1 | chr3 | NM_006225 | 49.49 |
| NOS1AP | chr1 | NM_014697,NM_001164757 | 49.49 |
| DSC3 | chr18 | NM_024423,NM_001941 | 49.49 |
| FAM105B | chr5 | NM_138348 | 49.49 |
| HMGB2 | chr4 | NM_001130689,NM_001130688,NM_002129 | 49.48 |
| LPXN | chr11 | NM_001143995,NR_024091,NM_053023 | 49.48 |
| NOVA2 | chr19 | NM_002516 | 49.48 |
| RPS6KA2 | chr6 | NM_021135 | 49.48 |
| HAGHL | chr16 | NM_032304 | 49.48 |
| GAS5 | chr1 | NR_002578,NR_003942,NR_003944,NR_003939,NR_003943,NR_002750,NR_003941,NR_002579,NM_032522,NM_001122770 | 49.48 |
| PEPD | chr19 | NM_000285,NM_001166057,NM_001166056 | 49.47 |
| CH25H | chr10 | NM_003956 | 49.47 |
| TRIP10 | chr19 | NM_004240 | 49.47 |
| ITGB1BP1 | chr2 | NM_004763,NM_016207,NM_022334 | 49.47 |
| POU3F4 | chrX | NM_000307 | 49.46 |
| RAP1B | chr12 | NM_001010942,NM_015646 | 49.46 |
| SOX14 | chr3 | NM_004189 | 49.46 |
| FEZ1 | chr11 | NM_005103,NM_022549 | 49.46 |
| HNRNPU | chr1 | NM_031844,NM_004501 | 49.46 |
| CPE | chr4 | NM_001873 | 49.45 |
| SORBS3 | chr8 | NM_005775 | 49.45 |
| USP7 | chr16 | NM_003470 | 49.44 |
| FGF5 | chr4 | NM_004464,NM_033143 | 49.44 |
| SEPHS1 | chr10 | NM_012247 | 49.44 |
| FERD3L | chr7 | NM_152898 | 49.44 |
| NDUFB6 | chr9 | NM_182739,NM_002493 | 49.44 |
| NEDD1 | chr12 | NM_001135177,NM_001135176,NM_152905,NM_001135175 | 49.43 |
| TCF3 | chr19 | NM_001136139,NM_003200 | 49.43 |
| MYOD1 | chr11 | NM_002478 | 49.43 |
| HHEX | chr10 | NM_002729 | 49.43 |
| LSG1 | chr3 | NM_018385 | 49.43 |
| CALD1 | chr7 | NM_033140,NM_033139 | 49.43 |
| SLC36A4 | chr11 | NM_152313 | 49.43 |
| THYN1 | chr11 | NM_199298,NM_001037304,NM_199297,NM_001037305,NM_014174,NM_014384 | 49.43 |
| CELF2 | chr10 | NM_006561 | 49.42 |
| CCDC50 | chr3 | NM_178335,NM_174908,NM_198152 | 49.42 |
| TMEM200B | chr1 | NM_001003682,NM_001171868 | 49.41 |
| TFAP2A | chr6 | NM_001032280 | 49.41 |
| KIF3A | chr5 | NM_007054 | 49.41 |
| CUL9 | chr6 | NM_015089 | 49.41 |
| MYO3A | chr10 | NM_017433 | 49.41 |
| USE1 | chr19 | NM_018467 | 49.41 |
| SMAD2 | chr18 | NM_001003652,NM_005901,NM_001135937 | 49.4 |
| ETV1 | chr7 | NM_001163148,NM_001163149,NM_001163147,NM_004956 | 49.4 |
| SMAD6 | chr15 | NM_005585,NR_027654 | 49.4 |
| FZD10 | chr12 | NM_007197 | 49.4 |
| STRBP | chr9 | NM_018387,NR_033234,NM_001171137 | 49.4 |
| USP31 | chr16 | NM_020718 | 49.4 |
| CPLX2 | chr5 | NM_001008220 | 49.39 |
| SEPSECS | chr4 | NM_016955 | 49.39 |
| FYCO1 | chr3 | NM_024513 | 49.39 |
| DOCK5 | chr8 | NM_024940 | 49.39 |
| CACNB1 | chr17 | NM_000723,NM_199248,NM_199247 | 49.38 |
| HIC1 | chr17 | NM_001098202,NM_006497 | 49.38 |
| LMO2 | chr11 | NM_001142316,NM_001142315 | 49.38 |
| MN1 | chr22 | NM_002430 | 49.38 |
| QKI | chr6 | NM_006775,NM_206854,NM_206855,NM_206853 | 49.38 |
| EDEM1 | chr3 | NM_014674 | 49.38 |
| MEF2A | chr15 | NM_001171894,NM_005587,NM_001130926,NM_001130927 | 49.37 |
| CHRD | chr3 | NM_003741 | 49.37 |
| FLT3 | chr13 | NM_004119 | 49.37 |
| SCAF4 | chr21 | NM_020706,NM_001145444,NM_001145445 | 49.37 |
| CLK3 | chr15 | NM_001130028 | 49.36 |
| NBAS | chr2 | NM_015909 | 49.36 |
| NAP1L1 | chr12 | NM_139207,NM_004537 | 49.36 |
| CHMP4C | chr8 | NM_152284 | 49.36 |
|  | chr17 | NR_026899,NR_027487 | 49.36 |
| TBL1X | chrX | NM_005647 | 49.35 |
| VAT1L | chr16 | NM_020927 | 49.35 |
| DDX26B | chrX | NM_182540 | 49.35 |
| MIR1915 | chr10 | NR_031736 | 49.35 |
| GDNF | chr5 | NM_000514 | 49.34 |
| SORBS3 | chr8 | NM_001018003 | 49.34 |
| SPTSSB | chr3 | NM_001040100 | 49.33 |
| REPIN1 | chr7 | NM_001099696,NM_001099695,NM_013400 | 49.33 |
| TGFB2 | chr1 | NM_001135599,NM_003238 | 49.33 |
| LGR4 | chr11 | NM_018490 | 49.33 |
| TMEM116 | chr12 | NM_138341,NM_006817,NM_001034025 | 49.33 |
| PELI3 | chr11 | NM_001098510,NM_145065 | 49.32 |
| ERBB4 | chr2 | NM_005235,NM_001042599 | 49.32 |
| PCNT | chr21 | NM_006031,NM_058180 | 49.32 |
| WNT6 | chr2 | NM_006522 | 49.32 |
| RHBDL3 | chr17 | NM_138328 | 49.32 |
| ZNF805 | chr19 | NM_001023563,NM_001145078 | 49.31 |
| RHOD | chr11 | NM_014578 | 49.31 |
| CACNA1D | chr3 | NM_001128840,NM_001128839,NM_000720 | 49.3 |
| SYNJ2 | chr6 | NM_003898 | 49.3 |
| TMEM38B | chr9 | NM_018112 | 49.3 |
| FOXA2 | chr20 | NM_021784,NM_153675 | 49.3 |
| TIRAP | chr11 | NM_148910,NM_001039661 | 49.3 |
| IRF3 | chr19 | NM_001571,NM_001040668,NM_138639 | 49.29 |
| DDX5 | chr17 | NM_004396,NM_138363 | 49.29 |
| RBM15B | chr3 | NM_013286 | 49.29 |
| VTA1 | chr6 | NM_016485 | 49.29 |
| EPS8L1 | chr19 | NM_017729 | 49.29 |
| LINC00261 | chr20 | NR_001558 | 49.29 |
| GPD2 | chr2 | NM_000408,NM_001083112 | 49.28 |
| C2CD4B | chr15 | NM_001007595 | 49.28 |
| PHKB | chr16 | NM_001031835,NM_030790,NM_000293 | 49.28 |
| ONECUT3 | chr19 | NM_001080488 | 49.28 |
| GSK3B | chr3 | NM_001146156,NM_002093 | 49.28 |
| ATXN10 | chr22 | NM_001167621,NM_013236 | 49.28 |
| MIR1227 | chr19 | NR_031596,NM_018049,NM_007165 | 49.28 |
| MYCBP | chr1 | NM_012333 | 49.27 |
| CYHR1 | chr8 | NM_032687,NM_001129888,NM_145754 | 49.27 |
| EMX2OS | chr10 | NR_002791 | 49.27 |
| MXI1 | chr10 | NM_001008541 | 49.26 |
| U2SURP | chr3 | NM_001080415 | 49.26 |
| ST6GAL2 | chr2 | NM_001142351,NM_001142352,NM_032528 | 49.26 |
| BIVM | chr13 | NM_017693,NM_001159596,NM_024089 | 49.26 |
| ZNF367 | chr9 | NM_153695 | 49.26 |
| P4HTM | chr3 | NM_177939,NM_177938 | 49.26 |
| LAYN | chr11 | NM_178834 | 49.26 |
| PAK2 | chr3 | NM_002577 | 49.25 |
| TGFBR3 | chr1 | NM_003243 | 49.25 |
| RUNX3 | chr1 | NM_004350 | 49.25 |
| ABL1 | chr9 | NM_005157 | 49.25 |
| CORO1C | chr12 | NM_014325 | 49.25 |
| MEX3C | chr18 | NM_016626 | 49.25 |
| TLX3 | chr5 | NM_021025 | 49.25 |
| TMEM87B | chr2 | NM_032824 | 49.25 |
| ALDH16A1 | chr19 | NM_153329,NM_017916,NM_001145396 | 49.25 |
| ABHD12B | chr14 | NM_181814,NM_181533 | 49.25 |
| SS18 | chr18 | NM_001007559,NM_005637 | 49.24 |
| C11orf96 | chr11 | NM_001145033 | 49.24 |
| CDH7 | chr18 | NM_004361,NM_033646 | 49.24 |
| FZD7 | chr2 | NM_003507 | 49.23 |
| GLCE | chr15 | NM_015554 | 49.23 |
| GPN3 | chr12 | NM_016301,NM_001164373,NM_013300,NM_001164372 | 49.23 |
| PAX5 | chr9 | NM_016734 | 49.23 |
| PCDHB6 | chr5 | NM_018939 | 49.23 |
| SPHK1 | chr17 | NM_182965,NM_021972,NM_001142601 | 49.23 |
| SNCB | chr5 | NM_001001502,NM_003085,NM_001099408 | 49.22 |
| BAG5 | chr14 | NM_001015048,NM_004873,NM_001015049,NM_032374 | 49.22 |
| KIAA0895L | chr16 | NM_001040715 | 49.22 |
| URGCP | chr7 | NM_001077664,NM_015983 | 49.22 |
| ALDH1A2 | chr15 | NM_003888,NM_170696 | 49.22 |
| ABCD2 | chr12 | NM_005164 | 49.22 |
| KIAA0247 | chr14 | NM_014734 | 49.22 |
| TMEM260 | chr14 | NM_017799 | 49.22 |
| SLC22A15 | chr1 | NM_018420 | 49.22 |
| SLC25A40 | chr7 | NM_018843,NM_006716 | 49.22 |
| KIAA1524 | chr3 | NM_020890,NM_014648 | 49.22 |
| CACHD1 | chr1 | NM_020925 | 49.22 |
| GRIN3A | chr9 | NM_133445 | 49.22 |
| FAM124A | chr13 | NM_145019 | 49.22 |
| MANEAL | chr1 | NM_152496,NM_001031740,NM_001113482 | 49.22 |
| DUSP4 | chr8 | NM_001394 | 49.21 |
| IRF1 | chr5 | NM_002198 | 49.21 |
| PFDN4 | chr20 | NM_002623 | 49.21 |
| CYTH3 | chr7 | NM_004227 | 49.21 |
| FOXC2 | chr16 | NM_005251 | 49.21 |
| ZC3H12C | chr11 | NM_033390 | 49.21 |
| OMA1 | chr1 | NM_145243 | 49.21 |
| MORF4L2 | chrX | NM_001142424,NM_012286,NM_001142426,NM_001142418,NM_001142421,NM_001142419,NM_001142430,NM_001142425,NM_001142429,NM_001142423,NM_001142420,NM_001142432,NM_001142428,NM_001142427,NM_001142431,NM_001142422 | 49.2 |
| RFTN1 | chr3 | NM_015150 | 49.2 |
| GPATCH1 | chr19 | NM_018025 | 49.2 |
| SDF2L1 | chr22 | NM_022044 | 49.2 |
| SOX7 | chr8 | NM_031439 | 49.2 |
| CLTA | chr9 | NM_001076677,NM_007096,NM_001833 | 49.19 |
| CORIN | chr4 | NM_006587 | 49.19 |
| PSMF1 | chr20 | NM_006814 | 49.19 |
| PITX1 | chr5 | NM_002653 | 49.18 |
| PHOX2A | chr11 | NM_005169 | 49.18 |
| MNX1 | chr7 | NM_005515,NM_001165255 | 49.18 |
| TRIB3 | chr20 | NM_021158 | 49.18 |
| IL17RC | chr3 | NM_153461,NM_153460,NM_032732 | 49.18 |
| ZNF189 | chr9 | NM_197977,NM_019051,NM_003452 | 49.18 |
| SKIDA1 | chr10 | NM_207371 | 49.18 |
| TCHP | chr12 | NM_001143852,NM_032300 | 49.17 |
| EIF3I | chr1 | NM_003757,NM_019118 | 49.17 |
| EMILIN2 | chr18 | NM_032048 | 49.17 |
| FAM221A | chr7 | NM_199136,NM_001127364,NM_001127365 | 49.17 |
| CHRM2 | chr7 | NM_001006632,NM_001006631,NM_000739,NM_001006629,NM_001006626,NM_001006628 | 49.16 |
| MNAT1 | chr14 | NM_002431 | 49.16 |
| PREP | chr6 | NM_002726 | 49.16 |
| SLC25A24 | chr1 | NM_013386 | 49.16 |
| ARSJ | chr4 | NM_024590 | 49.16 |
| HOXA11-AS | chr7 | NR_002795,NM_005523 | 49.16 |
| NCOA3 | chr20 | NM_001174088,NM_001174087,NM_006534,NM_181659 | 49.15 |
| S1PR2 | chr19 | NM_004230 | 49.15 |
| POU3F3 | chr2 | NM_006236 | 49.15 |
| SERTAD4 | chr1 | NM_019605,NR_024337 | 49.15 |
| ARNTL2 | chr12 | NM_020183 | 49.15 |
| IRX1 | chr5 | NM_024337 | 49.15 |
| SLCO5A1 | chr8 | NM_030958,NM_001146008,NM_001146009 | 49.15 |
| ZNF488 | chr10 | NM_153034 | 49.15 |
| TTLL3 | chr3 | NM_001025930 | 49.14 |
| PDLIM3 | chr4 | NM_001114107,NM_014476 | 49.14 |
| C3orf80 | chr3 | NM_001168214 | 49.14 |
| CLPTM1 | chr19 | NM_001294 | 49.14 |
| DGKZ | chr11 | NM_003646,NM_201533 | 49.14 |
| SOX3 | chrX | NM_005634 | 49.14 |
| SRPX | chrX | NM_006307,NM_001170752,NM_001170750,NM_001170751 | 49.14 |
| IMPA2 | chr18 | NM_014214 | 49.14 |
| AKAP11 | chr13 | NM_016248 | 49.14 |
| SPTY2D1 | chr11 | NM_194285 | 49.14 |
| SNORD61 | chrX | NR_002735,NM_001164803,NR_028477,NR_028476,NM_002139 | 49.14 |
| RBMX | chrX | NR_002735,NM_001164803,NR_028477,NR_028476,NM_002139 | 49.14 |
| N4BP2L1 | chr13 | NM_001079691,NM_052818 | 49.13 |
| FOXO1 | chr13 | NM_002015 | 49.13 |
| FAM115A | chr7 | NM_014719 | 49.13 |
| TEX10 | chr9 | NM_017746,NM_001161584 | 49.13 |
| MFSD6L | chr17 | NM_152599 | 49.13 |
| AKR1A1 | chr1 | NM_153326,NM_006066 | 49.13 |
| SKP1 | chr5 | NM_170679,NM_006930 | 49.13 |
| RHEB | chr7 | NM_005614 | 49.12 |
| MND1 | chr4 | NM_032117 | 49.12 |
| FAM222A | chr12 | NM_032829 | 49.12 |
| HAGH | chr16 | NM_001040427,NM_001018104,NM_005326,NM_031208,NM_001142398 | 49.11 |
| SPATA5L1 | chr15 | NM_024063,NR_027635 | 49.11 |
| SPAG16 | chr2 | NM_024532,NM_001025436 | 49.11 |
| KIAA0368 | chr9 | NM_001080398 | 49.1 |
| PAX6 | chr11 | NM_001604,NM_000280 | 49.1 |
| ANKRD11 | chr16 | NM_013275 | 49.1 |
| PHF7 | chr3 | NM_016483,NM_004656,NM_173341 | 49.1 |
| CHCHD4 | chr3 | NM_144636,NM_001098502,NM_024334 | 49.1 |
| KIAA1239 | chr4 | NM_001144990 | 49.09 |
| ERBB2IP | chr5 | NM_001006600,NM_018695 | 49.08 |
| PRR7 | chr5 | NM_001174101,NM_030567,NM_001174102 | 49.08 |
| SMAD7 | chr18 | NM_005904 | 49.08 |
| SLC7A3 | chrX | NM_032803,NM_001048164 | 49.08 |
| COX20 | chr1 | NM_198076 | 49.08 |
| MAOB | chrX | NM_000898 | 49.07 |
| FAM135A | chr6 | NM_001105531,NM_020819 | 49.07 |
| AGO2 | chr8 | NM_012154,NM_001164623 | 49.07 |
| DKK2 | chr4 | NM_014421 | 49.07 |
| EBF3 | chr10 | NM_001005463 | 49.06 |
| LTBP4 | chr19 | NM_001042545 | 49.06 |
| CDX2 | chr13 | NM_001265 | 49.06 |
| NMT2 | chr10 | NM_004808 | 49.06 |
| APPL2 | chr12 | NM_018171 | 49.06 |
| KCNJ13 | chr2 | NM_001172416,NM_002242,NM_001172417 | 49.05 |
| NDST2 | chr10 | NM_003635 | 49.05 |
| FKBP1B | chr2 | NM_004116,NM_054033 | 49.05 |
| PFKFB3 | chr10 | NM_004566 | 49.05 |
| DCUN1D4 | chr4 | NM_015115,NM_001040402 | 49.05 |
| PLXNB1 | chr3 | NM_002673,NM_001130082 | 49.04 |
| ZNF131 | chr5 | NM_003432 | 49.04 |
| GYLTL1B | chr11 | NM_152312 | 49.04 |
| C17orf58 | chr17 | NM_181656,NM_181655 | 49.04 |
| CLCN5 | chrX | NM_000084 | 49.03 |
| RRM1 | chr11 | NM_001033 | 49.03 |
| RRBP1 | chr20 | NM_001042576,NM_004587 | 49.03 |
| FMR1 | chrX | NM_002024 | 49.03 |
| MAP3K1 | chr5 | NM_005921 | 49.03 |
| SLC17A6 | chr11 | NM_020346 | 49.03 |
| PCBP4 | chr3 | NM_020418,NM_001174100,NM_033008 | 49.03 |
| MAP6D1 | chr3 | NM_024871 | 49.03 |
| LIMD2 | chr17 | NM_030576 | 49.03 |
| L3MBTL3 | chr6 | NM_032438,NM_001007102 | 49.03 |
| GGA1 | chr22 | NM_001001561,NM_001001560,NM_001172688,NM_001172687,NM_013365 | 49.02 |
| ZNF267 | chr16 | NM_003414 | 49.02 |
| BNIP2 | chr15 | NM_004330 | 49.02 |
| SGTB | chr5 | NM_019072,NM_020726 | 49.02 |
| HOXC12 | chr12 | NM_173860 | 49.02 |
| PLEKHA1 | chr10 | NM_001001974 | 49.01 |
| PSMC5 | chr17 | NM_002805,NM_017647 | 49.01 |
| CUL3 | chr2 | NM_003590 | 49.01 |
| E2F1 | chr20 | NM_005225 | 49.01 |
| VCL | chr10 | NM_014000,NM_003373 | 49.01 |
| NTNG1 | chr1 | NM_014917,NM_001113228,NM_001113226 | 49.01 |
| PPP1R14C | chr6 | NM_030949 | 49.01 |
| LARGE | chr22 | NM_133642,NM_004737 | 49 |
| GRIA1 | chr5 | NM_001114183,NM_000827 | 48.99 |
| GPR75-ASB3 | chr2 | NM_001164165,NM_006794 | 48.99 |
| FOXF2 | chr6 | NM_001452 | 48.99 |
| SSH2 | chr17 | NM_033389,NM_001145053 | 48.99 |
| NDUFAF3 | chr3 | NM_199074,NR_029948,NM_001009996,NR_029690 | 48.99 |
| BTRC | chr10 | NM_003939,NM_033637 | 48.98 |
| ARPC5L | chr9 | NM_030978 | 48.98 |
| KBTBD7 | chr13 | NM_032138 | 48.98 |
| NAF1 | chr4 | NM_138386,NM_001128931 | 48.98 |
| TGIF1 | chr18 | NM_170695,NM_003244,NM_173209,NM_173208,NM_173210 | 48.98 |
| CHRM1 | chr11 | NM_000738 | 48.97 |
| ZBTB21 | chr21 | NM_001098402,NM_020727,NM_001098403 | 48.97 |
| MLLT10 | chr10 | NM_004641,NM_001009569 | 48.97 |
| OXR1 | chr8 | NM_018002 | 48.97 |
| MSL2 | chr3 | NM_018133 | 48.97 |
| VANGL2 | chr1 | NM_020335 | 48.97 |
| SPRYD7 | chr13 | NM_020456,NR_023351,NM_001127482 | 48.97 |
| ZNF692 | chr1 | NM_001136036,NM_017865 | 48.96 |
| SH3BP5 | chr3 | NM_004844 | 48.96 |
| TM2D2 | chr8 | NM_078473,NR_027639,NM_003816,NR_027638,NM_031940,NM_001024381,NM_001024380,NR_027878 | 48.96 |
| MID1IP1 | chrX | NM_001098790,NM_001098791 | 48.95 |
| PAM | chr5 | NM_001177306,NM_000919,NM_138822,NM_138766,NR_033440,NM_138821 | 48.95 |
| TDGF1 | chr3 | NM_003212 | 48.95 |
| ASXL2 | chr2 | NM_018263 | 48.95 |
| C1QTNF2 | chr5 | NM_031908 | 48.95 |
| TTC21A | chr3 | NM_001105513,NM_031899,NM_145755 | 48.94 |
| UNG | chr12 | NM_003362,NM_080911 | 48.94 |
| NFKB1 | chr4 | NM_003998,NM_001165412 | 48.94 |
| GBF1 | chr10 | NM_004193 | 48.94 |
| NREP | chr5 | NM_001142481,NM_004772,NM_001142483,NM_001142482,NM_001142479,NM_001142480,NM_001142478,NM_001142477,NM_001142476 | 48.93 |
| CD1D | chr1 | NM_001766 | 48.93 |
| PIGL | chr17 | NM_004278 | 48.93 |
| LBX1 | chr10 | NM_006562 | 48.93 |
| MBNL3 | chrX | NM_001170704 | 48.92 |
| DECR1 | chr8 | NM_001359 | 48.92 |
| DCHS1 | chr11 | NM_003737 | 48.92 |
| HOXA5 | chr7 | NM_019102 | 48.92 |
| MBD6 | chr12 | NM_052897 | 48.92 |
| MARK3 | chr14 | NM_001128921,NM_001128919,NM_002376,NM_001128920,NM_001128918 | 48.91 |
| BCL2L1 | chr20 | NM_001191,NM_138578 | 48.91 |
| LRBA | chr4 | NM_006726 | 48.91 |
| SV2C | chr5 | NM_014979 | 48.9 |
| BRIX1 | chr5 | NM_018321,NR_026591,NM_002853 | 48.9 |
| STX12 | chr1 | NM_177424 | 48.9 |
| CRMP1 | chr4 | NM_001014809 | 48.89 |
| ZIC1 | chr3 | NM_003412 | 48.89 |
| RGS20 | chr8 | NM_003702 | 48.89 |
| INPP5A | chr10 | NM_005539 | 48.89 |
| ARHGEF40 | chr14 | NM_018071 | 48.89 |
| TOMM40L | chr1 | NM_032174 | 48.89 |
| DENND1B | chr1 | NM_144977,NM_001142795 | 48.89 |
| TJP1 | chr15 | NM_175610,NM_003257 | 48.89 |
| PLEKHG1 | chr6 | NM_001029884 | 48.88 |
| DARC | chr1 | NM_001122951,NM_002036 | 48.88 |
| CDC42EP5 | chr19 | NM_145057 | 48.88 |
| CRYZL1 | chr21 | NM_145858,NM_001001132,NM_003024 | 48.88 |
